# Minimally Mutated HIV-1 Broadly Neutralizing Antibodies to Guide Reductionist Vaccine Design

**DOI:** 10.1371/journal.ppat.1005815

**Published:** 2016-08-25

**Authors:** Joseph G. Jardine, Devin Sok, Jean-Philippe Julien, Bryan Briney, Anita Sarkar, Chi-Hui Liang, Erin A. Scherer, Carole J. Henry Dunand, Yumiko Adachi, Devan Diwanji, Jessica Hsueh, Meaghan Jones, Oleksandr Kalyuzhniy, Michael Kubitz, Skye Spencer, Matthias Pauthner, Karen L. Saye-Francisco, Fabian Sesterhenn, Patrick C. Wilson, Denise M. Galloway, Robyn L. Stanfield, Ian A. Wilson, Dennis R. Burton, William R. Schief

**Affiliations:** 1 Department of Immunology and Microbial Science, The Scripps Research Institute, La Jolla, California, United States of America; 2 IAVI Neutralizing Antibody Center, The Scripps Research Institute, La Jolla, California, United States of America; 3 Center for HIV/AIDS Vaccine Immunology and Immunogen Discovery, The Scripps Research Institute, La Jolla, California, United States of America; 4 Department of Biochemistry, University of Washington, Seattle, Washington, United States of America; 5 International AIDS Vaccine Initiative, New York, New York, United States of America; 6 Department of Integrative Structural and Computational Biology, The Scripps Research Institute, La Jolla, California, United States of America; 7 Program in Molecular Structure and Function, The Hospital for Sick Children Research Institute and Departments of Biochemistry and Immunology, University of Toronto, Toronto, Ontario, Canada; 8 Human Biology Division, Fred Hutchinson Cancer Research Center, Seattle, Washington, United States of America; 9 Department of Medicine, Section of Rheumatology, The Knapp Center for Lupus and Immunology Research, The University of Chicago, Chicago, Illinois, United States of America; 10 Skaggs Institute for Chemical Biology, The Scripps Research Institute, La Jolla, California, United States of America; 11 Ragon Institute of MGH, MIT, and Harvard, Cambridge, Massachusetts, United States of America; University of Zurich, SWITZERLAND

## Abstract

An optimal HIV vaccine should induce broadly neutralizing antibodies (bnAbs) that neutralize diverse viral strains and subtypes. However, potent bnAbs develop in only a small fraction of HIV-infected individuals, all contain rare features such as extensive mutation, insertions, deletions, and/or long complementarity-determining regions, and some are polyreactive, casting doubt on whether bnAbs to HIV can be reliably induced by vaccination. We engineered two potent VRC01-class bnAbs that minimized rare features. According to a quantitative features frequency analysis, the set of features for one of these minimally mutated bnAbs compared favorably with all 68 HIV bnAbs analyzed and was similar to antibodies elicited by common vaccines. This same minimally mutated bnAb lacked polyreactivity in four different assays. We then divided the minimal mutations into spatial clusters and dissected the epitope components interacting with those clusters, by mutational and crystallographic analyses coupled with neutralization assays. Finally, by synthesizing available data, we developed a working-concept boosting strategy to select the mutation clusters in a logical order following a germline-targeting prime. We have thus developed potent HIV bnAbs that may be more tractable vaccine goals compared to existing bnAbs, and we have proposed a strategy to elicit them. This reductionist approach to vaccine design, guided by antibody and antigen structure, could be applied to design candidate vaccines for other HIV bnAbs or protective Abs against other pathogens.

## Author Summary

Many HIV vaccine design efforts aim to elicit so-called broadly neutralizing antibodies that bind and neutralize diverse strains and subtypes of the virus. However, these efforts are guided by very unusual antibodies isolated from HIV-infected individuals. These antibodies have rare features that limit their use as direct vaccine templates, because it is unlikely that any vaccine could consistently elicit similar antibodies. We engineered HIV broadly neutralizing antibodies that minimized these rare features and may therefore serve as better leads for HIV vaccine design. Antibodies generally gain affinity for their target epitope by accumulating mutations in a natural process of maturation. Figuring out how to use vaccines to elicit particular kinds of antibodies, with particular kinds of helpful mutations, is a major unsolved challenge for vaccine design. We were able to determine which mutations in our new antibodies are most important and which epitope structures are needed to induce those mutations. This analysis allowed us to deduce a logical strategy, which remains to be tested, for how to guide the maturation of these types of antibodies by vaccination. We propose that this reductionist approach to vaccine design, guided by molecular structure and engineering-oriented to allow for optimization, has promise for designing vaccines against HIV and many other pathogens.

## Introduction

Many antibodies capable of neutralizing a large fraction of circulating HIV isolates, broadly neutralizing antibodies (bnAbs), have been isolated from HIV-infected individuals [1–12]. Combinations of bnAbs targeting different epitopes are able to neutralize the great majority of HIV strains, even at low concentrations [4, 13]. These bnAbs can provide sterilizing immunity against challenge by simian-human immunodeficiency virus (SHIV) in macaques [14, 15], and can reduce viral load to undetectable levels when administered to chronically infected animals [16, 17]. For these reasons, it is thought that an optimally protective vaccine will induce sustained titers of potent bnAbs targeting different epitopes. HIV vaccines have not yet induced bnAbs in humans or animal models, except in one case of a knock-in mouse engineered to express the critical, fully mature heavy chain of a potent HIV bnAb [18].

The plausibility of designing a vaccine that can induce bnAbs is under question in part due to the low frequency and complex mechanisms of bnAb induction in natural infection. Potent bnAbs develop in only a few percent of HIV-infected individuals, typically after two or more years [19, 20], and recent case studies [8, 21, 22] have illustrated how the prolonged and dynamic co-evolution between mutating virus and the adaptive immune system occasionally selects these rare bnAbs from the repertoire [23]. In contrast, a bnAb-based HIV vaccine should induce bnAbs much more reliably—in a majority of vaccine recipients—and should achieve this feat using a small number of immunizations.

The plausibility of a bnAb-based vaccine is further challenged by the fact that potent HIV bnAbs typically have one or more unusual features, such as extensive mutation, long (or short) complementarity-determining region 3 (CDR3) loops, insertions, deletions, additional disulfides and/or sulfated tyrosines [1, 2, 4, 24–28]. Aside from CDR3 lengths, which are normally set in the naïve B cell, these unusual features are generally produced by somatic hypermutation and induced by the rapidly mutating HIV Envelope (Env) during infection. Whether vaccines can be developed to consistently induce highly mutated antibodies with these rare but desirable features is not known.

The unusual features of bnAbs to HIV raise the question of whether one can determine which bnAbs are least unusual and therefore potentially less difficult to induce by vaccination. To date, the various features of bnAbs have not been weighed together on a single quantitative scale in order to measure the degree to which HIV bnAbs are unusual compared to other types of antibodies. Considering that such a scale would assist in ranking and prioritization of HIV bnAb epitopes as targets for vaccine design—with the idea that vaccine efforts should focus on the epitopes targeted by the most "normal" or least "unusual" potent bnAbs—here we developed a computational method, termed the Antibody Features Frequency (AFF) method, to estimate the "features frequency" of any antibody sequence. The method compares the features in a given sequence with those in a large panel of sequences obtained by next-generation sequencing of paired [29] and unpaired (this study) heavy and light chains from human memory B cells from multiple donors. Our AFF analysis of HIV bnAbs not only provides a framework to prioritize epitopes but also motivates the development of minimally mutated bnAbs to serve as potentially more realistic vaccine goals compared to known bnAbs.

Aside from which epitopes to prioritize and which bnAbs to use as guides, the critical question is how to design vaccines to elicit bnAbs. One proposed strategy is B-cell-lineage vaccine design, which seeks to use, as immunogens, HIV Env proteins that parallel the evolution of bnAbs in infected individuals [22, 30]. Such a B-cell-lineage method requires detailed phylogenetic data on both the bnAb lineage and the co-evolving Env lineage from an infected human and may require mapping of multiple bnAb lineages to identify "cooperating" lineages [31]. Thus, its applicability is limited to case studies where such data are available, although the generality of the approach may benefit as more lineages are studied.

VRC01-class bnAbs are attractive vaccine leads owing at least to their potency, breadth and protective capacity [17, 32–36], and to the fact that their in vitro neutralization curves typically saturate at 100% viral neutralization, in contrast to some other bnAb classes (V2/Apex, PGT151, and 10E8) that fail to reach complete neutralization for some isolates, likely influenced by glycan heterogeneity [1, 9, 37]. However, longitudinal Env and antibody phylogenetic data sufficient to consider a B-cell-lineage approach have only recently become available [38, 39], thus alternate vaccine design strategies have been required. To develop candidate immunogens to prime a VRC01-class response, we and others have engineered immunogens with affinity for germline-reverted VRC01-class antibodies [40–43]. We have shown that our most advanced germline-targeting immunogen (eOD-GT8 60mer) can prime VRC01-class responses in VRC01-class heavy-chain knock-in mouse models [18, 44] and can bind VRC01-class human naïve B cell precursors at a frequency of 1 precursor per 400,000 to 2.4 million naïve B cells, corresponding to 15–90 precursors per resting human lymph node [43].

While germline-targeting immunogens are available as candidates to prime a VRC01-class response, the high degree of mutation and other unusual features in VRC01-class bnAbs have made it very difficult to postulate which or how many different boosting immunogens may be required to induce sufficient favorable (bnAb-like) somatic mutations to produce VRC01-class bnAbs via vaccination. Multiple structural and bioinformatic analyses have elegantly defined much of the VRC01-class epitope and paratope [25, 34, 40, 45–49], and one study engineered VRC01 bnAb variants with reduced framework mutations that improved our understanding of which mutations are required for bnAb activity [50]. However, a picture has persisted that there are important mutations scattered across the CDRs and framework regions of VRC01-class bnAbs, and it remains unclear what types of immunogen structures should be employed as boosts to favor elicitation of the most critical mutations.

Here, we developed two minimally mutated VRC01-class bnAbs, one of which had features more common than those in other potent HIV bnAbs analyzed but similar to those in antibodies elicited by common vaccines. We further employed mutational and structural analyses to gain insight into the types of immunogen structures that might be required to induce a minimal set of mutations needed for VRC01-class bnAb activity.

## Results

### Antibody features frequency analysis

**Fig 1:**
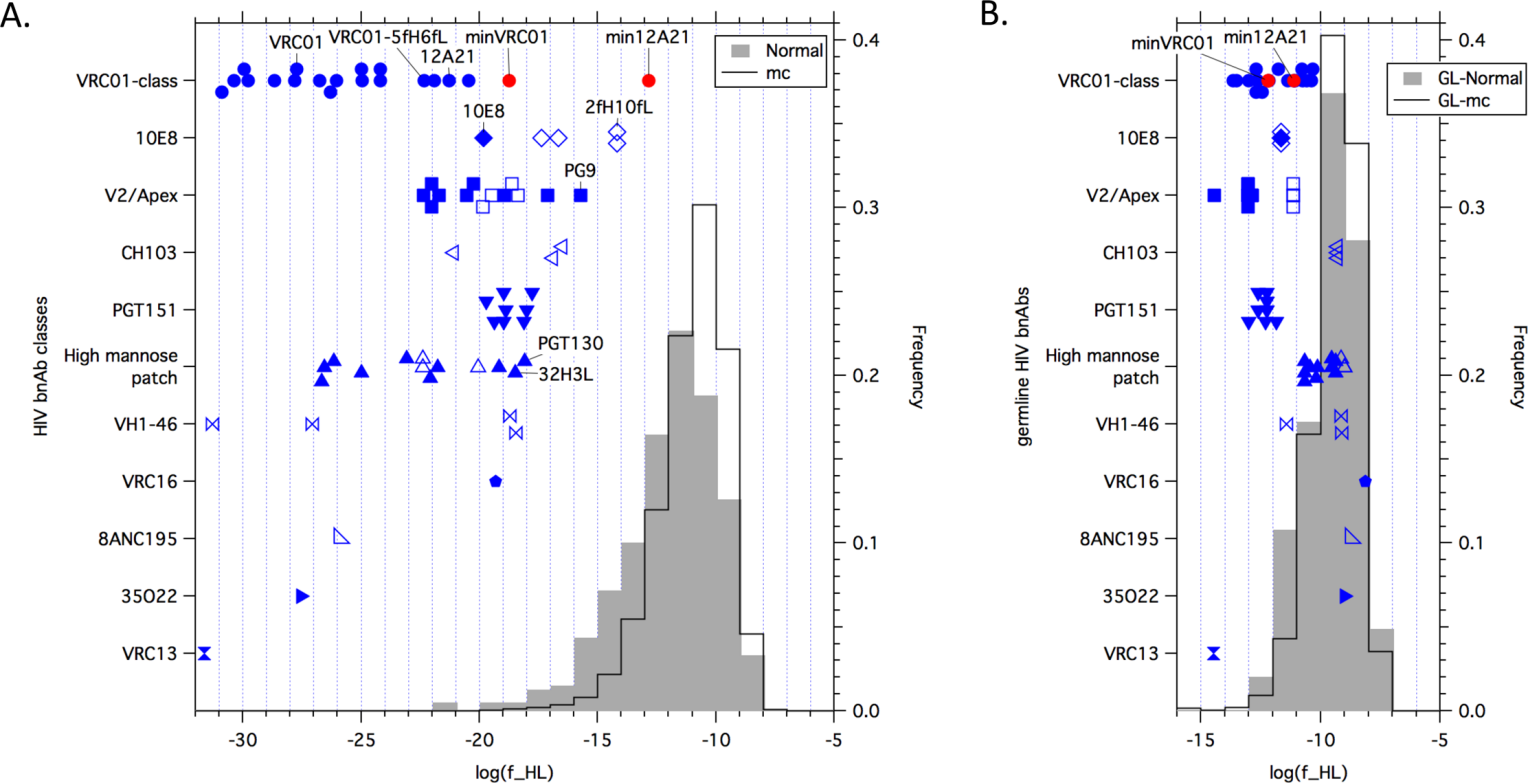
Antibody features frequency analysis. (**A**) Log_10_ antibody features frequencies plotted for HIV bnAbs of different classes (left y-axis), the distribution of Log_10_ antibody features frequencies plotted for a set of 388 "normal" human memory (and plasmablast) antibodies isolated by B cell sorting from human memory B cells [56](and this study), influenza infection [55], HPV vaccination [54](and this study), anthrax vaccination (this study), tetanus toxoid vaccination [52], and HIV RV144 glycoprotein vaccination [51, 53] (gray histogram, right y-axis), and the distribution of Log_10_ antibody features frequencies plotted for a set of 300,000 antibody sequences generated by Monte Carlo ("mc") via the AFF method (black line histogram, right y-axis). Potent HIV bnAbs (mean or median IC_50_ < 0.5 μg/mL [27]) are shown with solid blue symbols, while less potent HIV bnAbs (mean or median 0.5 ≤ IC_50_ < 5.0 μg/mL) are shown with open blue symbols. HIV bnAbs previously engineered with reduced mutations are indicated (VRC01-5fH6fL [50], the 10E8 variant 2fH10fL [50], and the PGT124 variant 32H3L [40]). The shape of the distributions for "normal" and "mc" memory antibodies reflects the smearing of the germline distributions shown in (**B**) to lower features frequencies due to the effects of mutations, insertions and deletions. The slightly increased smearing of the "normal" compared to the "mc" memory distribution stems from the slightly higher mutation frequencies in the "normal" Abs, which are likely due to the fact that all the "normal" Abs except those from Tiller *et al*. [56] were affinity-selected either by antigen-specific B cell sorting [51–54] or by direct affinity measurements on recombinant antibodies after cloning from plasmablast B cells [55]); hence, the "mc" features frequency distribution is probably a better representation of the human memory repertoire. (**B**) Antibody features frequencies for germline versions of the antibodies in (**A**). The shape of the germline distribution curve (for GL-Normal or GL-mc) reflects both the great diversity of the human antibody repertoire and combinatorial statistics. The minimum in the distribution at high features frequency (log(f) = -7) is due to germline antibodies composed of the most common V_H_D_H_J_H_, V_H_V_L_, and V_L_J_L_ combinations and having the most common CDR-H3 and CDR-L3 lengths; such Abs have the highest features frequencies (of ~10^−7^), but there are relatively few such combinations, so they are created infrequently. The peak of the germline distribution at features frequency of ~10^−10^ is due to antibodies that utilize somewhat less frequent but not rare individual components; as there are a very large number of such combinations, these are created frequently. The tail in the distribution at low features frequency is due to germline antibodies composed of the least common V_H_D_H_J_H_, V_H_V_L_, and V_L_J_L_ combinations and the use of rare H-CDR3 and/or L-CDR3 loop lengths. Potent HIV bnAbs with the lowest germline features frequencies either had long H-CDR3 loops (V2/Apex and PGT151) or short L-CDR3 loops combined with less frequent V_L_ or J_H_ chains (VRC13 and some members of the VRC01-class), and all but two HIV bnAbs (CAP256-VRC26.08, with a rare H-CDR3 length of 39, and VRC13) had germline features frequencies greater than 10^−14^.

The AFF method computes the antibody features frequency as the product of the frequencies for the following individual sequence features: the combination of V_H_, D_H_, and J_H_ genes on the heavy (H) chain; the light (L) chain V_L_ given the V_H_; the light chain J_L_ given the V_L_; the lengths of the complementarity-determining-region 3 regions (H-CDR3 and L-CDR3); the percentages of amino-acid mutations on V_H_ and V_L_; the ratios of framework mutations to total V gene mutations on V_H_ and V_L_; the number and size of insertions and deletions on the heavy or light chains as a function of the percent mutation on V_H_ or V_L_, respectively; and the number of cysteines on the heavy or light chains as a function of the percent mutation on V_H_ or V_L_, respectively (see Materials and Methods and S1–S3 Figs). To test the AFF method, we compared the features frequencies for 388 "normal" memory (or plasmablast) antibodies induced by vaccines [51–55] (and this study) or found in the human memory B cell repertoire [56] with the features frequencies for 300,000 antibody sequences computationally generated by the Monte Carlo method (mc) to be consistent with the frequency distributions of the individual features (S1 Fig). The "normal" antibodies, which were not employed in the parameterization of the AFF method, had a mean (± standard deviation) log antibody features frequency (mean log(f)) of -12.0 ± 2.1, while the "mc" antibodies had a mean log(f) of -11.0 ± 1.5. The similarity of these two distributions supports the validity of the method (Fig 1A). Further support came from agreement between the distributions of features frequencies for germline versions of the "normal" and "mc" antibodies, in which germline antibodies were computationally assigned to have no mutations, insertions, or deletions, and to have only two cysteines (that form the conserved internal disulfide bond in Ig domains) in each heavy or light chain variable fragment (Fv) region (Fig 1B). The germline "normal" mean log(f) was -9.6 ± 1.1, while the germline "mc" mean log(f) was -9.4 ± 1.0.

We next evaluated the features frequencies for many HIV bnAbs (Fig 1 and S4 Fig). The set of 49 potent HIV bnAbs (mean or median IC_50_ < 0.5 μg/mL [27] against >50% of diverse viruses in the TZM-bl assay [57] or a similar assay [58]), had a mean log(f) of -23.0 ± 4.1, significantly lower than -12.0 ± 2.1 for "normal" Abs or -11.0 ± 1.5 for "mc" Abs. The set of 19 less potent HIV bnAbs (0.5 μg/mL < mean or median IC_50_ < 5 μg/mL) had a similarly low mean log(f) of -20.0 ± 4.4. Furthermore, all but one potent HIV bnAb (PG9) had features frequencies of less than 10^−17^, whereas only 2.8% (11 of 388) of "normal" antibodies and 0.39% (1179 of 300,000) of "mc" antibodies had such frequencies (Fig 1A). Despite those differences, the features frequencies for germline HIV bnAbs (mean log(f) = -11.4 ± 1.6) were similar to those for germline "normal" or "mc" antibodies (Fig 1B). Thus, the significantly lower features frequencies of HIV bnAbs compared to normal human memory antibodies are largely due to higher levels of mutations, insertions and deletions accumulated during affinity maturation, with a smaller contribution from unusual germline features. Among the known potent bnAbs, the least unusual that fall within the tail of normalcy in Fig 1 are the V2/Apex bnAbs PG9 and CAP256, followed by several PGT151-class bnAbs and the high mannose patch bnAbs PGT130 and 32H3L (Fig 1 and S4 Fig). Overall, this analysis quantifies the large (several orders of magnitude) gap in features frequencies between HIV bnAbs and normal human memory antibodies; this gap indicates that the known potent HIV bnAbs generally provide poor direct leads to guide HIV vaccine development, because antibodies with similar features are unlikely to be elicitable in a consistent manner. In contrast, a set of eight anti-influenza bnAbs had a mean log(f) of -11.9 ± 1.6, with values ranging from -14.4 to -10.6, consistent with typical human memory antibodies (S5 Fig). Moreover, the analysis suggests that engineering or discovery of potent HIV bnAbs with higher features frequencies will be needed to focus vaccine efforts toward epitopes targeted by more plausibly inducible potent bnAbs.

### Development of minimally mutated VRC01-class bnAbs

Our AFF analysis showed VRC01-class bnAbs to be among the most unusual of all HIV bnAbs, with log features frequencies ranging from -21.3 to -30.9. Even VRC01-5fH6fL, a VRC01-derived bnAb previously engineered to have reduced framework mutation [50], retained a low log features frequency of -22.3 (Fig 1A), due to substantial mutation levels in V_H_ (19.8%) and V_L_ (15.1%), and to an insertion and a deletion on the light chain (S1B Fig and ref. [50]).

**Fig 2:**
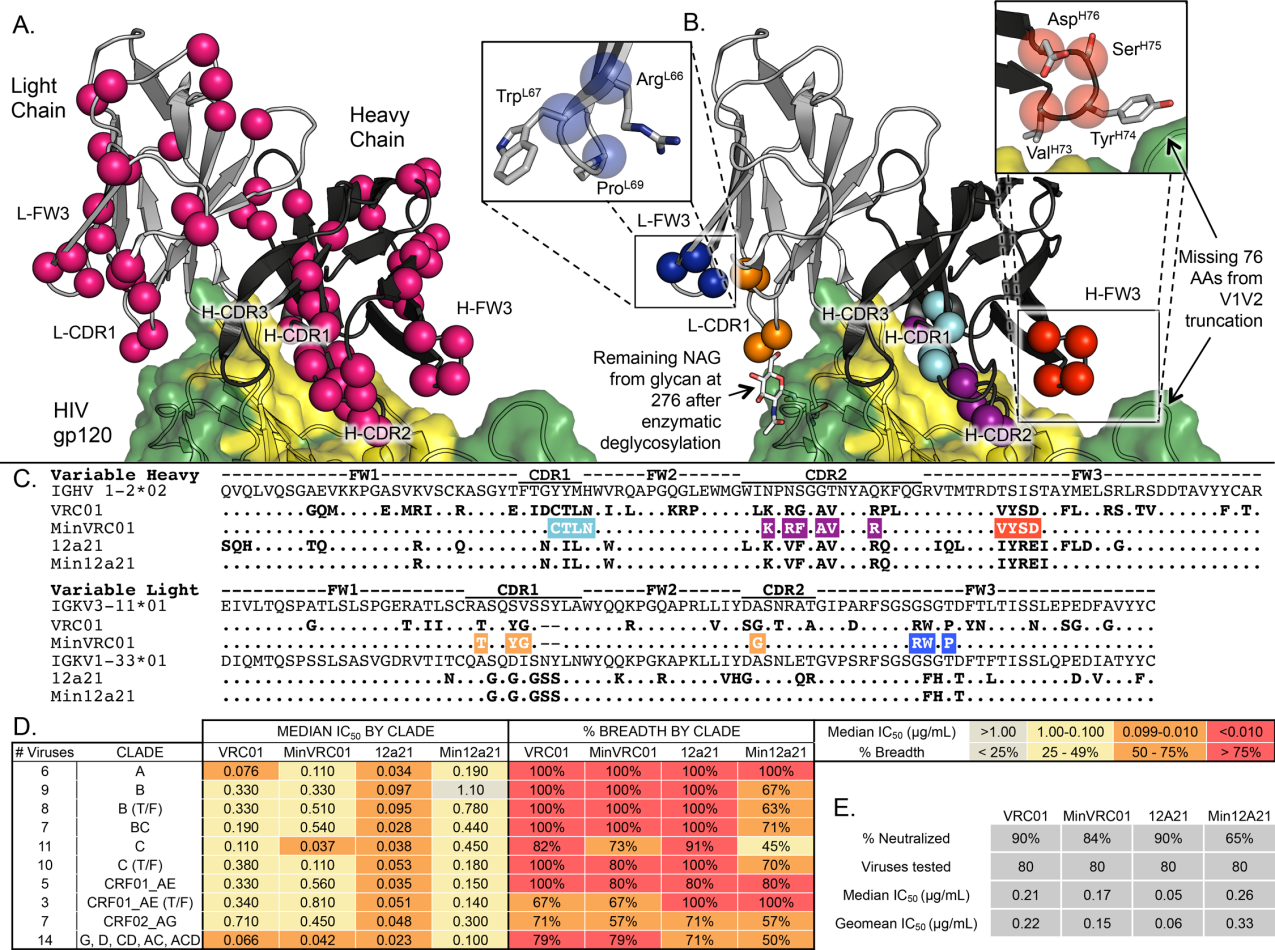
Limited somatic hypermutation of VRC01-like antibodies is sufficient to afford broad and potent neutralization. (**A**) The crystal structure of VRC01 (heavy chain in black, light chain in gray) in complex with core gp120 (PDB ID 3NGB, green surface with CD4bs highlighted in yellow) with mutations from the inferred germline variable genes highlighted as pink spheres. (**B**) The same structure as in (**A**) showing only the mutations retained in MinVRC01 following directed evolution. Mutations are grouped together into patches distinguished by color. (**C**) Alignment of VRC01 and MinVRC01, 12A21, and Min12A21 with their germline variable genes, highlighting mutations from germline for MinVRC01. Mutations are colored according to scheme in (**B**). (**D**) Neutralization of a 80-virus cross-clade panel by VRC01, MinVRC01, 12A21, and Min12A21. Values for median neutralization IC_50_ (μg/mL) and percent breadth are displayed by clade and colored according to the legend. (**E**) Summary of neutralization breadth and potency of VRC01, MinVRC01, 12A21 and Min12A21.

To establish improved plausibility for a bnAb-based HIV vaccine, we developed two minimally mutated VRC01-class bnAbs. Though VRC01-class bnAbs are among the most mutated of HIV bnAbs [2, 3, 59], crystal structures of VRC01-class bnAbs bound to various core gp120s revealed that many of the somatic mutations are distally located from the core epitope, suggesting that some mutations may not be necessary for potent and broad virus neutralization (Fig 2A and refs. [25, 50]). We developed a yeast surface display method to assess the importance of all V_H_ or V_L_ mutations for broad gp120 recognition. Libraries based on an inferred germline VRC01 were generated in which positions mutated in VRC01 V_H_ and V_L_ genes sampled only the germline and mutated residues; the libraries also sampled reversion of the insertion and deletion on the VRC01 light chain. These libraries were then sorted for high-affinity binding to a set of recombinant gp120 proteins from diverse HIV strains. Mutations that were heavily enriched (67% threshold) were retained.

Using this strategy, we developed two minimally mutated VRC01-class bnAbs, MinVRC01 and Min12A21, with excellent neutralization breadth and potency only slightly or moderately diminished compared to the original bnAbs VRC01 [2] and 12A21 [3] on a cross-clade 80-virus panel (Fig 2B to 2D and S1 Table). The V_H_ genes of MinVRC01 and Min12A21 are 13% and 17% mutated from the germline V_H_1-2*02 precursor at the amino-acid level, respectively, whereas those of VRC01 and 12A21 are 42% and 32% mutated, respectively, showing that ~1/2 to 2/3 of the mutations in VRC01 and 12A21 are not strictly required for neutralization. Both MinVRC01 and Min12A21 retain a short L-CDR3 loop of five residues that appears to be structurally required for VRC01-class bnAbs to bind their gp120 epitope [46, 49]. MinVRC01, but not Min12A21, retains a deletion in L-CDR1 and a disulfide between H-CDR1 and H-CDR3, both of which are present in some VRC01-class bnAbs. MinVRC01, but not Min12A21, contains a single mutation that arose from PCR error, G54F, that had previously been shown to improve the potency of VRC01-class antibodies [60]. We retained this F54 in MinVRC01 because it is important for the breadth of MinVRC01, and similar residues (F,Y,W) are found at this position in multiple other VRC01-class bnAbs (12A12, 12A21, VRC03, VRC-PG20). Overall, MinVRC01 and Min12A21 have higher features frequencies than any other VRC01-class bnAb, and Min12A21 has the highest features frequency of all HIV bnAbs examined in this study (Fig 1A). The development of these minimally mutated bnAbs, particularly Min12A21, establishes a more feasible goal for reliable vaccine elicitation of potent anti-HIV bnAbs.

### Polyreactivity analysis

**Fig 3:**
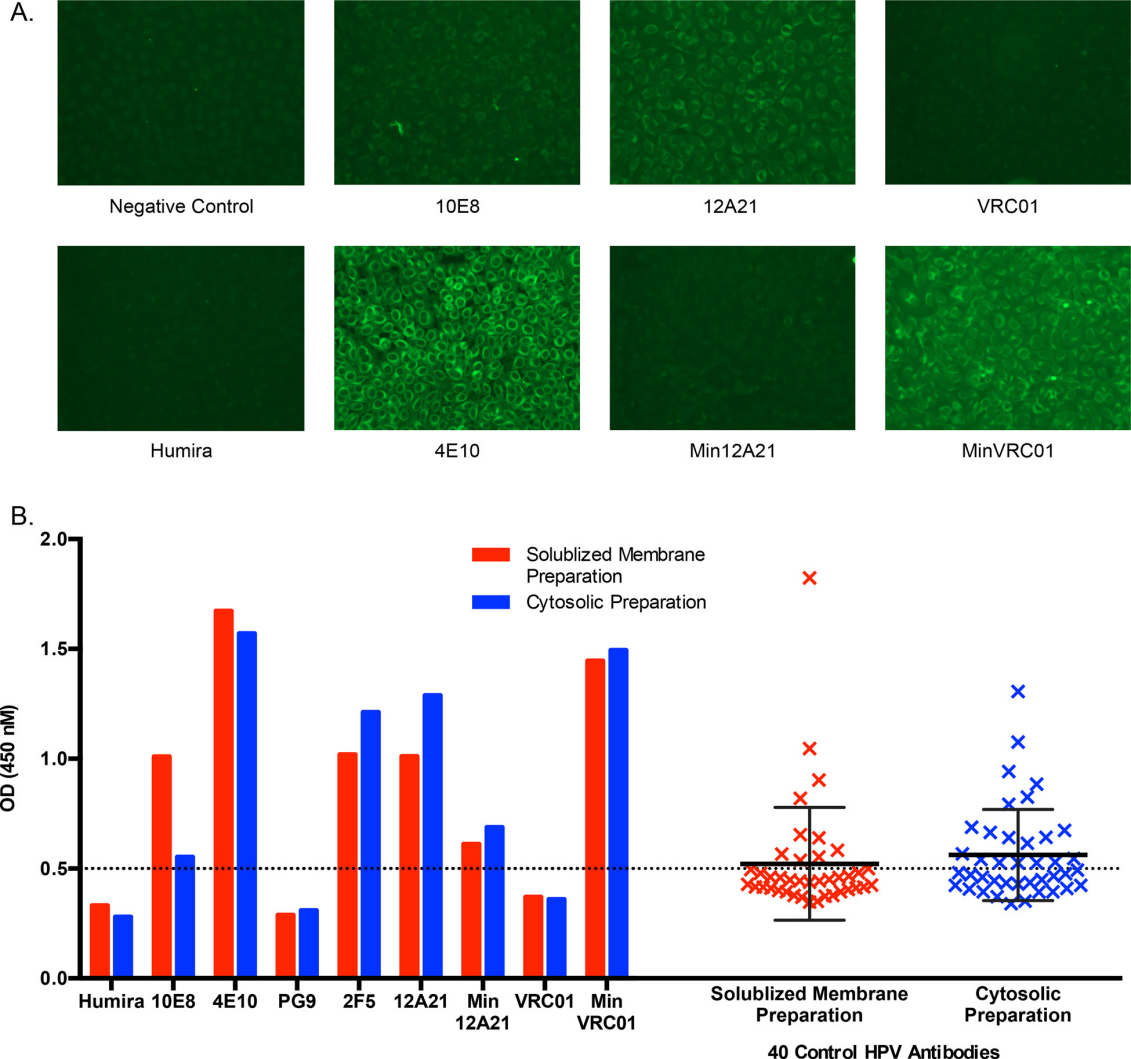
Polyspecificity analysis of MinVRC01 and Min12A21. (**A**) HEp-2 cell staining assay, for which 4E10 is a positive control while Humira, 10E8, and undiluted human serum "Negative control" are negative controls. (**B**) Polyspecificity reagent (PSR) binding assay measuring binding to preparations of solubilized membrane proteins and cytosolic proteins from CHO cells. Here, 4E10 and 2F5 are positive controls, while Humira, 10E8, PG9 and 40 HPV-vaccine induced human mAbs are negative controls. Error bars reflect mean ± standard deviation with N = 40.

Many HIV bnAbs have been identified as poly- or auto-reactive; thus, tolerance mechanisms may serve as a barrier to elicitation of some bnAbs [27]. To assess the polyreactivity of MinVRC01 and Min12A21, we conducted four assays: cardiolipin binding, HEp-2 cell staining, single autoantigen reactivity, and a polyspecificity reagent (PSR) binding assay [61, 62] measuring binding to preparations of solubilized membrane proteins or cytosolic proteins (Fig 3 and S6 Fig). By all four assays, 12A21 appeared clearly polyreactive whereas Min12A21 did not. In contrast, MinVRC01 was polyreactive in all four assays, whereas VRC01 showed no evidence of polyreactivity. As noted above, MinVRC01 contains one mutation, G54F, that is absent from VRC01. The point mutant of MinVRC01 containing the VRC01-germline serine at position 54 (MinVRC01-F54S) retained polyreactivity (though reduced compared to MinVRC01), and the Phe54-variant of VRC01 (VRC01-G54F) lacked polyreactivity (S6 Fig), therefore Phe54 is not the sole source of polyreactivity in MinVRC01. Thus, while the reduction of mutation from 12A21 to Min12A21 removed polyreactivity, the removal of mutations from VRC01 to create MinVRC01 gave rise to polyreactivity. Overall, these results further support Min12A21 as a more realistic target for vaccine elicitation.

### Hierarchy of mutation patches for neutralization potency and breadth

**Fig 4:**
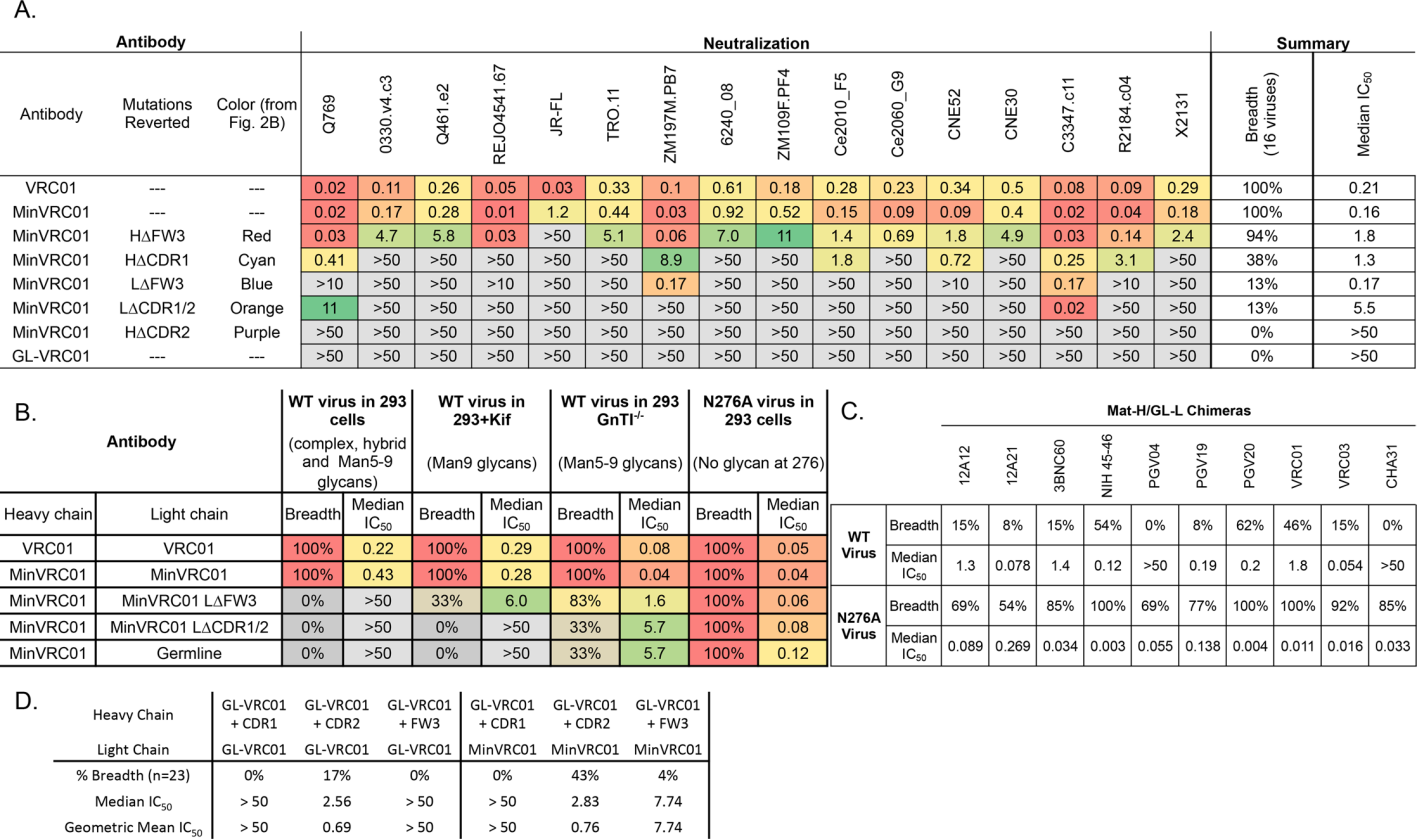
Reversions of MinVRC01 patches to germline reveal importance of affinity maturation in the light chain to accommodate the N276 glycan on Env. (**A**) Neutralization on a 16-pseudovirus cross-clade panel was performed for each reverted antibody variant. Neutralization values are presented in μg/mL and colored by most potent (red) to least potent (green). (**B**) Neutralization of MinVRC01 light chain reversions against a 6-pseudovirus panel of wild-type (WT) virus, viruses produced with kifunensine, which yields virions with Man_9_GlcNAc_2_ glycans on Env, viruses produced in GnTI^-/-^ cells, which yields virions with Man_5-9_GlcNAc_2_ glycans on Env, and viruses with the glycan site at N276 on Env removed by alanine substitution (N276A). (**C**) VRC01-class bnAbs were produced as mature heavy chain and germline light chain chimeras and tested on a 13-pseudovirus panel with (wild-type, WT) and without (N276A) the glycan site at position 276 of Env. Values are percent breadth and median neutralization IC_50_ (μg/mL); all measurements were performed in duplicate. (D) Neutralization of N276A viruses by variants of GL-VRC01 and GL-VRC01-HC/MinVRC01-LC in which either the H-CDR1, H-CDR2 or H-FW3 mutation patch was restored. All glycan-modified viruses were tested against non-neutralizing and weakly neutralizing antibodies b6, b12 and F105, as well as CD4-IgG2 (S6 Table). These agents showed no neutralization or no increase of neutralization over WT virus, confirming that the N276 interaction is specific to the VRC01 light chain and does not cause a global increase in neutralization sensitivity.

Mutations in MinVRC01 and Min12A21 could be grouped into five spatial patches, three on V_H_ and two on V_L_ (Fig 2B and 2C, colored patches). Alignments of VRC01-class heavy or light chains (S7 Fig) revealed similar patches in many of these bnAbs. To assess the relative importance of the mutation patches for antibody function, we generated variants of MinVRC01 with individual patches reverted to germline and tested these patch-revertants for neutralization on a cross-clade 16-pseudovirus panel (Fig 4A and S2 Table). The data revealed a hierarchy of importance for neutralization potency and breadth for the patches, with H-CDR2 > L-CDR1/2 ≈ L-FW3 > H-CDR1 > H-FW3. Notably, while the H-ΔFW3 revertant retained high breadth (94%) and modest potency (1.8 μg/mL) and the H-ΔCDR1 revertant showed modest breadth (38%) and potency (1.3 μg/mL), the H-ΔCDR2 revertant failed to neutralize any viruses and the L-ΔCDR1/2 and L-ΔFW3 revertants each neutralized only 2/16 viruses in the panel. We generated the same patch reversions on VRC01 and observed similar effects but diminished in magnitude, suggesting the parent bnAb may contain some redundancy (S2 Table).

### Structural analyses to clarify patch interactions

Previous structural analyses of VRC01, 12A21 and other VRC01-class bnAbs interacting with Env subunits provide considerable information on the structural roles of the residues within the patches. The H-CDR2 patch makes direct contacts with four gp120 elements (loop D, β15/α3, β20/21 and β23/V5) in all VRC01-class bnAbs [25]. The H-CDR1 patch makes little direct gp120 contact [25, 49, 60], but owing to its importance for neutralization we hypothesize that it may stabilize the adjacent H-CDR2. H-FW3 contacts residues from β3 and β20/21 on the V1/V2 truncated gp120 core [25, 49, 60] and has been proposed to contact V3 residues on a neighboring protomer in the trimer [48], but the precise role of H-FW3 has remained unclear. L-CDR1 in all VRC01-class bnAbs includes either deletions or mutations to glycine, which has been interpreted as a requirement to avoid a clash with Loop D on gp120 [25, 49]. Comparative analysis of light chains in VRC01 and NIH45-46, a VRC01-class bnAb with 89% V_H_ sequence identity to VRC01 [33], and structural analysis of 45-46m2, an engineered variant of NIH45-46 with improved breadth and potency, have indicated an important but not obligatory role of Tyr28 in VRC01 L-CDR1 for contacting the N276 glycan, and have also shown that 45-46m2 L-CDR2 and L-FW3 residues can contact a short Man_4_GlcNAc_2_ glycan at position 276 [45, 47].

**Fig 5:**
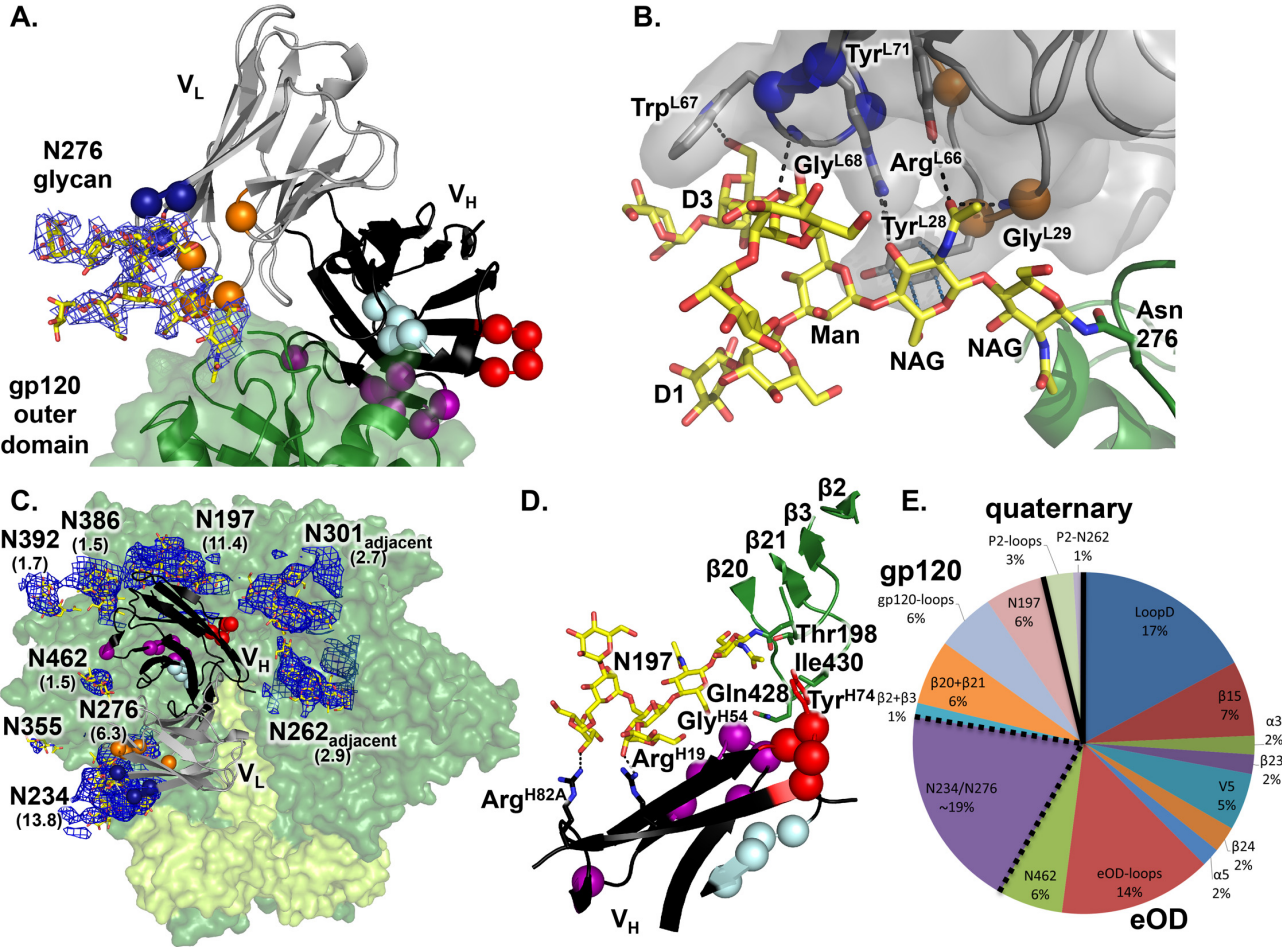
Structural definition of the full VRC01-class bnAb epitope in the context of the Env trimer. (**A**) Crystal structure of eOD-N276Kif containing a Man_9_GlcNAc_2_ glycan at N276 in complex with VRC01, with the critical MinVRC01 mutations from germline shown as spheres and colored as in Fig 2B. A 2mFo-DFc simulated annealing composite omit map displayed at a contour level of 1.0 σ for the N276 glycan is shown in a blue mesh. (**B**) Close-up view of the interactions of the N276 glycan with the light chain. Mutations identified in MinVRC01 are highlighted as spheres and the side chains are shown as sticks. (**C**) Crystal structure of NIH45-46 scFv in complex with the BG505 SOSIP Env trimer (gp120, green surface; gp41, yellow surface) and PGT122 Fab (omitted for clarity). A blue mesh 2mFo-DFc composite omit map is displayed at a contour level of 1.2 σ for glycans that are part of or surround the CD4bs epitope. Numbers in parentheses below each glycan name indicate the fold decrease in VRC01 IC_50_ (i.e. increase in potency) upon substituting that glycan with an alanine. (**D**) Close-up view of the interaction between the NIH45-46 H-CDR2 and H-FW3 patches with the gp120 bridging sheet (green) in a pre-fusion, pre-CD4 bound conformation and with the N197 glycan. (**E**) Buried surface area analysis of VRC01-class bnAbs on eOD, gp120 and partially deglycosylated BG505 SOSIP reveal that the majority of the epitope components are contained on eOD, but important components are also present on the inner domain and bridging sheet of gp120 on the same protomer (gp120) as well as loops and a glycan from the adjacent protomer (quaternary). P2 refers to the adjacent protomer. The trimer restricts the antibody angle of approach to the CD4bs due to quaternary packing constraints and glycan fencing.

To further dissect the role of light chain patches in interacting with the N276 glycan, we determined the crystal structure at 3.25 Å resolution of Fab VRC01 bound to an engineered gp120 outer domain (eOD) that contains a Man_9_GlcNAc_2_ glycan at position 276 (eOD-N276Kif) (Fig 5A). The VRC01 interactions with the N276 glycan contribute an additional 550Å^2^ of buried surface area to the previously defined epitope [25], and an additional 229 Å^2^ of buried surface area compared to the 45-46m2+gp120 structure with a shorter Man_4_GlcNAc_2_ glycan at position 276 [47], due to a significantly different glycan conformation, several new H-bonds, and increased stacking interactions with Tyr^L28^ (Fig 5B). All three L-FW3 affinity-matured residues (Arg^L66^, Trp^L67^ and Pro^L69^) and two of the L-CDR1 affinity-matured residues (Tyr^L28^ and Gly^L29^) directly contact the N276 glycan (Fig 5B and S8 Fig). Together, the Man_9_GlcNAc_2_ N276 glycan interactions constitute a third of the antibody paratope surface area. The two-residue deletion in L-CDR1 eliminates a steric clash between the germline-VRC01 L-CDR1 and the GlcNAc_2_ base of the N276 glycan (S9A Fig). Steric clashes between germline VRC01 L-FW3 and the N276 Man_9_GlcNAc_2_ D2 arm are alleviated by mutations in mature VRC01 (S9B Fig). Structural comparisons with other VRC01-class bnAbs co-crystallized with core gp120 suggest that all VRC01-class antibodies have evolved similar mutations to avoid and/or utilize the N276 glycan (S9C and S9D Fig). The glycoforms present at N276 on infectious particles remain to be identified, information that may be important for VRC01-class vaccine development. Fully-solvated molecular dynamics simulations of eOD with a complex biantennary glycan at N276 suggest that mammalian glycoforms other than oligomannose species could also be accommodated by VRC01-class bnAbs (S10 Fig). This structure and these analyses thus improve our understanding of VRC01-class interactions with the N276 glycan.

To further delineate the full extent of the VRC01-class epitope and paratope, we determined the crystal structure (at 4.4 Å) of a partially deglycosylated BG505 SOSIP trimer in complex with PGT122 Fab and the single-chain variable fragment (scFv) of NIH45-46. The trimer backbone conformation was very similar to the same trimer crystallized either with PGT122 alone [63] (Cα RMSD 0.97 Å for gp120) or in the unliganded state [64] (Cα RMSD 0.74 Å for gp120), illustrating that NIH45-46 binds to the "ground-state" conformation of Env [65] and does not induce Env conformational changes.

The VRC01-class epitope is densely surrounded by N-linked glycans (Fig 5C), as suggested in the cryo-EM structure of the BG505 trimer with VRC01-class bnAb PGV04 [48]. Glycans account for at least 30% of the surface area buried by NIH45-46 on the trimer; the antibody interacts with N197, N234, N276 and N462 glycans on one gp120 protomer and with the N262 glycan on the adjacent gp120 protomer (S3 Table). The N276 glycan appears to be truncated in the trimer structure, as there is no density for glycan moieties beyond the first NAG, which would be consistent with trimming by EndoH that was employed for deglycosylation of the protein used for structural study. This considerably reduces the buried surface area on the 276 glycan compared to the eOD-VRC01 structure. In the absence of the N276 glycan, the N234 glycan appears to have avoided trimming and makes extensive contacts to the NIH45-46 L-FW3 patch, effectively replacing the N276 glycan interactions with L-FW3 seen in the eOD+VRC01 complex. This indicates promiscuity in the ability of the L-FW3 to interact with glycans, and also a level of redundancy in antibody recognition of the HIV Env glycan shield [66].

Neutralization of pseudoviruses with glycan knock-out mutations demonstrated that VRC01 and 12A12 potency improved when the N197, N234, N262, N276 or N462 glycans were absent (Fig 5C and S4 Table). We therefore conclude that N-linked glycans surrounding the CD4bs on the trimer restrict antibody angles of approach and reduce binding and neutralization by VRC01-class bnAbs. The MinVRC01 mutations that enable N276 glycan interactions are required for neutralization of viruses bearing that glycan (see below). Thus, while the HIV Env trimer has evolved a glycan fence around the CD4 binding site that imposes steric and entropic penalties to antibody binding [67, 68], VRC01-class bnAbs appear to have evolved solutions that avoid glycan clashes and partially offset the entropic penalties.

Based on our trimer structure, we estimate that NIH45-46 buries ~25% more area on the Env trimer than on core gp120, nearly half of which is due to additional protein contacts (S3 Table). Approximately 75% of the area buried by NIH45-46 on the trimer is contained within elements present on eOD, ~20% is within the bridging sheet or inner domain of the same protomer, and ~5% comes from an adjacent protomer (Fig 5E and S3 Table). Importantly, NIH45-46 recognizes the bridging sheet in its pre-fusion conformation on the BG505 SOSIP trimer, positioning the H-FW3 patch to interact with gp120 elements β21, β3 and the base of the V2 loop (Fig 5D and 5E and S3 Table). In contrast, when NIH45-46 and other VRC01-class bnAbs bind core gp120, the bridging sheet adopts the CD4-bound conformation, resulting in a considerably different interaction surface for H-FW3. Moreover, the N197 glycan, at the V2 base on the trimer, is in H-bonding distance to two affinity-matured NIH45-46 residues, namely Arg^H19^ (H-FW1) and Arg^H82A^ (H-FW3). Arg or Lys is present in most VRC01-class bnAbs at these positions, suggesting that these residues may make important contributions to neutralization potency and/or breadth. The trimer structure also indicates that the NIH45-46 H-FW3 patch is probably too distant from the adjacent V3 region to make extensive interactions, although other bnAbs with H-FW3 insertions, such as 3BNC60, likely have evolved to do so more effectively [26]. Overall, the trimer structure reveals the importance of employing native-like trimer immunogens in regimens to induce VRC01-class bnAbs—only native-like trimers provide the pre-fusion conformation of the bridging sheet, the full VRC01-class epitope including both protein and glycan contacts, and the restrictions on angle of approach due to quaternary packing and the glycan fence around the CD4bs that are also present on the virus.

### Effect of light chain patches on neutralization of viruses with different glycoforms

We next evaluated how the different gp120 glycoforms modulated neutralization by MinVRC01 light chain revertants (Fig 4B). Neutralization was tested against a 6-pseudovirus panel produced in four conditions: (i) in 293T cells, resulting in viruses having diverse biologically relevant glycoforms, including complex, hybrid and oligomannose glycans, (ii) in 293T cells grown with kifunensine (293kif), producing viruses with uniform Man_9_GlcNAc_2_ glycans, (iii) in 293 GnTI^-/-^ cells, yielding viruses with oligomannose glycans, (Man_5_GlcNAc_2_ to Man_9_GlcNAc_2_) [69]_,_ and (iv) in 293T cells with an Env point mutation to eliminate the 276 glycosylation site (N276A). While both L-CDR1/2 and L-FW3 mutations are required to achieve maximum breadth and potency against wild-type viruses, the L-CDR1/2 mutations alone are necessary and sufficient for modest neutralization of GnTI^-/-^ viruses, consistent with the proximity of the L-CDR1/2 mutations to the base of the N276 glycan (Fig 5 and S9 Fig). Furthermore, the light-chain germline revertant broadly neutralizes a panel of N276A viruses, which suggests that the primary function of the light-chain somatic mutations of MinVRC01 is to accommodate the N276 glycan. This conclusion holds for VRC01-class bnAbs in general, based on our neutralization assays for mature-H/germline-L chimeras of 10 different VRC01-class bnAbs against a panel of 13 pseudoviruses with and without N276A. All 10 chimeras are potent and broad neutralizers against the N276A panel, with breadth >50% and median IC_50_ <0.3 μ/mL, but only 2 of 10 show similar activity against the panel with N276 intact (Fig 4C and S5 Table), consistent with our structural analysis (Fig 5).

### Minimal heavy chain mutations

Following on from the finding that MinVRC01-HC/GL-VRC01-LC and other VRC01-class mature-H/germline-L chimeras broadly neutralize N276A viruses, and with the concomitant realization that neutralization of N276A viruses could be an important readout on the pathway to induction of VRC01-class bnAbs, we sought to determine more precisely the minimal heavy-chain mutations required for broad neutralization of sensitive N276A viruses. We generated variants of GL-VRC01 and GL-VRC01-HC/MinVRC01-LC in which we restored only the H-CDR1, H-CDR2 or H-FW3 patches, and we tested these Abs against a panel of 23 N276A-sensitive viruses. These experiments showed that, for many viruses, only the six H-CDR2 mutations were necessary for weak neutralization (Fig 4D). The GL-VRC01+H-CDR2 HC paired with the GL-VRC01 LC variant had 17% breadth across diverse viruses, while the same heavy chain paired with the MinVRC01 LC had 43% breadth against the same panel. Both Abs neutralized at least one virus each from clade A, B and C.

## Discussion

We and others have found that removal of the N276 glycan and other glycans surrounding the VRC01-class epitope is necessary to develop candidate priming immunogens with appreciable affinity for germline-reverted VRC01-class Abs [40, 41]. We previously proposed that ultimate elicitation of VRC01-class bnAbs will likely require boosting with one or more immunogens bearing the N276 glycan and other highly conserved glycans near the epitope [40, 41, 44], otherwise the elicited antibodies may not tolerate the presence of those glycans and hence may fail to neutralize most tier 2 viruses. That logic provides a backdrop for the present work.

Our finding here that VRC01-class mature-H/germline-L chimeras broadly neutralize N276A viruses, combined with previous demonstrations that VRC01-class germline B cell activation requires the absence of the N276 glycan [40, 41, 44, 70], suggests that vaccine regimens employing immunogens lacking the N276 glycan could be developed to induce the MinVRC01 heavy chain mutations or their equivalents and thereby achieve an intermediate goal of inducing bnAbs against N276A viruses. That MinVRC01 L-ΔFW3 broadly neutralizes GnTI^-/-^ viruses further suggests that vaccine selection of light-chain mutations could be achieved incrementally, by first employing immunogens produced in GnTI^-/-^ or insect cells (to select the L-CDR1/2 mutations required to accommodate small oligomannose glycans without simultaneously pressuring L-FW3), and next employing immunogens produced in 293T cells (to select the L-FW3 mutations required to accommodate larger native glycans). From our analysis of minimal heavy-chain mutations required for neutralization of N276A viruses, we conclude that very few (a maximum of six) mutations are needed for VRC01-class cross-neutralizing activity to develop against N276A viruses. This finding offers an early detection signal for the development of VRC01-class bnAbs. Moreover, these results suggest an alternate scenario to one in which the induction of potent autologous neutralization is seen as a necessary step toward induction of heterologous neutralization [71]. For VRC01-class bnAbs, our data suggest that the successful vaccine elicitation of such Abs may be accompanied initially by the emergence of cross-neutralizing activity and subsequently by increases in neutralization potency and breadth.

**Fig 6:**
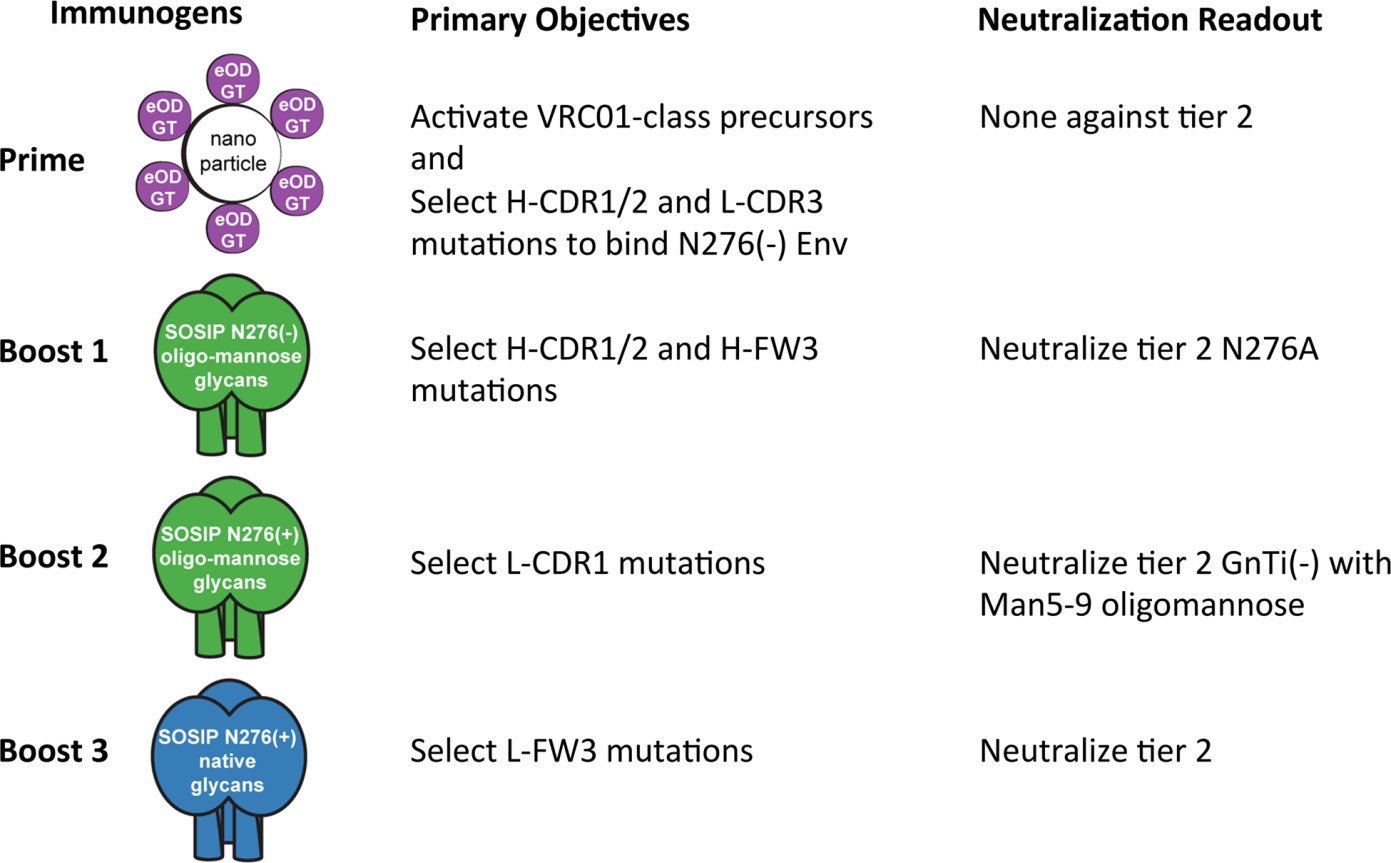
A proposed reductionist vaccine strategy to induce VRC01-class bnAbs. This working-concept strategy has four main objectives for achieving affinity maturation by using a sequence of at least four types of immunogens. Incremental, step-wise progress can be assessed by using neutralization assays against panels of mutant viruses, as well as analysis of antibody sequences from antigen-specific B cells. Thus, using this strategy, each step can be optimized individually. One strategy is indicated; variant strategies, for example incorporating cocktails of Env, can be readily envisaged. "SOSIP" is one type of native-like trimer but other types of native-like trimers could be used.

Based on our findings here and on the development of VRC01-class germline-targeting immunogens [40, 41], we suggest a stepwise vaccine strategy to induce VRC01-class bnAbs, with objectives for affinity maturation at each step (Fig 6). This strategy has four basic objectives, to be achieved by a sequence of four types of immunogens: (i) activation of VRC01-class germline precursors with VH1-2 and short L-CDR3 loops, and concomitant selection of initial H-CDR1/2 and L-CDR3 mutations sufficient for low-affinity recognition of N276A Env, using germline-targeting nanoparticles; (ii) selection of H-CDR1/2 and H-FW3 mutations to allow neutralization of N276A viruses, preferably using native-like N276(-) trimers but possibly requiring a bridging immunogen to focus pressure first on H-CDR1/2 alone; (iii) selection of mutations and/or deletions in L-CDR1 to accommodate the base of the N276 glycan and to allow neutralization of viruses with oligomannose glycans, using native-like N276(+) trimers produced in GnTI^-/-^ or insect cells; (iv) selection of L-FW3 mutations to accommodate the distal portions of the N276 glycan and to allow neutralization of viruses bearing native glycans.

Previous immunization results in the VRC01 gH and 3BNC60 gH knock-in mice [18, 44] support the concept of germline-targeting to initiate the induction of VRC01-class bnAbs, and the 3BNC60 mH mouse results [18] support the notion of using native-like trimers as the final immunogen(s) in the sequence. Our proposed strategy provides a structure-based and data-driven hypothesis for what types of immunogens should come between the germline-targeting prime and the final native-like trimer boost. The proposed strategy should be regarded as a working concept that will likely require refinement and optimization via experimental testing. Additional issues that will need to be addressed include maintaining conserved T help among the different immunogens in the sequence and minimizing the boosting of non-VRC01-class responses that might compete directly or indirectly with the maturation of the VRC01-class response.

### Conclusions

In summary, we have developed two minimally mutated VRC01-class bnAbs and demonstrated that their features frequencies are more concordant with vaccine-induced Abs in general as compared to VRC01-class bnAbs isolated from individuals following many years of HIV infection. Min12A21, with the highest features frequency of any of the 68 bnAbs tested, was also free of polyreactivity. Taking advantage of their reduced mutation, we dissected the structural requirements for broad and potent neutralization by the minimally mutated Abs and then used the resulting knowledge to develop a stepwise vaccination strategy as a working concept that is intended to elicit the necessary antibody features for potent and broad neutralization of HIV viruses. We recognize that any immunogen may induce some level of non-critical mutations. The immunization strategy poses specific objectives for affinity maturation at each step and, as such, is amenable to experimental testing and stepwise optimization in a way that vaccine strategies normally are not. Taken as a whole, this work advances the concept of "reductionist" vaccine design guided by structural analysis of minimally mutated bnAbs and their interaction with HIV Env.

## Materials and Methods

### Next-generation antibody sequencing: RNA isolation from IgG_+_ memory B cells

Peripheral blood mononuclear cells (PBMCs) were isolated from the whole blood of eight healthy donors by gradient centrifugation (Histopaque-1077; Sigma-Aldrich). From each donor, IgG^+^ memory B cells were separated from 10 million PBMCs by selective depletion (Switched memory B cell isolation kit; Miltenyi Biotec) and total RNA was extracted (RNeasy; Qiagen).

### Next-generation antibody sequencing: Library preparation and sequencing

Approximately 10% of each total RNA sample was subjected to reverse transcription (Superscript III; Life Technologies) using cDNA barcoding primers that contain 20 nucleotide long unique antibody identifiers (UAIDs). The resulting cDNA was purified (Qiaquick; Qiagen) and eluted into 50uL of water. 10uL of cDNA was used to amplify antibody heavy chains (HotStarTaq Plus; Qiagen) in a 50uL total reaction volume using the following thermal cycling program: 94°C for 5 min; 30 cycles of 94°C for 30 s, 55°C for 30 s, 72°C for 2 min; 72°C for 7 min. Following initial amplification, PCR products were purified using 45 μL of SPRIselect beads (Beckman-Coulter Genomics) per 50 μL PCR reaction and eluted in 50 μL of water. Illumina sequencing adapters and sample-specific indexes were added during a second round of PCR using 1 μL of purified PCR product in 100 μL of total PCR reaction volume and using the following thermal cycling program: 94°C for 5 min; 10 cycles of 94°C for 30 s, 55°C for 30 s, 72°C for 2 min; 72°C for 7 min. Indexed PCR products were purified using 75 μL of SPRIselect beads and eluted in 50 μL of water. Samples were quantified using fluorometry (Qubit; Life Technologies), pooled at approximately equimolar concentrations and the sample pool was re-quantified. Samples were loaded onto an Illumina MiSeq sequencer with a target loading concentration of 40 pM and 10% PhiX and sequenced (MiSeq 600-base v3 reagent kit; Illumina).

### Next-generation antibody sequencing: Initial sequence analysis and UAID processing

Paired-end MiSeq reads were merged with PANDAseq using default settings [72]. Sequences were annotated with AbStar, an antibody analysis software package based on BLASTn, using human germline V(D)J databases from IMGT [73]. Following annotation, sequences were loaded into a MongoDB database for querying and additional analysis. To correct sequencing and amplification errors, antibody sequences were binned by the cDNA barcode, and all bins containing only a single sequence were discarded. For each bin containing two or more sequences, the appropriate germline variable gene region was added to the bin to serve as a consensus tiebreaker. The bins were then separately aligned with Muscle, and consensus sequences were generated using Biopython. Consensus sequences were re-processed with AbStar and stored in a separate MongoDB database. Following error correction, sequences were screened with two additional quality filters: 1) the sequence must be at least 200 bp (light chains) or 250 bp (heavy chains) in length; and 2) the junction must begin with a conserved cysteine and end with either a phenylalanine (light chains) or tryptophan (heavy chains) and contain no ambiguous codons.

### Processing of DeKosky/Georgiou paired antibody sequences

Raw sequence data from DeKosky *et al*. [29] were downloaded from the Short Read Archive (SRA) and, because SRA files are generated by concatenating paired Illumina reads into a single file, each SRA file was split into two ‘read’ files corresponding to the paired sequencing reads. In each pair of read files, paired reads were assigned the same sequence ID to enable reconstitution of native heavy/light pairs. Quality and length trimming was performed on each read file using Sickle [74] (with options -q 25 and -l 200) such that the 3' end of each read was trimmed until a 25-base sliding window contained an average sequence quality of 25 and trimmed reads of less than 200 bases were discarded. Germline V(D)J gene assignment and junction identification was performed with AbStar and resulting assignments were stored in a MongoDB database. Heavy-chain junctions were clustered at 96% sequence identity to collapse duplicate sequencing reads and centroid sequences were calculated for each cluster with at least 2 heavy chain junctions. For each centroid heavy chain sequence, the sequence ID was used to retrieve the appropriate paired light chain sequence. Length distributions for heavy, kappa light, and lambda light chains were measured using IMGT [75] conventions.

### HPV vaccine-induced antibodies

MAbs were cloned from single HPV 16-specific, CD27^+^IgD^-^ memory B cells as previously described [54]. In brief, PBMC samples were enriched for B cells, separated into two parts, and stained with a multicolor flow cytometry panel and either Alexa Fluor 488 (AF488)-conjugated HPV 16 pseudovirus (psV) or AF488-conjugated bovine papillomavirus psV (negative control). AF488-HPV 16 psV^+^ memory B cells were then single cell sorted by FACS into PCR plates containing lysis buffer. cDNA was generated from the bulk RNA of these sorted cells using RT-PCR with random primers. Full-length heavy and light chain variable regions were then separately amplified from the cDNA by conducting multiple PCRs in parallel with pools of newly designed primers against the leader and constant regions. For this study, we utilized the following PBMC samples: 1. De-identified PBMC collected one month post-final vaccine dose from women aged 9–13 and 16–26 years of age who received two or three doses of the quadrivalent HPV vaccine as part of a clinical trial [76](clinicaltrials.gov Identifier: NCT00501137); 2. PBMC collected one month post-final vaccine dose from women aged 18–26 years of age who received the full quadrivalent HPV vaccine series and were HPV 16 seronegative at the start of the study; 3. PBMC collected from women aged 27–45 years of age who were HPV 16 seropositive and had never been vaccinated against HPV.

### Anthrax vaccine-induced antibodies

Monoclonal antibodies were generated as previously described [77] from day 7 plasmablasts from individuals who had received anthrax vaccination. The antibodies were tested for reactivity against PA by ELISA.

### Ethics statement

For next generation sequencing of IgG+ memory B cells, peripheral blood was obtained from healthy adult donors following written informed consent, under a protocol (IRB# 12–5951) approved by the Scripps Institutional Review Board. For analysis of HPV vaccine-induced antibodies, sample group 1, as outlined above, was collected from de-identified participants in a clinical trial [76](clinicaltrials.gov Identifier: NCT00501137); sample groups 2 and 3 were collected with written informed consent from women enrolled in a study that was approved by the Institutional Review Boards of the University of Washington and the Fred Hutchinson Cancer Research Center (file numbers 42337 and 7740, respectively). For analysis of anthrax vaccine-induced antibodies, samples were collected from individuals who were recruited and consented in writing in accordance with the University of Chicago institutional review board (IRB #09-440-A).

### Antibody features frequency analysis

Frequency distributions were measured for multiple antibody features (see below) from two sets of human memory antibody sequences: one set of heavy-light paired sequences from three donors from DeKosky *et al*. [29] and another set of unpaired heavy and light chain sequences from eight donors from this study. Because each antibody chain was sequenced using only a single MiSeq read, the DeKosky *et al*. sequences were incomplete, generally spanning only from CDR2 through the J-chain on both the heavy and light chains. This meant that the DeKosky *et al*. sequences could not be used to measure distributions for several of the features. However, the DeKosky *et al*. paired sequences were essential for measuring the frequency distribution for V_L_ given V_H_. In total, after we filtered the DeKosky *et al*. sequencing data as described above, we were left with 127,701 paired sequences. From the unpaired sequences from eight donors determined for this study, we used 99,678 heavy-chain sequences and 52,560 light-chain sequences.

Frequency distributions for the following 15 features were measured, with the distributions shown in S1 Fig: (1) Heavy chain VDJ; (2) V_L_ given V_H_, or "V_L_|V_H_"; (3) J_L_ given V_L_, or "J_L_|V_L_"; (4) H-CDR3 length; (5) L-CDR3 length, computed separately for kappa or lambda light chains; (6) Percent amino-acid mutation on V_H_, or "V_H_mut"; (7) Percent amino-acid mutation on V_L_, or "V_L_mut"; (8) Ratio of framework % amino acid mutation to V_H_ gene % amino-acid mutation for heavy chains with different % V_H_ mutation levels, or "FR_H_mut/V_H_mut given V_H_mut" or most compactly written as "[FR_H_mut/V_H_mut]|V_H_mut"; (9) Ratio of framework % amino-acid mutation to V_L_ gene % amino-acid mutation for light chains with different % V_L_ mutation levels, or "FR_L_mut/V_L_mut given V_L_mut" or "[FR_L_mut/V_L_mut]|V_L_mut"; (10) sizes of insertions in human heavy chains with different % V_H_ gene mutation levels, or "InsSizes_H_|V_H_mut"; (11) sizes of deletions in human heavy chains with different % V_H_ gene mutation levels, or "DelSizes_H_|V_H_mut"; (12) sizes of insertions in human light chains with different % V_L_ gene mutation levels, or "InsSizes_L_|V_L_mut"; (13) sizes of deletions in human light chains with different % V_L_ gene mutation levels, or "DelSizes_L_|V_L_mut"; (14) number of cysteines in the Fv domain of human heavy chains with different % V_H_ gene mutation levels, or "CysCount_H_|V_H_mut"; (15) number of cysteines in the Fv domain of human light chains with different % V_L_ gene mutation levels, or "CysCount_L_|V_L_mut". (n.b. Most heavy or light chain Fv domains have two cysteines that form highly conserved disulfide bonds; therefore, counting the number of cysteines was used to track the potential formation of additional disulfide bonds.)

The features frequency (f) was computed as the product of the 15 individual frequencies:

f = f(VDJ) × f(V_L_|V_H_) × f(J_L_|V_L_) × f(V_H_mut) × f(V_L_mut) × f([FR_H_mut/V_H_mut]|V_H_mut) × f([FR_L_mut/V_L_mut]|V_L_mut) × f(InsSizes_H_|V_H_mut) × f(DelSizes_H_|V_H_mut) × f(InsSizes_L_|V_L_mut) × f(DelSizes_L_|V_L_mut) × f(CysCount_H_|V_H_mut) × f(CysCount_L_|V_L_mut), where each term in the equation represents a single frequency with a value greater than 0 and less than or equal to 1, except for the InsSizes and DelSizes terms which represent the product of the frequencies for each insertion or deletion, respectively, detected in a sequence. Bin sizes and mutation ranges used to compute the various frequency distributions were selected with an attempt to reveal variations in the data above random noise and to minimize the number of bins with zero counts in the data ranges needed to assess HIV bnAb sequences. In the cases of bins with zero counts, the AFF method assigns a frequency of 1/N, where N is the number of sequences used to measure the distribution in question. For example, if the AFF method is asked to evaluate the features frequency of an antibody sequence that has a V_H_D_H_J_H_ not observed in the 227,379 memory antibody heavy-chain sequences used to construct the VDJ frequency distribution, the value of f(VDJ) will be assigned as 1/227,379. For Monte Carlo generation of antibody sequences consistent with the AFF method, features were randomly selected from each feature distribution, using a pseudo-random number generator in awk. We found that the default output of the rand() function in awk, with six significant figures, contained repeats even in 10,000 trials. We combined rand() and sprintf() to generate numbers with ten significant figures [as random_number = sprintf("%.10g",1-rand())], and this method generated sequences of numbers with no repeats in 300,000 trials that were sufficiently evenly distributed over the interval from 0 to 1, with less than 1% variation in the frequency of numbers generated among all 20 intervals of size 0.05 (0<x≤0.05; 0.05 <x≤0.1; etc.).

### Feature independence in the AFF model

By computing the overall features frequency as a product of individual features frequencies, the AFF model makes the approximation that the 15 features frequencies are independent. As described above, we have attempted to construct the features in a manner that makes them independent, by accounting for expected dependencies or correlations (e.g. f(VDJ) accounts for correlations in usage of V_H_, D_H_ and J_H_ genes, f(V_L_|V_H_) accounts for V_L_ and V_H_ pairing preferences, f(J_L_|V_L_) accounts for J_L_ and V_L_ pairing preferences, and multiple features explicitly account for their dependence on % mutation albeit in a rather coarse manner dictated by limited available data). There are two notable exceptions, where we were unable to account for an expected dependence. The first is that V_H_mut and V_L_mut are treated as independent. In reality, as an antibody undergoes somatic hypermutation, both heavy and light chains are likely to gain mutations; hence, the % mutations on the heavy and light chains are likely to be correlated. The Pearson linear correlation coefficient for V_H_mut and V_L_mut computed over the 388 "normal" Abs is 0.49, and the Spearman rank correlation coefficient is 0.51, both indicating a modest but not strong linear correlation (S2A Fig). Therefore, treating V_H_mut and V_L_mut as independent modestly overestimates the frequency penalty due to mutation. At present, there are insufficient data available to allow parametrization of the dependence of V_L_mut on V_H_mut or vice versa. We noted above that the DeKosky *et al*. [29] heavy-light paired sequences were incomplete, generally spanning only from CDR2 through the J-chain on both the heavy and light chains; this meant that the DeKosky et al. data could not be used to compute V_H_mut or V_L_mut or their correlations. If similar NGS data on heavy-light paired sequences becomes available with complete sequences of heavy and light, then the AFF model could be improved by accounting for the correlation between V_H_mut and V_L_mut. We did test the effect of ignoring the V_L_mut term, which would correspond to underestimating the frequency penalty due to mutation; while the computed frequency for each antibody sequence increased because a penalty term was eliminated, the overall findings of AFF were qualitatively the same.

The second expected dependence that we could not explicitly account for in the AFF model is the dependence of H-CDR3 length on the V_H_, D_H_ and J_H_ genes. It has been shown that, in human peripheral blood antibodies, "long" H-CDR3 loops of length 24 aa or more, and "very long" H-CDR3 loops of length 28 aa or more, are more commonly found in antibodies utilizing J_H_ genes of the J6 family or D_H_ genes of the D2 or D3 families, and less commonly found in antibodies utilizing J_H_ genes of the J4 family [78]. We confirmed these trends in the 227,379 memory antibody heavy chain sequences obtained by NGS in this study and in DeKosky *et al*., finding that antibodies using D_H_2 or D_H_3 with J_H_6 had an H-CDR3 length distribution most shifted to longer lengths while antibodies using J_H_4 with neither D_H_2 nor D_H_3 had a distribution most shifted to shorter lengths (S2B Fig). Briney et al. [78] further showed that only a small subset of the D_H_2 or D_H_3 genes were favored for long H-CDR3s, and they also provided evidence that certain V_H_ gene families were favored or disfavored for long H-CDR3s. We lack sufficient sequencing data to quantify these effects and incorporate them into AFF at this time: even the coarse treatment shown in S2B Fig has too little data to accurately specify distributions at lengths above ~25 amino acids. Therefore, we are not accounting for these effects in AFF, even at this coarse level, at this time. We note that the magnitude of the frequency corrections that would be obtained by accounting for these dependencies is modest: in S2B Fig, over the range of H-CDR3 lengths of 20 to 25, the average frequency correction for Abs using D_H_2 or D_H_3 with J_H_6 would be to increase the frequency by a factor of 3.4 compared to using the distribution for all Abs, while the average correction for Abs using J_H_4 with neither D_H_2 nor D_H_3 would be to reduce the frequency by a factor of 7.1. Frequency corrections of these magnitudes would shift the log(f) values in Fig 1 by less than one unit. Thus, while the AFF model currently assigns a frequency based on the H-CDR3 length without incorporating any dependence on V_H_, D_H_ and J_H_ genes, incorporating these dependencies would improve the accuracy of the model.

To evaluate the degree to which the features frequencies might be linearly correlated, we computed the Pearson linear correlation coefficient "r" for each of the pairs of frequencies (frequencies 1 to 15 above, a total of 105 pairs), among the 388 "normal" Abs. The r-values ranged from -0.41 to +0.39, indicating that none of the pairs of frequencies were strongly linearly correlated. Indeed, six of the seven r values with absolute value greater than 0.2 each involved frequencies (of insertions or deletions) with very small data range (of standard deviation less than 0.1) indicating no meaningful correlation. Inspection of plots of frequency pairs for the seven largest absolute values of r failed to identify visually compelling correlations.

### Sensitivity analysis of the AFF model

To assess the degree to which the 15 individual terms in the AFF model contribute to the output, we conducted a global sensitivity analysis using as input the 300,000 mc antibody sequences. We measured the proportion of the total variance of log(f_HL) contributed by the variance in each individual log(f) term. The results are shown in S3 Fig. All of the terms contribute to varying degrees, with eight terms each contributing between 7 and 12% of the variance and seven terms each contributing between 2.5 and 5% of the variance.

### Protein production and purification: gp120

Full-length gp120 proteins were produced in FreeStyleTM 293F (Invitrogen) suspension cultures or lab-adapted 293S (GnTI^-/-^) suspension cultures by transient transfection using 293Fectin (Invitrogen) of a pHLSec plasmid containing gp120 with a C-terminal His_6x_ affinity tag. Protein was harvested from the supernatant after 96 h and purified by affinity chromatography with a HisTrap column (GE) followed by Superdex 200 size exclusion chromatography (GE Healthcare) using an AKTA Express system (GE Healthcare).

### Protein production and purification: eOD-N276Kif

eOD-N276Kif was produced in FreeStyleTM 293F (Invitrogen) suspension cultures by transient transfection using 293Fectin (Invitrogen) of a pHLSec plasmid containing gp120 with a C-terminal His_6_ affinity tag in the presence of 25 μM kifunensine. Protein was harvested from the supernatant after 96 h and purified by affinity chromatography with a HisTrap column (GE) followed by Superdex 75 size exclusion chromatography (GE Healthcare) using an AKTA Express system (GE Healthcare).

### Antibody production

IgG and Fabs were produced using the pFUSEss expression vectors or pHLsec, respectively, and purified as described previously [40].

### Development of MinVRC01: Library design and screening

VRC01 is extensively mutated from its putative germline precursor and we sought to identify all mutations from germline that contributed significantly to the function of the antibody. This was accomplished by generating yeast surface display libraries in which VRC01 variants were expressed on the surface of yeast as scFvs. In these libraries, positions containing somatic mutations in the V_H_ and V_L_ gene segments of VRC01 were allowed to sample either the germline or affinity-matured residues. VRC01 contains 41 mutations in the variable heavy (V_H_) and 23 mutations in the variable light (V_L_) gene segments. Due to limitations on the size of library generation for yeast surface display (the largest library that can be easily sampled is ~1x10^7^), it was not possible to generate a library that simultaneously sampled all possible combinations on the heavy chain and light chain (2^(41+23)^ = 1.8x10^19^); therefore, the library size had to be reduced by ~12 orders of magnitude.

The library size was reduced in two ways. First, the heavy chain and light chain were separated into two libraries, in which the heavy chain (HC) library was paired with the fully mature light chain (LC), and vice versa. Second, to further reduce the diversity on the HC, only positions that had mutated from the germline precursor in both VRC01 and PGV04 (the only VRC01-class bnAbs available to us when this study began) were considered for the library. Libraries were generated using PCR assembly of oligos containing degenerate codons, as described previously [40]. Both the HC_library/LC_Mat and HC_Mat/LC_library were sorted for binding to biotinylated YU2 gp120. Initially, a LC library was generated in which the two amino-acid deletion in L-CDR1 was removed. However, as high affinity clones failed to enrich, a second library containing the deletion was created and sorted. Sequences were recovered and any mutations that enriched above 60% were retained.

At this point, the library was small enough that the heavy and light chains could be combined together and all possible combinations sorted at the same time. Initially this library was sorted for binding to recombinantly produced YU2 gp120. Position 54 in the library was designed to encode Gly or Ser, but a number of recovered sequences contained a phenylalanine at that position; the phenylalanine presumably arose during PCR. A revised library was created, allowing position H54 to sample Gly, Ser and now Phe. This library was generated, split into 5 separate groups and independently sorted for binding to recombinant gp120 derived from UG037 (clade A), JR-FL (clade B), YU-2 (clade B), CN54 (recombinant clade BC) and DU179 (clade C) strains of HIV. Any position that enriched for the mature amino acid >67% of the time was included in MinVRC01.

### Development of MinVRC01: Library sorting method

Libraries displaying MinVRC01 scFvs were induced overnight, removed from the induction media, washed and labeled with monomeric gp120 for 1 hour. The cells were pelleted and washed once in PBS + 0.2% BSA, then labeled with an anti-c-Myc-FITC conjugated secondary to assess the amount of scFv on the surface and streptavidin PE to detect bound gp120. The top 10% binding population (indicated by the FITC:PE ratio) were sorted into selectable media and regrown to induce and sort again. This process was repeated 4–5 times at decreasing concentrations of gp120 to select the highest affinity clones. The initial library was sorted at a gp120 concentration of160 nM to generate a pool of clones that had properly assembled and displayed the scFv. In subsequent sorts, the concentration of gp120 was decreased such that higher affinity clones could be easily sorted from the majority of the binding population. This process was repeated until reducing the concentration of gp120 resulted in a global decrease in PE signal of all clones in the sort, indicating that all present variants had comparable affinity. The final sorts were typically done at a gp120 concentration of 10–40 nM. The initial GH/ML and MH/GL library was sorted against only YU2 gp120, and subsequent libraries were sorted against YU2, UG037, DU179 CN54 or JRFL gp120. After 5 sorts, cells were plated out onto selectable media and 48 sequences were recovered from each library.

### Development of Min12A21

To determine the generalizability of the MinVRC01 mutations, we set out to develop a second minimally mutated VRC01-class antibody. We selected 12A21 [33] as a good candidate because the mature 12A21 bnAb contains no insertions or deletions and has an aromatic residue at position 54 on the heavy chain. The sequence of 12A21 was aligned to MinVRC01 and the somatic hypermutations on 12A21 that appeared equivalent to those in MinVRC01 were preserved. We then generated a directed library for which the remaining positions were allowed to sample either the germline residue or the 12A21 mutation. This library was then sorted for binding to a single gp120. Two additional mutations, Arg^H19^ and Trp^H37^, were identified in the sorting and were therefore included in Min12A21.

### Polyreactivity assay: HEp-2 cell staining assay

The HEp-2 cell-staining assay was performed using kits purchased from Aesku Diagnostics (Oakland, CA). These Aesku slides use optimally fixed human epithelial (HEp-2) cells (ATCC) as substrate and affinity purified, FITC-conjugated goat anti-human IgG for the detection. The procedure followed the manufacturer's instructions. Briefly, 2.5 μg or 25 μl of 100 μg/ml mAb and controls were added to wells and incubated on HEp-2 slides in a moist chamber at room temperature for 30 min. Slides were then rinsed and submerged in PBS. Excess PBS was shaken off and 25 μl of FITC-conjugated goat anti-human IgG was immediately applied to each well. Slides were allowed to incubate at room temperature in a moist chamber for another 30 min. Slides were next washed in the same manner as above and then mounted on coverslips using the provided mounting medium. Slides were viewed at 20x magnification and photographed on an EVOS f1 fluorescence microscope at a 250 ms exposure with 100% intensity. Sera of positive and negative controls were provided by the vendor. Samples showing fluorescence greater than the negative control were considered positive for HEp-2 staining.

### Polyreactivity assay: Polyspecificity reagent (PSR) binding assay

Monoclonal antibodies were screened for reactivity with preparations of solubilized membrane proteins (SMP) and cytosolic proteins (SCP) as described previously [61, 62] with a small modification. Briefly, SMP and SCP were extracted from CHO cells (ATCC). The protein concentration was determined using the Dc-protein assay kit (BioRad). SMP and SCP were then immobilized on ELISA plates for mAb screening. The results were established by reading the absorbance at 450 nm of the examined samples.

### Polyreactivity assay: Cardiolipin binding

An Anti-Cardiolipin ELISA kit (Diamedix Corp, Miami Lakes, FL) was applied to test mAbs for cardiolipin reactivity according to the manufacturer’s instructions. Antibodies were used at concentrations starting at 50 μg/ml and titrated in two fold serial dilutions. 4E10 was the positive control and Humira was the negative control.

### Polyreactivity assay: Single autoantigen reactivity

Single antigen ELISA assays for SSA/Ro, SS-B/La, Sm, ribonucleoprotein (RNP), Jo-1, double-stranded DNA, centromere B, and histones were purchased from Aesku Diagnostics (Oakland, CA). The 96 wells were separately coated with these eight cellular and nuclear antigens for the qualitative detection of mAbs reactivity. A cut-off calibrator was provided by the manufacturer. The negative control was diluted human serum.

### Pseudovirus neutralization assay

Pseudoviruses were generated by transfection of 293T cells (ATCC) with an HIV-1 Env expressing plasmid and an Env-deficient genomic backbone plasmid (pSG3ΔEnv), as described previously [57]. Pseudoviruses were harvested 72 h post-transfection for use in neutralization assays. Neutralizing activity was assessed using a single round of replication in a pseudovirus assay with TZM-bl target cells. Briefly, TZM-bl cells were seeded in a 96-well flat bottom plate. To this plate was added pseudovirus, which was preincubated with serial dilutions of antibody for 1 h at 37°C. Luciferase reporter gene expression was quantified 72 h after infection upon lysis and addition of Bright-Glo Luciferase substrate (Promega). To determine IC_50_ values, dose response curves were fitted using nonlinear regression.

### VRC01-eOD-N276Kif crystallization

VRC01 Fab was produced and purified as previously described [40]. eOD-N276Kif, a minimal glycan, alanine-resurfaced construct possessing only three glycosylation sites (N18, N65 and N79, eOD numbering as in PDB IDs 4JPJ and 4JPK) was designed and subsequently transfected in HEK 293F (Invitrogen) suspension cells in the presence of the mannosidase I inhibitor kifunensine, to yield homogeneous Man_9_GlcNAc_2_ carbohydrates. The secreted protein was purified via its C-terminus His_6_-tag by affinity chromatography using HisTrap nickel columns (GE Healthcare), and subsequently purified to size homogeneity by Superdex 200 gel filtration chromatography (GE Healthcare). After incubation of VRC01 Fab in molar excess of eOD-N276Kif, the complex was purified by Superdex 200 size exclusion chromatography (GE Healthcare) and concentrated to ~5 mg/ml for crystallization trials using the automated JCSG/IAVI/TSRI CrystalMation robotic system (Rigaku) at the Joint Center for Structural Genomics (www.jcsg.org) at TSRI. Crystals used for data collection were obtained with 0.16 M ammonium sulfate, 20% (w/v) PEG4000, 20% (v/v) glycerol, 0.08 M sodium acetate, pH 4.6, as the mother liquor. Prior to data collection, crystals were cryoprotected in the mother liquor supplemented with 40% glycerol and subsequently fast-plunged into liquid nitrogen.

### BG505 SOSIP + NIH45-46 scFv + PGT122 Fab crystallization

The BG505 SOSIP.664 Env construct was expressed in HEK 293S GnTI^-/-^ cells, together with co-transfection with the furin protease, and purified as previously described [63]. The HEK 293S cells lack N-acetylglucosaminyltransferase I and, therefore, only produce glycoproteins bearing mannose-rich (Man_5-9_) glycans. Purified SOSIP.664 gp140 trimers were mixed in molar excess of the PGT122 Fab, and subsequently treated with EndoH (New England BioLabs), resulting in a partially deglycosylated glycoprotein, as previously described [63, 79]. Following partial deglycosylation, the complex was incubated with a molar excess of NIH45-46 scFv and purified to size homogeneity using a Superose 6 10/30 gel filtration column (GE Healthcare). Partial deglycosylation prior to addition of NIH45-46 scFv was critical to ensure complete saturation of the trimer, as previously described [48]. The purified complex was concentrated to ~5 mg/ml and screened in crystallization trials using the Oryx8 crystallization robot (Douglas Instruments). Crystals were readily obtained from a crystallization condition containing 2.4 M ammonium sulfate, 0.1 M Tris, pH 8.0. After dehydration and flash freezing, three crystals diffracted particularly well and the x-ray data collected were merged to obtain a high-redundancy complete data set to 4.4 Å (S7 Table). The resolution limit was determined based on I/σ > 2.0, the XSCALE-determined CC_1/2_ significant threshold [80], and overall quality of the electron density maps calculated to slightly varying resolutions.

### Crystallographic data processing and structural determination

Data processing was performed using XDS [80]. Statistics for data collection and processing are reported in S7 Table. The VRC01-eOD-N276Kif crystal structure was solved using the coordinates from PDB ID 4JPK as a search model for molecular replacement in PHASER [81]. To solve the partially deglycosylated BG505 SOSIP-NIH45-46 scFv–PGT122 Fab crystal structure, coordinates from PDB IDs 4NCO and 3U7Y were used as search models for molecular replacement using PHASER [81]. Refinement proceeded with non-crystallographic symmetry (NCS) and secondary structure restraints. For both structures, refinement was performed using a combination of PHENIX [82] and COOT [83]. Refinement statistics are reported in S7 Table.

### Molecular dynamic simulations: Starting structures

The crystal structure of mature VRC01 in complex with eOD containing a Man_9_GlcNAc_2_ glycan (Fig 5A and 5B and PDB ID 5KZC) was used as the template to prepare starting structures for molecular dynamics simulations.

### Preparation of the complex between eOD (with complex glycans) and mature VRC01

The core-fucosylated biantennary complex glycan [Gal β1–4 GlcNAc β1–2 Man α1–6 (Gal β1–4 GlcNAc β1–2 Manα1–3) Man β1–4GlcNAc β1–4 (Fuc α1–6) GlcNAcβ1] was obtained from the BiOligo database (http://glyco3d.cermav.cnrs.fr) [84] and edited to match the GLYCAM06h [85] force-field parameters for glycans and ff99SB [86] for protein as per AMBER12 [87] to generate the coordinate and topology files for the starting structures. The TIP3P explicit water model [88] was employed for solvation using periodic boundary conditions and chloride ions were added to neutralize the system using LEAP following standard protocols [89].

### Molecular dynamics simulations

Starting with the prepared mature VRC01-eOD complex containing the core-fucosylated biantennary N-glycan system, energy minimization was performed in two steps; first, to remove the initial unfavorable contacts made by the solvent, and, second, to minimize the entire system as a whole, prior to the molecular dynamics (MD) simulations. A stepwise protocol was employed for equilibration, beginning with a simulation under constant volume (NVT) conditions for 300 ps switching to constant pressure (NPT) conditions at 1 atm for a further 500 ps. All simulations were performed at 300 K. MD simulations were then performed on the prepared systems in explicit solvent for 30 ns with a time step of 2 fs. The co-crystal structure of mature VRC01-eOD with the Man_9_GlcNAc_2_ glycan on N276 was used as a control for the MD simulations, which, when compared to the simulated structure, did not reveal significant deviation, hence validating the protocol used. Ten simulations each, for the complexes between the mature VRC01 and the eOD containing a Man_9_GlcNAc_2_ or the core-fucosylated biantennary N-glycan at N276, were initiated and these uncorrelated simulations were used to demonstrate data reproducibility. Further, simulations of the unliganded eOD containing a Man_9_GlcNAc_2_ or the core-fucosylated biantennary N-glycan were performed to study the range of conformational sampling of glycan species that could be present at N276 of the antigen and the restrictions that antibody binding imposes upon its conformational space.

## Supporting Information

S1 Fig Parametrization of the AFF model. (**A**) V_H_D_H_J_H_ frequencies in human memory antibodies are shown for all 5,752 V_H_D_H_J_H_ combinations observed in 227,379 memory antibody heavy-chain sequences from this study and from DeKosky *et al*. 2015 [29] (blue), and for 3×10^5^ antibody sequences generated by a Monte Carlo method (red). (**B**) The same frequency distributions as in (A), but showing only the 100 most frequently observed V_H_D_H_J_H_ combinations. (**C**) Example frequency distributions for V_L_ paired with V_H_3-23D (blue), from 127,701 heavy-light paired sequences from DeKosky *et al*. 2015 [29], and for sequences generated by a Monte Carlo method (red). (**D**) Example frequency distributions for V_L_ paired with V_H_1-2, obtained as in (C). (**E**) Example frequency distributions for J_L_ paired with Vκ3–20 (blue), from 180,261 human memory B cell light chain sequences from this study and DeKosky *et al*. 2015 [29], and for sequences generated by a Monte Carlo method (red). (**F**) Example frequency distributions for J_L_ paired with Vκ1D-33 (blue), obtained as in (**E**). (**G**) Example frequency distributions for J_L_ paired with Vλ2–14 (blue), obtained as in (**E**). (**H**) H-CDR3 length distributions for memory heavy chain sequences obtained by NGS (blue circles and line) or from sequences generated by a Monte Carlo method (black line), both as in (A), or from 388 "normal" human antibodies isolated by B cell sorting from multiple sources (see Fig 1 caption) (gray diamonds). (**I**) Kappa chain L-CDR3 length distributions. (**J**) Lambda chain L-CDR3 length distributions. (**K**) Frequency distributions of V_H_ gene % amino-acid mutation. (**L**) Frequency distributions of V_L_ gene % amino-acid mutation. The somewhat higher mutation levels in the "normal" Abs reflect the relatively few "normal" Abs in the sample (388, minimum detectable frequency is 2.6×10^−3^ = 1/388) and also may reflect the fact that all but the Tiller *et al*. [56] antibodies were affinity-selected either by antigen-specific B cell sorting [51–54] or by direct affinity measurements on recombinant antibodies after cloning from plasmablast B cells [55]). (**M**) Distribution of the ratio of framework % mutation to V_H_ gene % mutation for V_H_ gene amino-acid mutation levels between 0 and 5%. (**N**) Same as in (**M**) but for V_H_ gene amino-acid mutation levels between 5 and 10%. (**O**) Same as in (M) but for V_H_ gene amino-acid mutation levels between 10 and 100%. The somewhat higher ratios in the "normal" Abs may reflect similar effects as noted in (**K**). (**P**) Distribution of the ratio of framework % mutation to V_L_ gene % mutation for V_L_ gene amino-acid mutation levels between 0 and 5%. (Q) Same as in (**P**) but for V_L_ gene amino-acid mutation levels between 5 and 100%. The somewhat higher ratios in the "normal" Abs may reflect similar effects as noted in (**K**). **(R)** Distribution of sizes of insertions in human heavy chains with different % V_H_ gene amino-acid mutation levels. **(S)** Same as in (**R**) but for different sizes of deletions. **(T)** Distribution of different sizes of insertions in human light chains with different % V_L_ gene amino-acid mutation levels. **(U)** Same as in (**T**) but for sizes of deletions. Most of the data points for the "normal" antibodies in (**R**), (**S**), (**T**) and (U) are at insertion size = 0 or deletion size = 0. **(V)** Distribution of the number of cysteines in the Fv domain of human heavy chains with different % V gene amino-acid mutation levels. **(W)** Same as in (**V)** but for light chains.
(PDF)

S2 Fig Parameter dependencies within the AFF model. (**A**) Plot of the % amino acid mutation in V_L_ versus V_H_ for 388 normal Abs. A modest correlation can be seen, corresponding to a Pearson linear correlation coefficient of 0.49 and a Spearman rank correlation coefficient of 0.51. However, as this is not a strong correlation, the V_H_mut level is not a strong predictor of the V_L_mut level: a V_H_mut level of ~5.2% is accompanied by V_L_mut levels ranging from 0% to 11.4%, while a V_H_mut level of ~9.5% is accompanied by V_L_mut levels ranging from 1.1% to 12.2%. (**B**) H-CDR3 length distributions for human memory antibodies utilizing different D_H_ and J_H_ gene families. The blue curve shows the distribution for all 227,379 memory antibody heavy-chain sequences obtained by NGS in this study and in DeKosky *et al*. 2015 [29]. The red curves (solid or dashed) are for Abs using J_H_4, the green curves (solid or dashed) for Abs using J_H_6, and the black curves (solid or dashed) for Abs using J_H_ genes other than J_H_4 or J_H_6. The solid curves (red, green or black) are for Abs using D_H_2 or D_H_3 genes, and the dashed curves (red, green, or black) for Abs using D_H_ genes other than D_H_2 or D_H_3. In accordance with Briney et al. [78], the distribution most shifted toward longer H-CDR3 length is for Abs using D_H_2 or D_H_3 genes and using J_H_6, while the distribution most shifted toward shorter H-CDR3 lengths is for Abs using J_H_4 with neither D_H_2 nor D_H_3. For a given H-CDR3 length, the frequency correction obtained by using a distribution specifying D_H_ and J_H_ gene families is typically less than a factor of 10 compared to using the distribution for all human memory Abs.
(PDF)

S3 Fig Sensitivity analysis of the AFF model. Proportion of the total variance in log(f_HL_) due to each individual log(f) (the log of the frequency for an individual feature) is shown, computed from antibody sequences generated by a Monte Carlo method.
(PDF)

S4 Fig Features frequency analysis of HIV bnAbs. (**A**) VRC01-class bnAbs. (**B**) Minimally mutated and other engineered VRC01-class bnAbs. (**C**) 10E8 (solid symbol in Fig 1A.) (**D**) Less potent variants of 10E8 with reduced mutation (open symbols in Fig 1A). (**E**) Potent V2Apex bnAbs (solid symbols in Fig 1A). (**F**) Less potent V2Apex bnAbs (open symbols in Fig 1A). (**G**) CH103-class bnAbs. (**H**) PGT121-class bnAbs. (**I**) Very potent high mannose patch bnAbs isolated from infected individuals (solid symbols in Fig 1A). (**J**) 32H3L, a very potent high mannose patch bnAb engineered to use less mutated heavy and light chains from the PGT124 lineage identified by NGS by Sok et al. [40]. (**K**) Less potent high mannose patch bnAbs (open symbols in Fig 1A). (**L**) V_H_1-46 bnAbs. (**M**) VRC16. (**N**) 8ANC195. (**O**) 35O22. (**P**) VRC13.
(PDF)

S5 Fig Features frequency analysis of anti-influenza bnAbs. The features and their associated frequencies are given for all the antibodies analyzed in Fig 1. Descriptions of each feature and frequency are shown in the leftmost columns. Values for each antibody are shown as a column.
(PDF)

S6 Fig Polyreactivity assays. (**A**) Cardiolipin binding assay. The indicated antibodies were assayed for cardiolipin binding at the concentrations shown, with 4E10 as a positive control and Humira as a negative control. (**B**) Single antigen binding assay. The indicated antibodies were assayed for binding to eight nuclear and cellular auto-antigens at an antibody concentration of 50 μg/mL. Values within 20% of the cut-off calibrator score as equivocal. **(C)** Assessment of the effect of introducing Phe54 into VRC01 (by the G54F mutation) or removing Phe54 from MinVRC01 (by the F54S mutation), by the Hep2 cell binding assay. (**D**) Assessment of the effects of Phe54 as in (**C**), by the Polyspecificity reagent (PSR) binding assay; positive controls, 4E10 and 2F5; negative controls, Humira, 10E8, and PG9.
(PDF)

S7 Fig Mutation patches in VRC01-class bnAbs analogous to those in MinVRC01 and Min12A21. (A) Alignment of VRC01-class antibody heavy chains and the inferred germline precursor V_H_1-2*02, which highlight the regions that align to MinVRC01 patches. (**B**) Alignment of VRC01-class antibody light chains and their inferred germline precursors, which highlight the regions that align to MinVRC01 patches. Patches in (A) and (B) are colored according to the scheme used in Fig 2.
(PDF)

S8 Fig Comparison of N276 glycan conformations and interactions with VRC01-class bnAbs in two crystal structures. (**A**) The crystal structure of eOD-N276Kif containing a Man_9_GlcNAc_2_ glycan at N276 in complex with VRC01 expands our understanding of this critical interaction. (**B**) For comparison, the crystal structure of the gp120 core containing a Man_4_GlcNAc_2_ glycan at N276 is shown in complex with NIH45-46m2 (PDB ID: 4JKP) [47]. Rendered as in Fig 5B.
(PDF)

S9 Fig Predicted interactions with the N276 glycan. (**A**) Predicted clashes of germline-VRC01 with the N276 glycan. Superposition of the germline-VRC01/germline-targeting eOD-GT6 crystal structure (PDB ID: 4JPK, blue surface) on the VRC01-eOD-N276Kif crystal structure reveals potential clashes (red stars) of germline-VRC01 LFW3 and L-CDR1 loops (tubes) with the N276 glycan (yellow sticks). (**B)** Affinity maturation in VRC01 (gray surface) results in slight conformational changes in L-FW3 and a shorter L-CDR1, which allows interactions with the N276 glycan. The eOD main chain is colored green and shown in a tube representation. (**C**) Predicted interaction of various VRC01-class bnAbs with the N276 glycan. Superposition of crystal structures of core gp120 in complex with CD4bs bnAbs VRC01 (blue, PDB ID: 3GNB), PGV04 (orange, PDB ID: 4I3S), NIH45-46 (red, PDB ID: 3U7Y) and 3BNC117 (magenta, PDB ID: 4LSV) on the VRC01 (gray)-eOD-N276Kif (green) crystal structure reveals a similar mode of recognition of the N276 glycan (yellow sticks) for all these antibodies. (**D**) Predicted interaction of other VRC01-class bnAbs with the N276 glycan. Superposition of crystal structures of core gp120 in complex with CD4bs bnAbs VRC23 (light green, PDB ID: 4J6R), VRC03 (purple, PDB ID: 3SE8), and 12A21 (cyan, PDB ID: 4JPW) on the VRC01 (gray)-eOD-N276Kif (green) crystal structure indicates significant differences in the position of L-CDR1 and L-FW3 loops, suggesting a slightly different mode of recognition of the N276 glycan (yellow sticks) for these antibodies compared to VRC01. However, the position of these loops in the absence of N276 might be influenced by crystal packing and they might, therefore, adopt a different conformation in the liganded form.
(PDF)

S10 Fig Comparison of the conformational sampling of two N276 glycoforms on eOD in the free and VRC01-bound state. **(A**) Superposition of structures generated by molecular dynamics (MD) simulations of the unliganded eOD (gray) shows the area sampled by the Man_9_GlcNAc_2_ at N276. The corresponding root mean square fluctuation (RMSF in Å) of each glycan residue is described with a color code over its Consortium for Functional Glycomics (CFG) symbol representation. The high RMSF observed here shows the high flexibility of the glycans represented by a variety of conformations acquired by the Man_9_GlcNAc_2_ on the unliganded state of eOD. (**B**) Superposition of eOD (with Man_9_GlcNAc_2_)-VRC01 complex structures from MD clearly illustrates the restriction on the allowed conformational space of Man_9_GlcNAc_2_ when VRC01 (cyan) binds, as also reflected in the RMSF values. (**C)** Superposition of eOD with a core-fucosylated biantennary complex glycan [Gal β1–4 GlcNAc β1–2 Man α1–6 (Gal β1–4 GlcNAc β1–2 Manα1–3) Man β1–4GlcNAc β1–4 (Fuc α1–6) GlcNAcβ1] at N276 from MD simulations in the unliganded state shows the glycan sampling a large conformational space with high RMSF values. (**D)** The core-fucosylated biantennary complex glycan is restricted to a narrow conformational space upon VRC01 binding.
(PDF)

S1 Table Minimally mutated variants of VRC01 (MinVRC01) and 12A21 (Min12A21) show high retention of neutralization breadth and potency compared to affinity-matured antibodies. VRC01, MinVRC01, 12A21, and Min12A21 were tested on a cross-clade 80-virus panel. Values are neutralization IC_50_ in μg/ml and are colored according to the legend.
(PDF)

S2 Table Reversion of affinity maturation patches on VRC01 and MinVRC01 reveals redundancies in affinity maturation residues that contribute to neutralization. Variants in which affinity maturation patches on VRC01 and MinVRC01 were reverted to germline were tested on a cross-clade panel of viruses. While there was loss of neutralization breadth for L-FW3 and H-CDR2 reversions for MinVRC01, neutralization activity remained for VRC01. The data also show that the extra disulfide between H-CDR1 residue 32 and H-CDR3 residue 98 is not necessary for potency and breadth of MinVRC01. Presented values are neutralization IC_50_ in μg/ml and colored according to the listed legend.
(PDF)

S3 Table Buried Surface Area (BSA) on gp120 calculated from different co-crystal structures with VRC01-class bnAbs. gp120-CD4, PDB ID: 1GC1; gp120-VRC01, PDB ID: 3NGB; gp120-NIH45-46, PDB ID: 4JKP. BSA was calculated using 'Protein interfaces, surfaces and assemblies' service PISA at the European Bioinformatics Institute. (http://www.ebi.ac.uk/pdbe/prot_int/pistart.html) [90]. Areas shaded in blue correspond to elements present in eOD.
(PDF)

S4 Table BG505 glycan mutants. MinVRC01 patch revertants were tested for neutralization on BG505 mutant viruses. (Top) Values are neutralization IC_50_ in μg/ml and colored according to the listed legend. (Below) Values are fold-enhancement in neutralization potency compared to wild-type virus. LT, low titer. NA, not available due to low titer.
(PDF)

S5 Table The light chain of VRC01-class antibodies undergoes somatic hypermutation to accommodate the N276 glycan on Env. The glycan site at N276 was removed by alanine mutagenesis (N276A) on a cross-clade 13-virus panel and tested for neutralization compared to the corresponding virus without the glycan site removed (wild-type, WT). Virus panels were tested for neutralization by chimeric VRC01-class antibodies containing a mature heavy chain and germline light chain. Values are neutralization IC_50_ in μg/ml and colored according to the legend.
(PDF)

S6 Table Removal of the N276 glycan site predominantly affects VRC01-class antibodies. The glycan site at N276 was removed by alanine mutagenesis on a cross-clade 6-virus panel and tested for neutralization by VRC01 class antibodies as well as CD4 IgG_2_, other CD4bs bnAbs (b12), and CD4bs non-broadly neutralizing antibodies (b6, F105). Values are neutralization IC_50_ in μg/ml and colored according to the legend.
(PDF)

S7 Table X-ray data collection and refinement statistics. (PDF)
