# Correction: Minimally Mutated HIV-1 Broadly Neutralizing Antibodies to Guide Reductionist Vaccine Design

**DOI:** 10.1371/journal.ppat.1005905

**Published:** 2016-09-14

**Authors:** Joseph G. Jardine, Devin Sok, Jean-Philippe Julien, Bryan Briney, Anita Sarkar, Chi-Hui Liang, Erin M. Scherer, Carole J. Henry Dunand, Yumiko Adachi, Devan Diwanji, Jessica Hsueh, Meaghan Jones, Oleksandr Kalyuzhniy, Michael Kubitz, Skye Spencer, Matthias Pauthner, Karen L. Saye-Francisco, Fabian Sesterhenn, Patrick C. Wilson, Denise A. Galloway, Robyn L. Stanfield, Ian A. Wilson, Dennis R. Burton, William R. Schief

There are two errors in the author list. The seventh author’s name is incorrect. The correct name is Erin M. Scherer. The twentieth author’s name is incorrect. The correct name is Denise A. Galloway.
