# Supplementary material for: Minimally Mutated HIV-1 Broadly Neutralizing Antibodies to Guide Reductionist Vaccine Design
Source: PLoS Pathog. 2016 Aug 25;12(8):e1005815. doi: 10.1371/journal.ppat.1005815 (PMC4999182; doi:10.1371/journal.ppat.1005815)
Supplement: S1 Fig — (A) VHDHJH frequencies in human memory antibodies are shown for all 5,752 VHDHJH combinations observed in 227,379 memory antibody heavy-chain sequences from this study and from DeKosky et al. 2015 [29] (blue), and for 3×10^5^ antibody sequences generated by a Monte Carlo method (red). (B) The same frequency distributions as in (A), but showing only the 100 most frequently observed VHDHJH combinations. (C) Example frequency distributions for VL paired with VH3-23D (blue), from 127,701 heavy-light paired sequences from DeKosky et al. 2015 [29], and for sequences generated by a Monte Carlo method (red). (D) Example frequency distributions for VL paired with VH1-2, obtained as in (C). (E) Example frequency distributions for JL paired with Vκ3–20 (blue), from 180,261 human memory B cell light chain sequences from this study and DeKosky et al. 2015 [29], and for sequences generated by a Monte Carlo method (red). (F) Example frequency distributions for JL paired with Vκ1D-33 (blue), obtained as in (E). (G) Example frequency distributions for JL paired with Vλ2–14 (blue), obtained as in (E). (H) H-CDR3 length distributions for memory heavy chain sequences obtained by NGS (blue circles and line) or from sequences generated by a Monte Carlo method (black line), both as in (A), or from 388 "normal" human antibodies isolated by B cell sorting from multiple sources (see Fig 1 caption) (gray diamonds). (I) Kappa chain L-CDR3 length distributions. (J) Lambda chain L-CDR3 length distributions. (K) Frequency distributions of VH gene % amino-acid mutation. (L) Frequency distributions of VL gene % amino-acid mutation. The somewhat higher mutation levels in the "normal" Abs reflect the relatively few "normal" Abs in the sample (388, minimum detectable frequency is 2.6×10−3 = 1/388) and also may reflect the fact that all but the Tiller et al. [56] antibodies were affinity-selected either by antigen-specific B cell sorting [51–54] or by direct affinity measurements on recombinant antibodie [file ppat.1005815.s001.pdf]

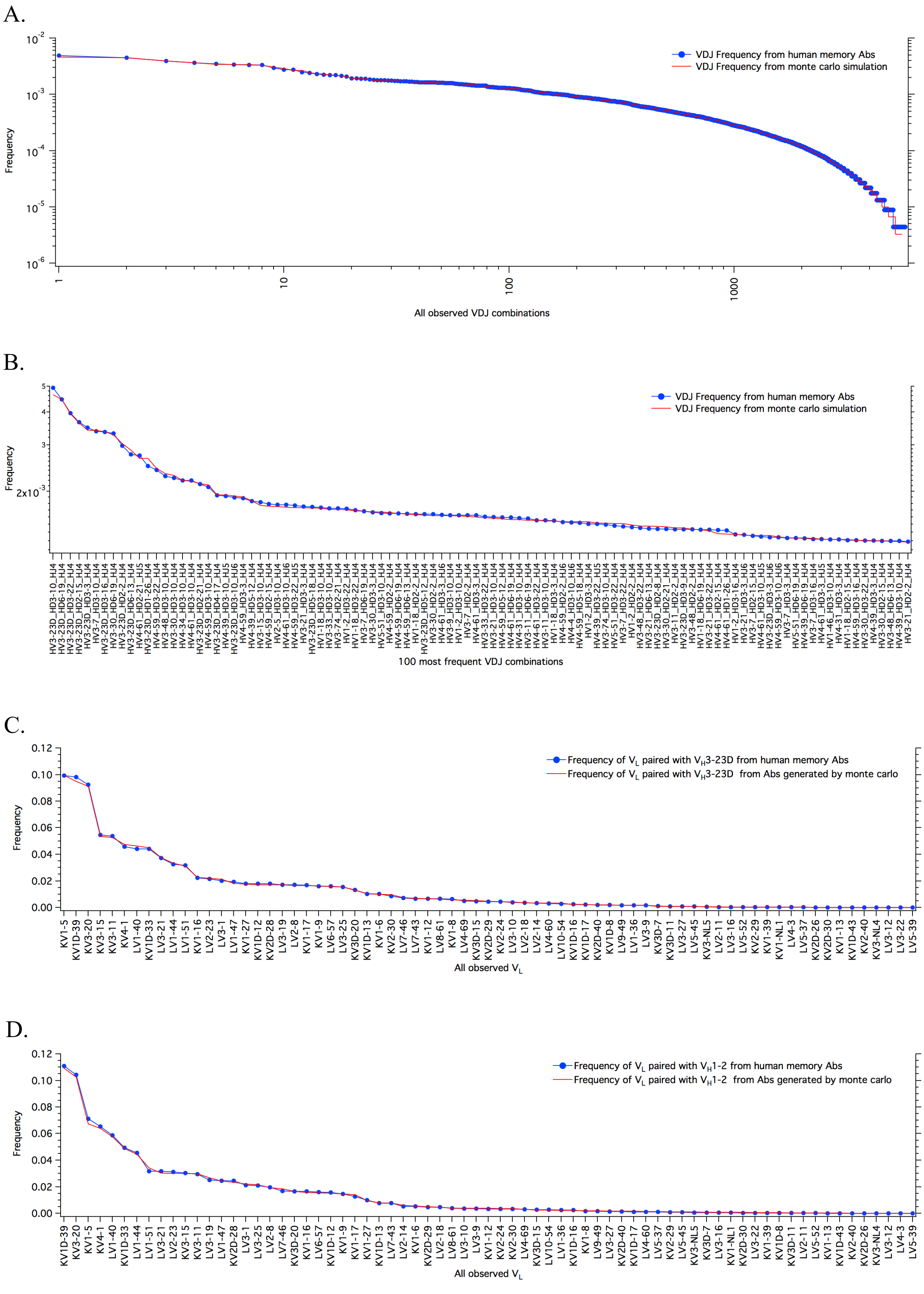
A.
VDJ Frequency from human memory Abs
VDJ Frequency from monte carlo simulation
Frequency
All observed VDJ combinations
B.
VDJ Frequency from human memory Abs
VDJ Frequency from monte carlo simulation
Frequency
100 most frequent VDJ combinations
C.
Frequency of V_L paired with V_H3-23D from human memory Abs
Frequency of V_L paired with V_H3-23D from Abs generated by monte carlo
Frequency
All observed V_L
D.
Frequency of V_L paired with V_H1-2 from human memory Abs
Frequency of V_L paired with V_H1-2 from Abs generated by monte carlo
Frequency
All observed V_L

E.

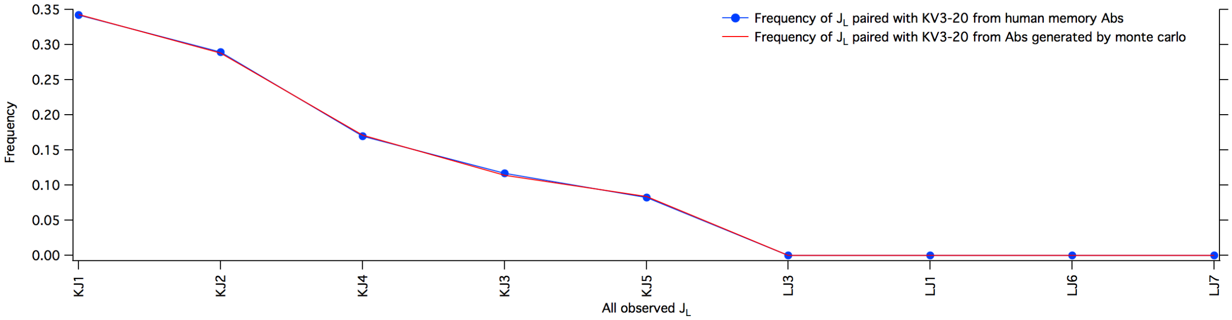

F.

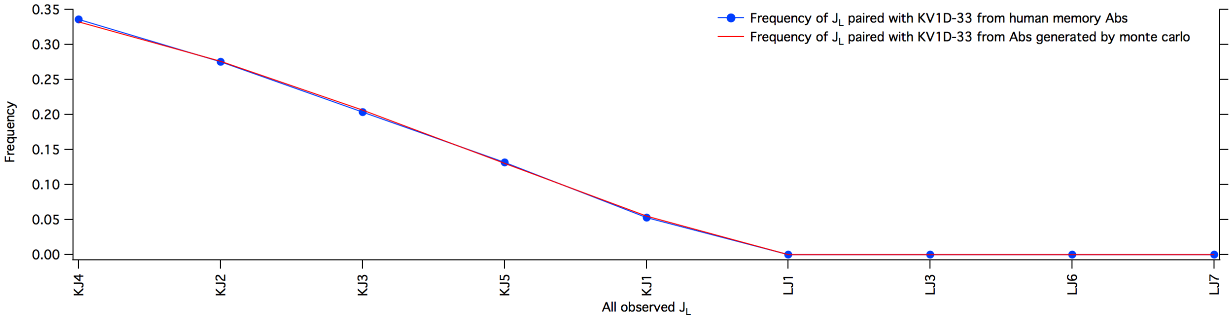

G.

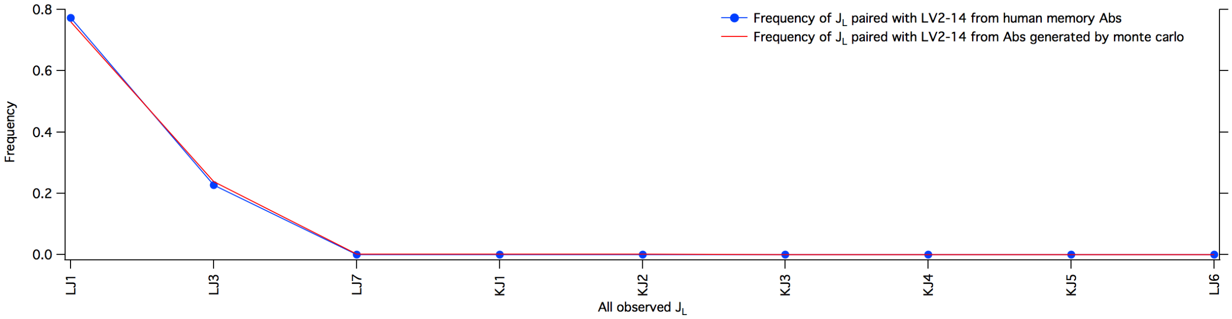

Fig. S1 (continued).

H.

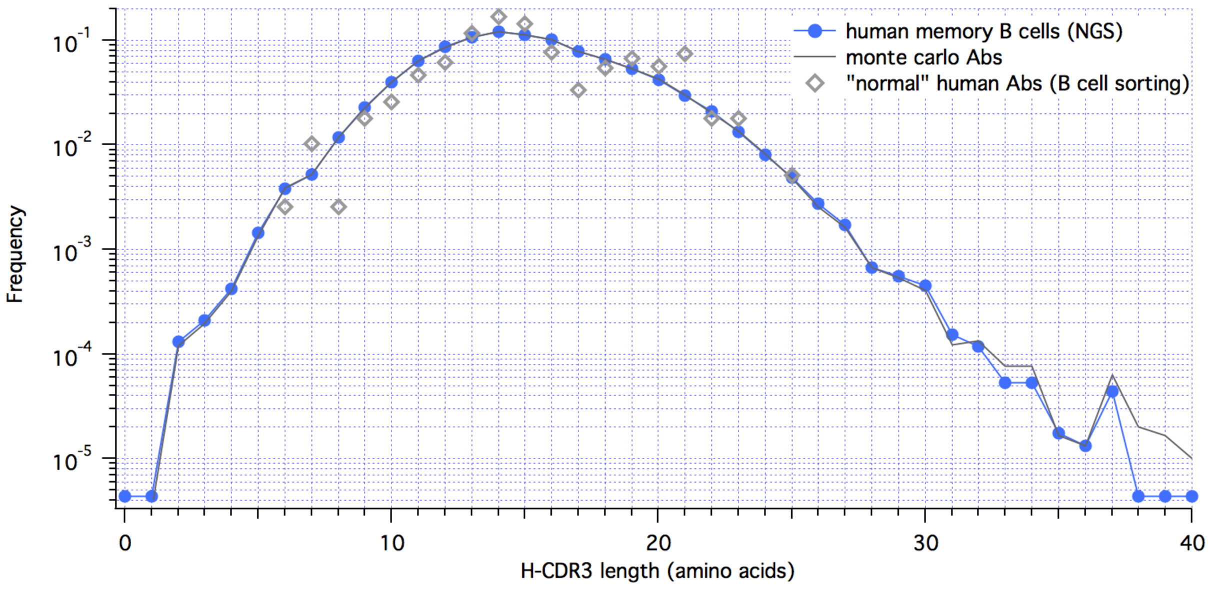

Fig. S1 (continued).

I.

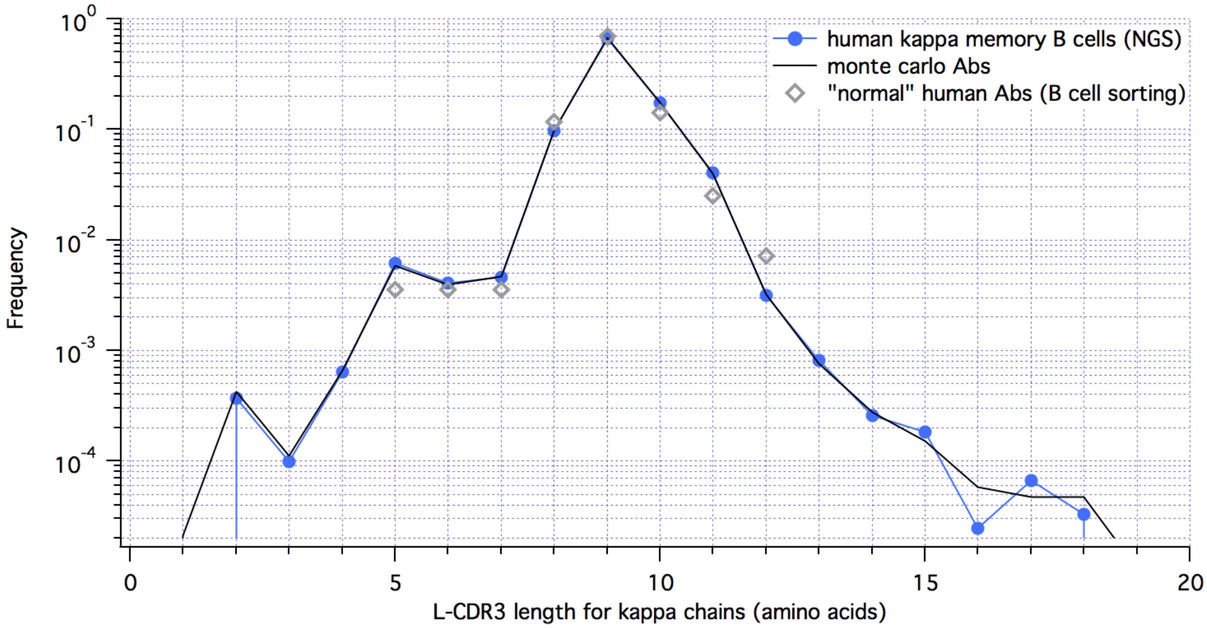

J.

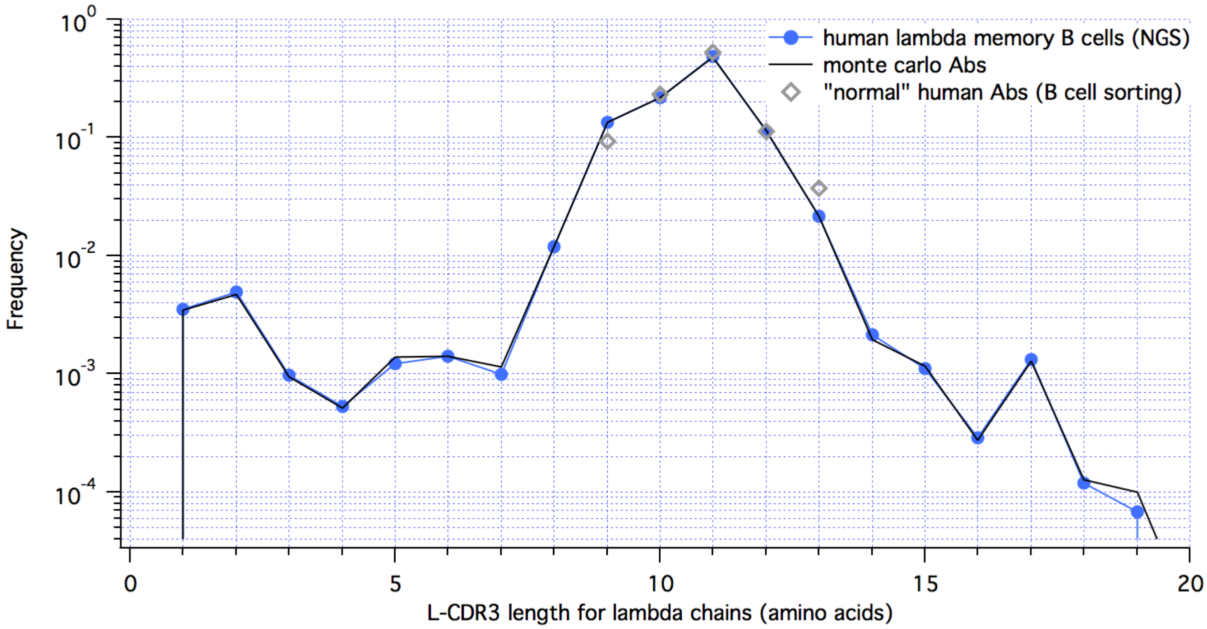

Fig. S1 (continued).

K.

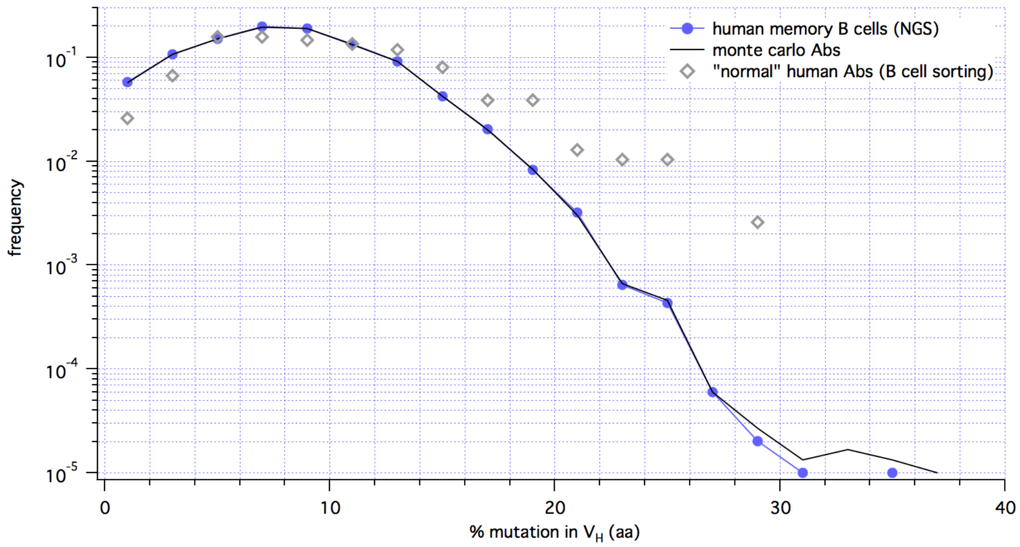

L.

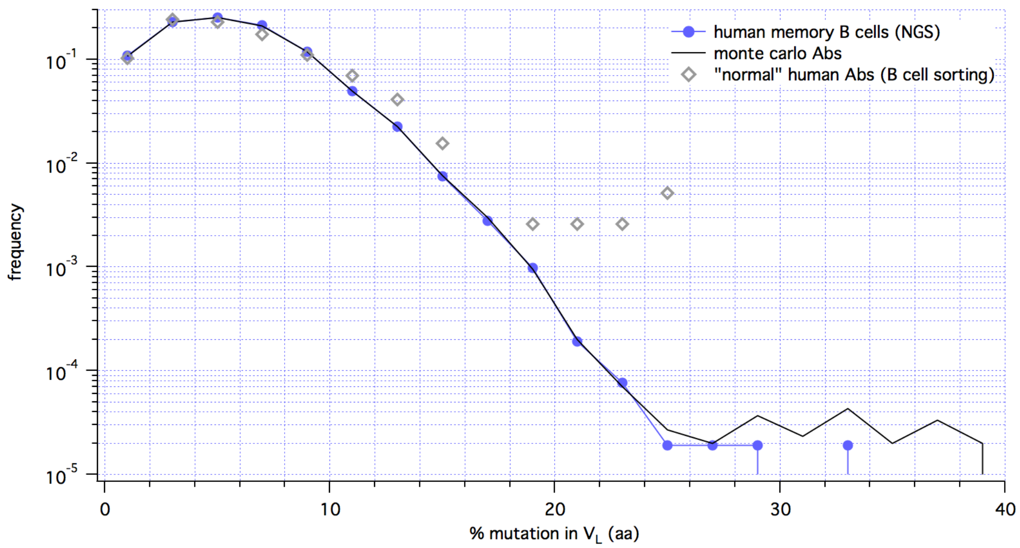

Fig. S1 (continued).

M.

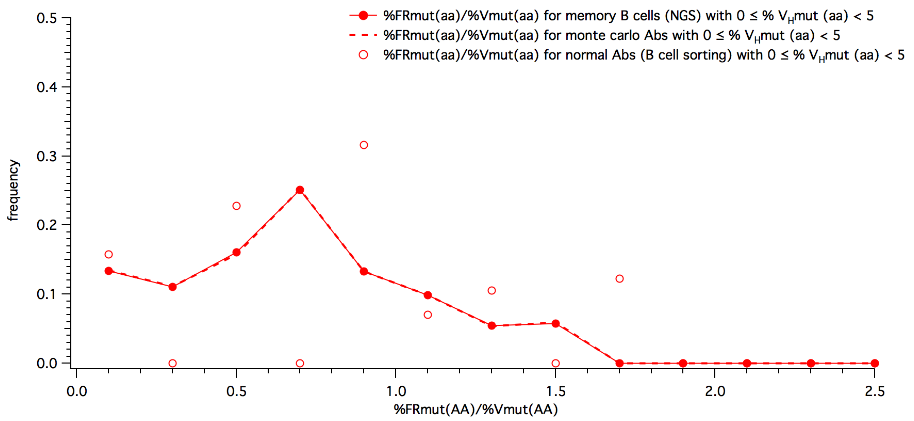

N.

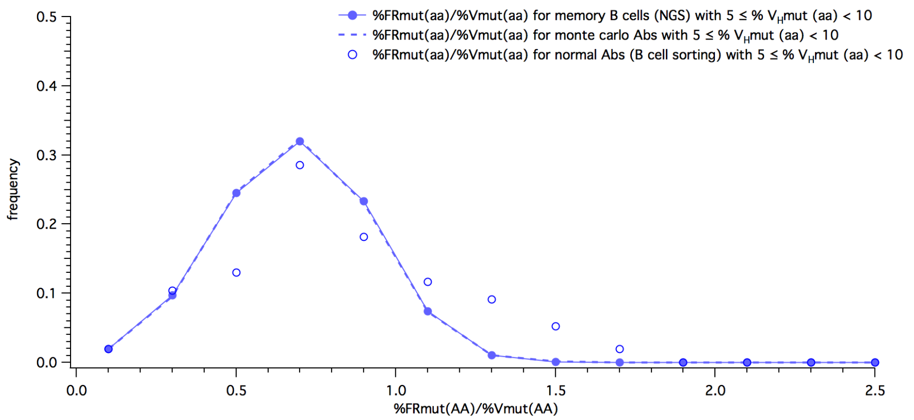

O.

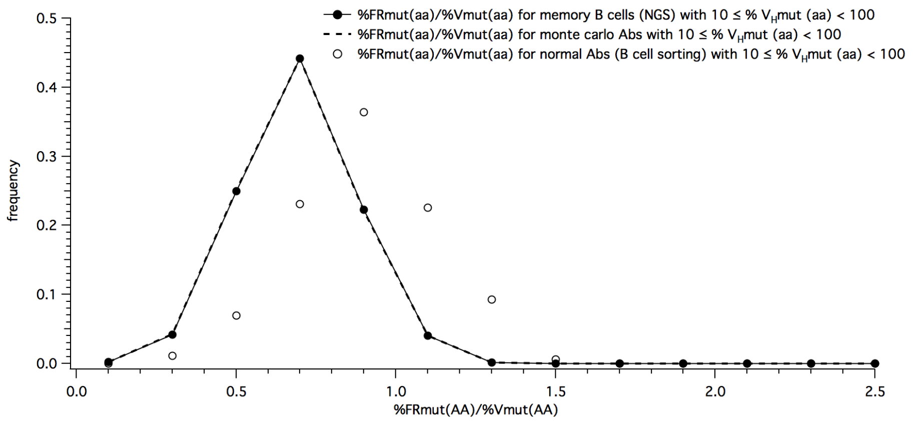

Fig. S1 (continued).

P.

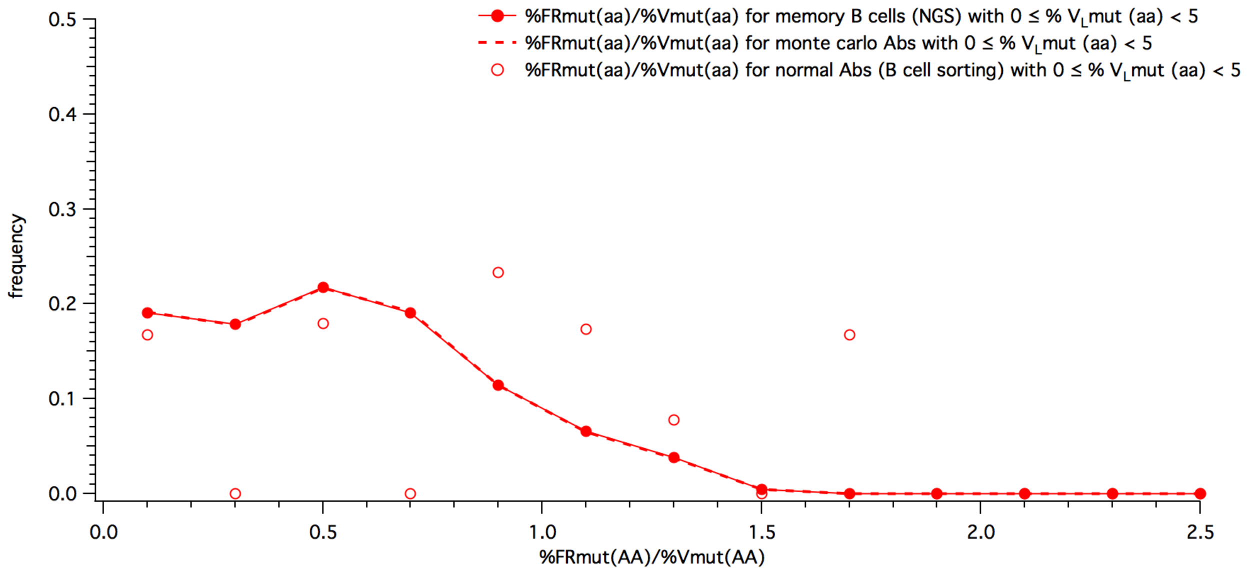

Q.

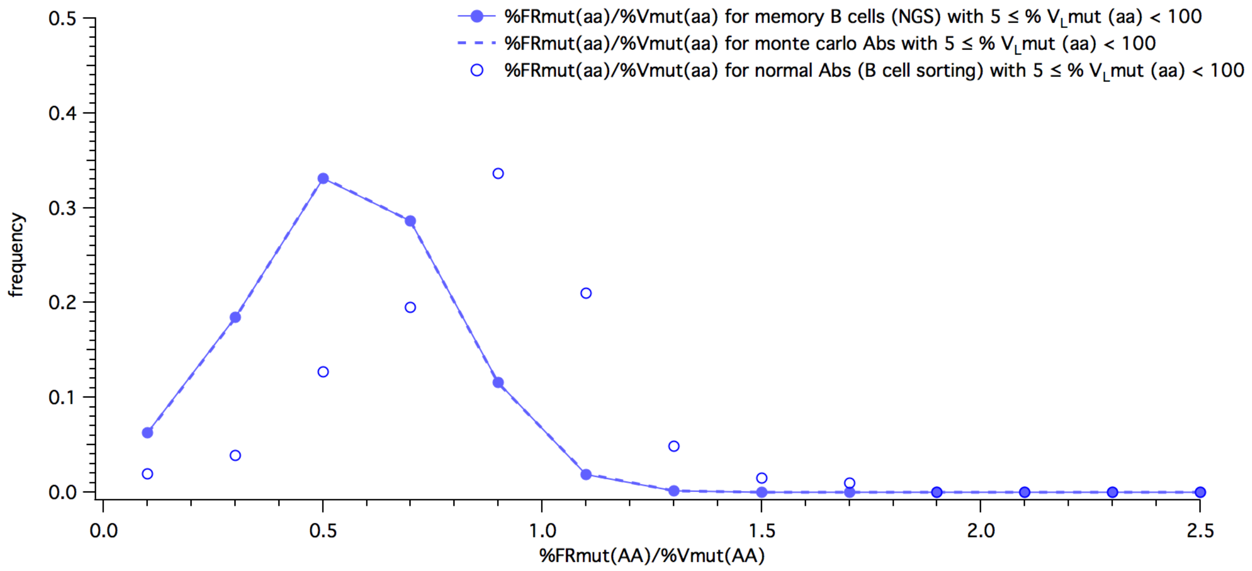

Fig. S1 (continued).

R.

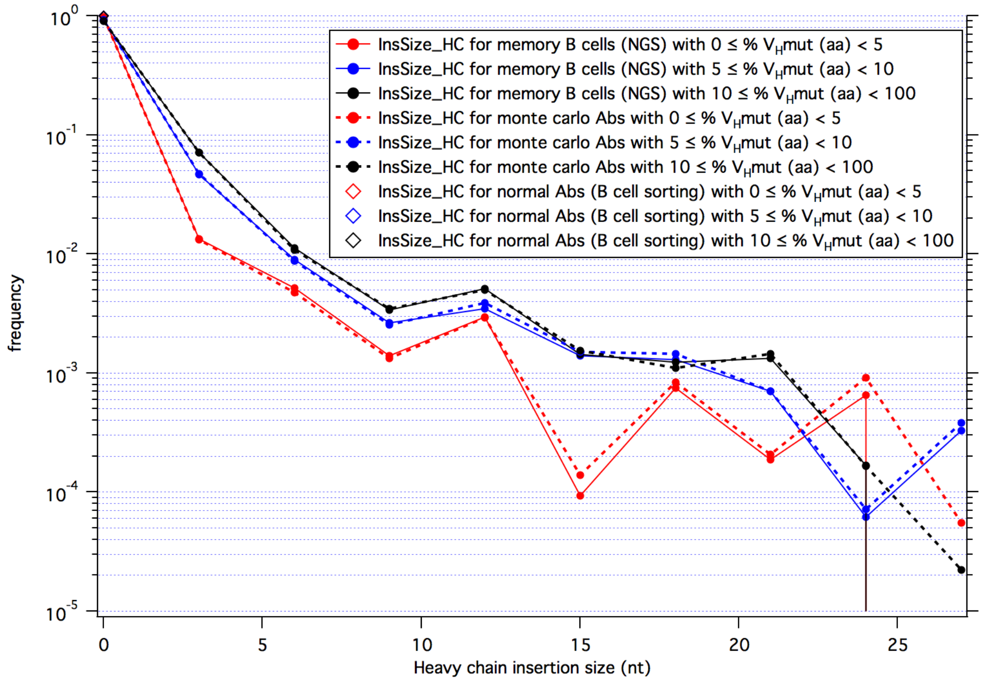

S.

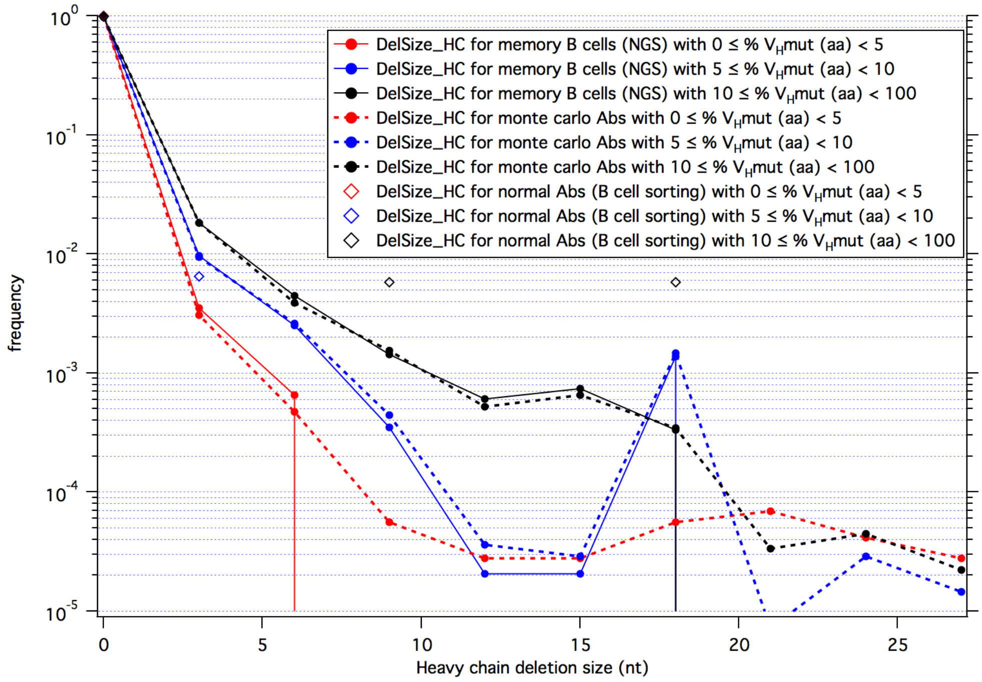

Fig. S1 (continued).

T.

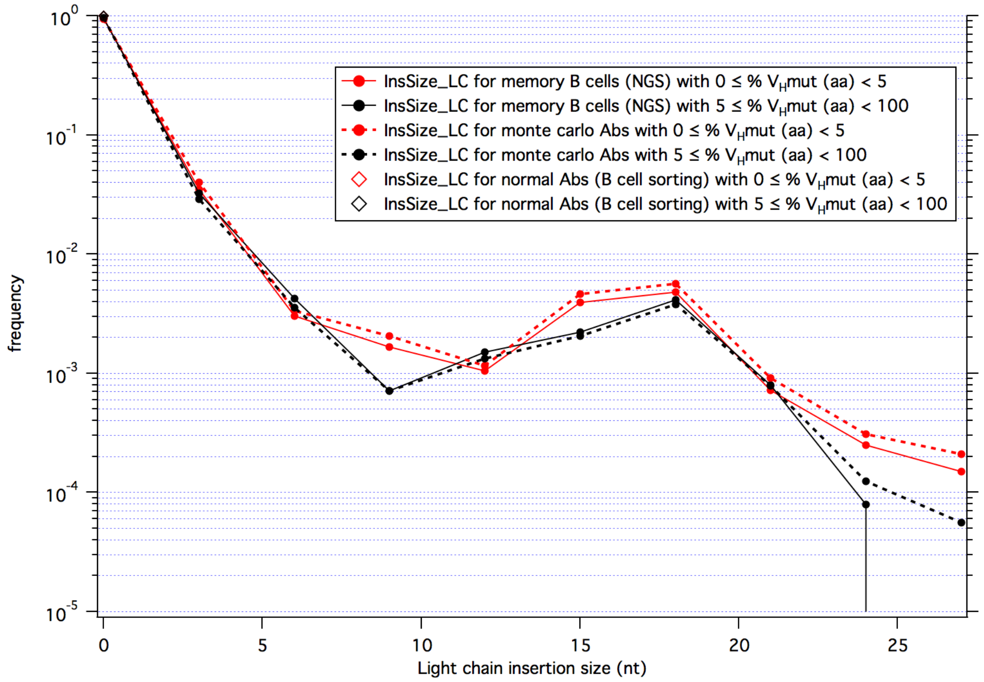

U.

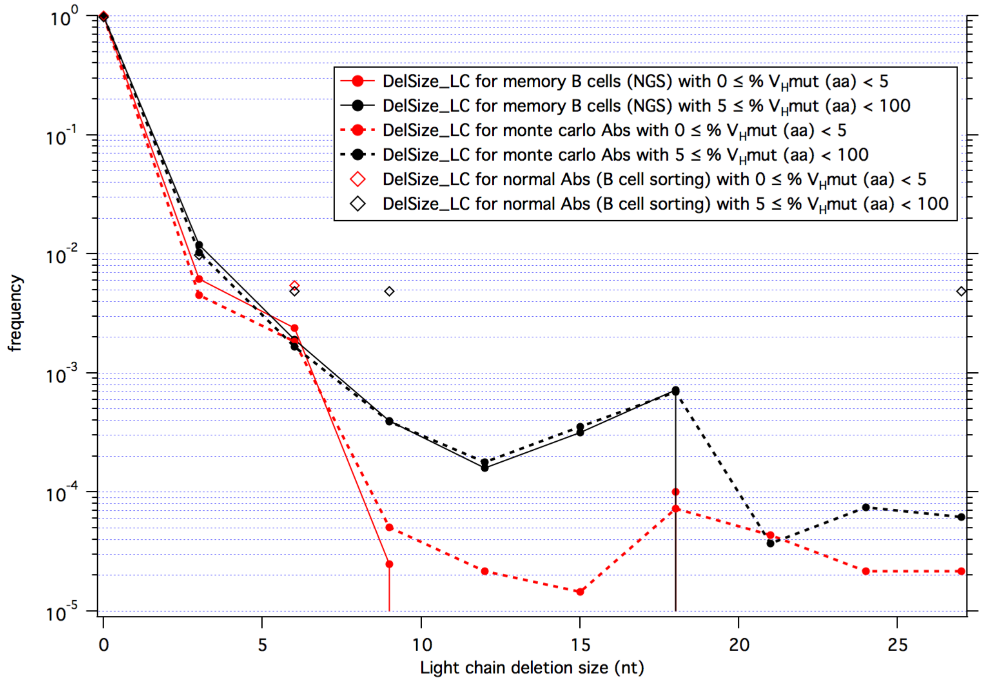

Fig. S1 (continued).

V.

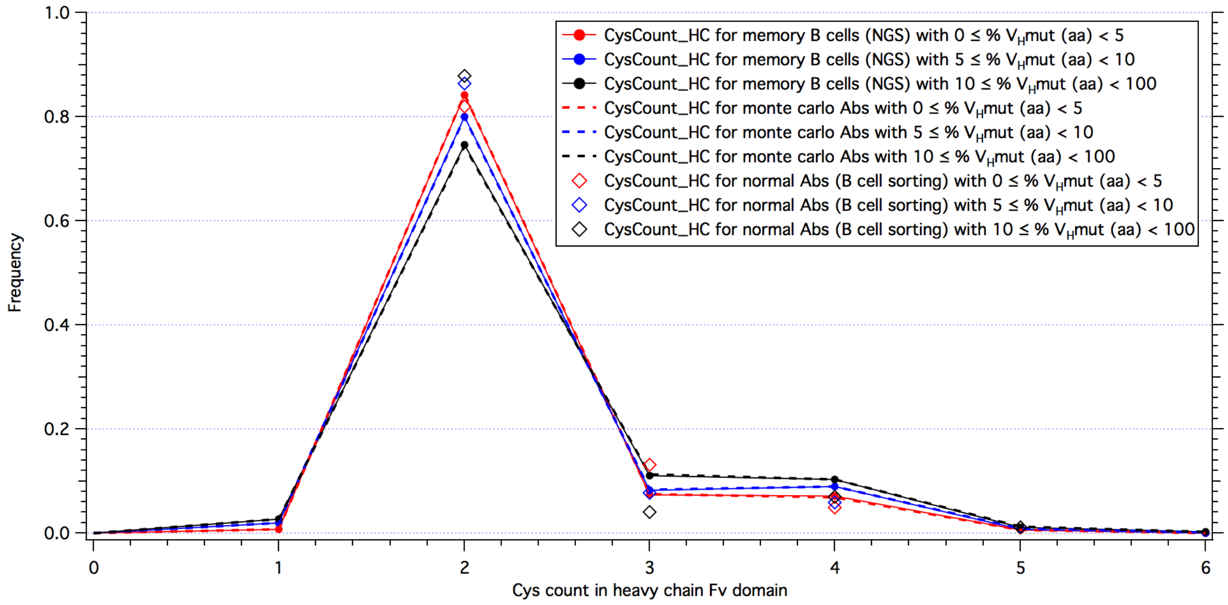

W.

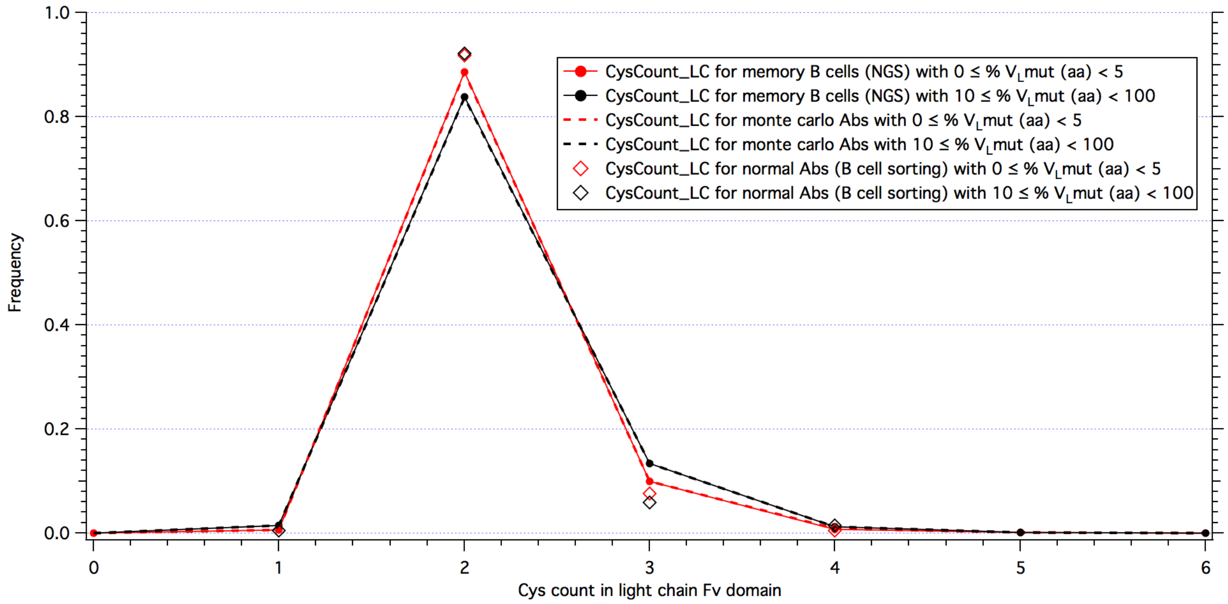

Fig. S1 (continued).
