# Supplementary figures and images for: Minimally Mutated HIV-1 Broadly Neutralizing Antibodies to Guide Reductionist Vaccine Design
Source: PLoS Pathog. 2016 Aug 25;12(8):e1005815. doi: 10.1371/journal.ppat.1005815 (PMC4999182; doi:10.1371/journal.ppat.1005815)

A.

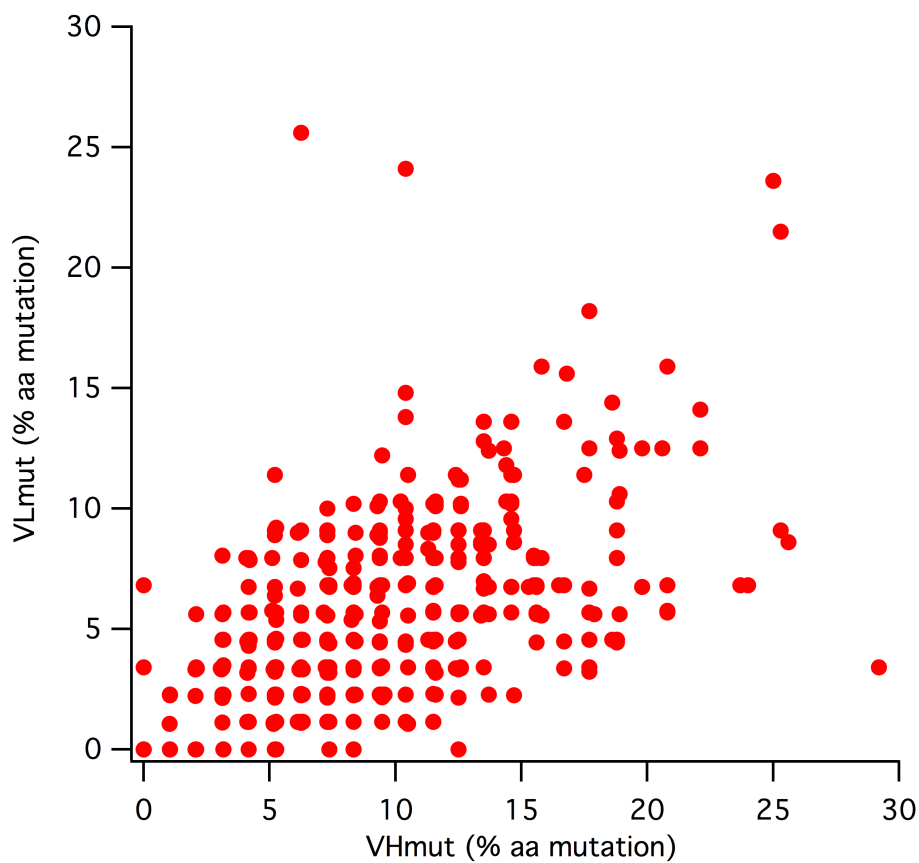

B.

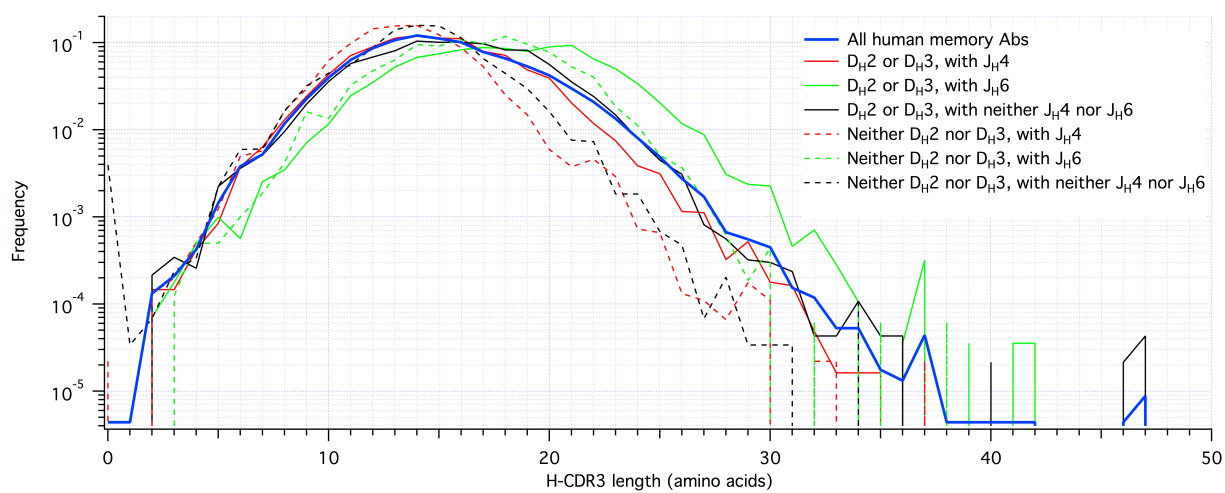

Supplement: S2 Fig — (A) Plot of the % amino acid mutation in VL versus VH for 388 normal Abs. A modest correlation can be seen, corresponding to a Pearson linear correlation coefficient of 0.49 and a Spearman rank correlation coefficient of 0.51. However, as this is not a strong correlation, the VHmut level is not a strong predictor of the VLmut level: a VHmut level of ~5.2% is accompanied by VLmut levels ranging from 0% to 11.4%, while a VHmut level of ~9.5% is accompanied by VLmut levels ranging from 1.1% to 12.2%. (B) H-CDR3 length distributions for human memory antibodies utilizing different DH and JH gene families. The blue curve shows the distribution for all 227,379 memory antibody heavy-chain sequences obtained by NGS in this study and in DeKosky et al. 2015 [29]. The red curves (solid or dashed) are for Abs using JH4, the green curves (solid or dashed) for Abs using JH6, and the black curves (solid or dashed) for Abs using JH genes other than JH4 or JH6. The solid curves (red, green or black) are for Abs using DH2 or DH3 genes, and the dashed curves (red, green, or black) for Abs using DH genes other than DH2 or DH3. In accordance with Briney et al. [78], the distribution most shifted toward longer H-CDR3 length is for Abs using DH2 or DH3 genes and using JH6, while the distribution most shifted toward shorter H-CDR3 lengths is for Abs using JH4 with neither DH2 nor DH3. For a given H-CDR3 length, the frequency correction obtained by using a distribution specifying DH and JH gene families is typically less than a factor of 10 compared to using the distribution for all human memory Abs. (PDF) [file ppat.1005815.s002.pdf]

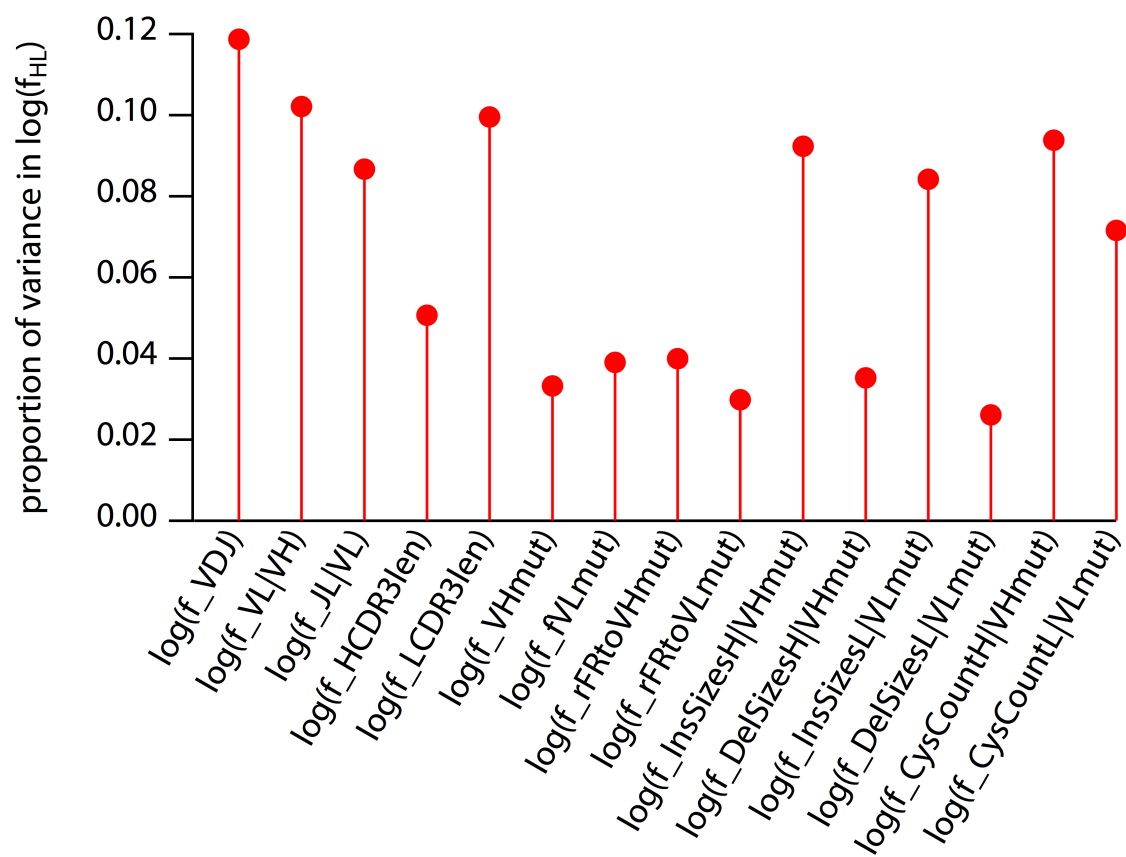

Supplement: S3 Fig — Proportion of the total variance in log(fHL) due to each individual log(f) (the log of the frequency for an individual feature) is shown, computed from antibody sequences generated by a Monte Carlo method. (PDF) [file ppat.1005815.s003.pdf]

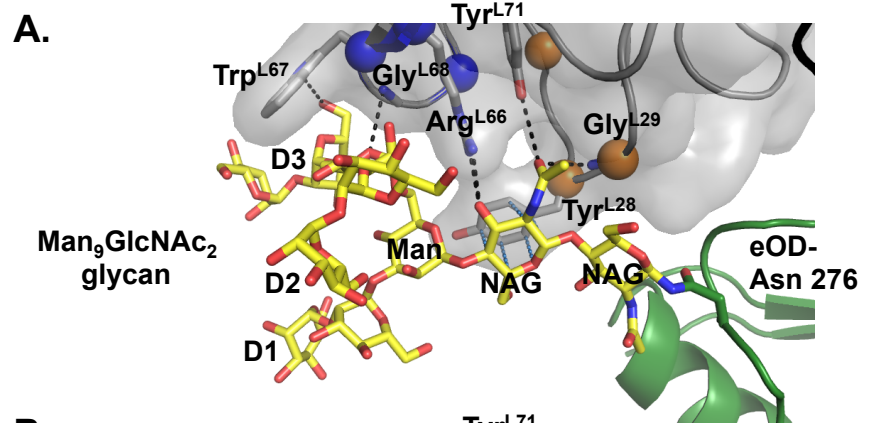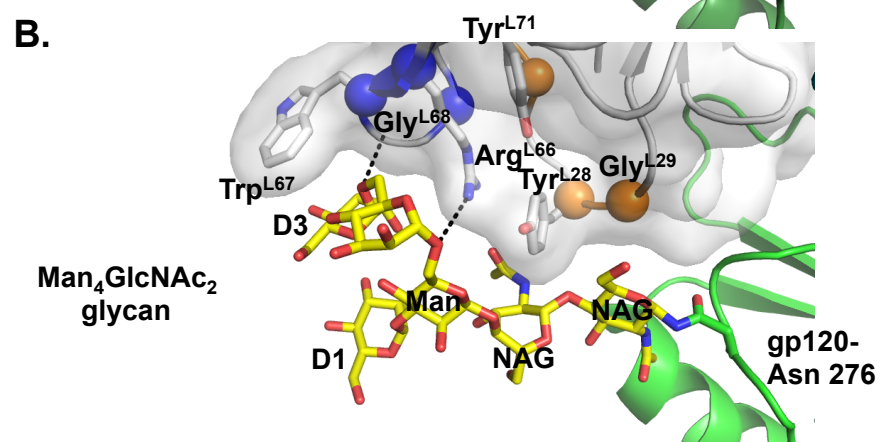

Supplement: S8 Fig — (A) The crystal structure of eOD-N276Kif containing a Man9GlcNAc2 glycan at N276 in complex with VRC01 expands our understanding of this critical interaction. (B) For comparison, the crystal structure of the gp120 core containing a Man4GlcNAc2 glycan at N276 is shown in complex with NIH45-46m2 (PDB ID: 4JKP) [47]. Rendered as in Fig 5B. (PDF) [file ppat.1005815.s008.pdf]

**A.**

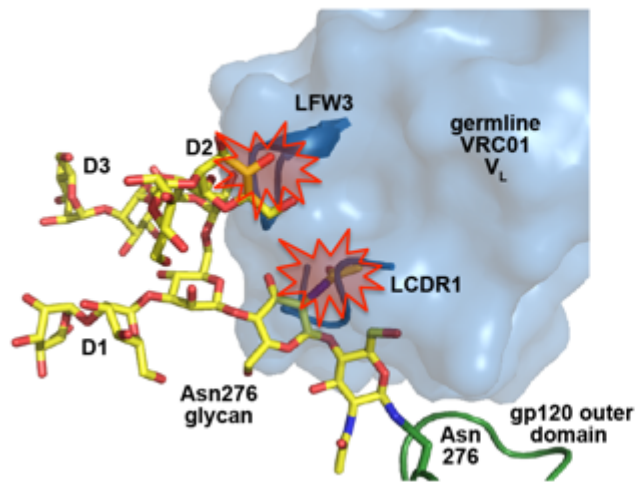

**B.**

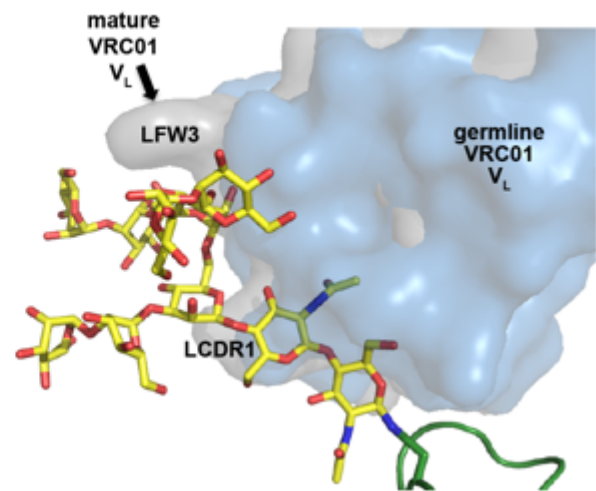

C.

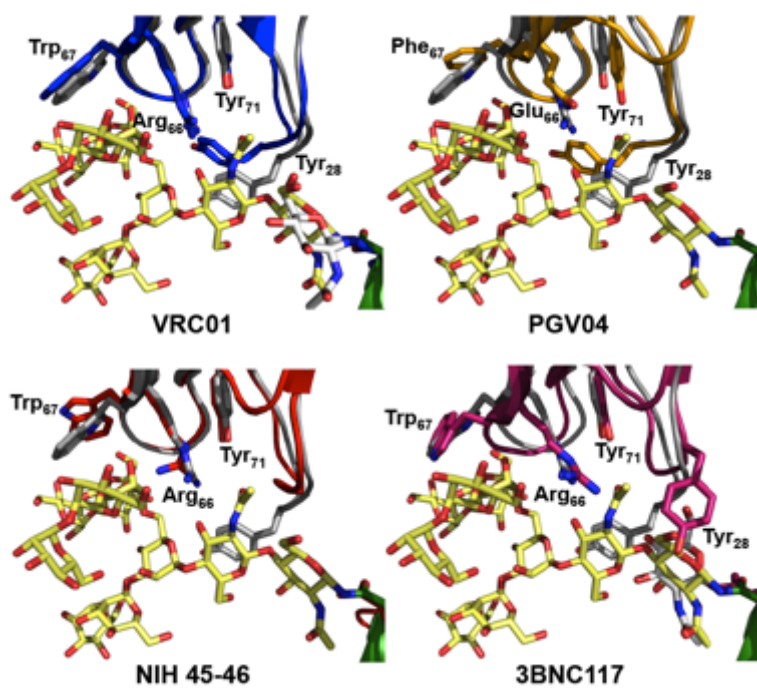

D.

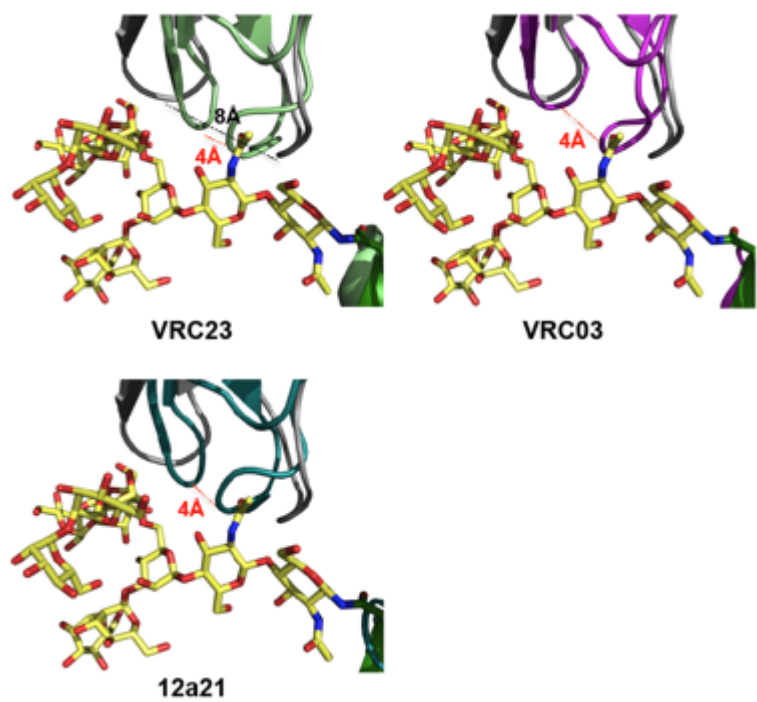

Supplement: S9 Fig — (A) Predicted clashes of germline-VRC01 with the N276 glycan. Superposition of the germline-VRC01/germline-targeting eOD-GT6 crystal structure (PDB ID: 4JPK, blue surface) on the VRC01-eOD-N276Kif crystal structure reveals potential clashes (red stars) of germline-VRC01 LFW3 and L-CDR1 loops (tubes) with the N276 glycan (yellow sticks). (B) Affinity maturation in VRC01 (gray surface) results in slight conformational changes in L-FW3 and a shorter L-CDR1, which allows interactions with the N276 glycan. The eOD main chain is colored green and shown in a tube representation. (C) Predicted interaction of various VRC01-class bnAbs with the N276 glycan. Superposition of crystal structures of core gp120 in complex with CD4bs bnAbs VRC01 (blue, PDB ID: 3GNB), PGV04 (orange, PDB ID: 4I3S), NIH45-46 (red, PDB ID: 3U7Y) and 3BNC117 (magenta, PDB ID: 4LSV) on the VRC01 (gray)-eOD-N276Kif (green) crystal structure reveals a similar mode of recognition of the N276 glycan (yellow sticks) for all these antibodies. (D) Predicted interaction of other VRC01-class bnAbs with the N276 glycan. Superposition of crystal structures of core gp120 in complex with CD4bs bnAbs VRC23 (light green, PDB ID: 4J6R), VRC03 (purple, PDB ID: 3SE8), and 12A21 (cyan, PDB ID: 4JPW) on the VRC01 (gray)-eOD-N276Kif (green) crystal structure indicates significant differences in the position of L-CDR1 and L-FW3 loops, suggesting a slightly different mode of recognition of the N276 glycan (yellow sticks) for these antibodies compared to VRC01. However, the position of these loops in the absence of N276 might be influenced by crystal packing and they might, therefore, adopt a different conformation in the liganded form. (PDF) [file ppat.1005815.s009.pdf]

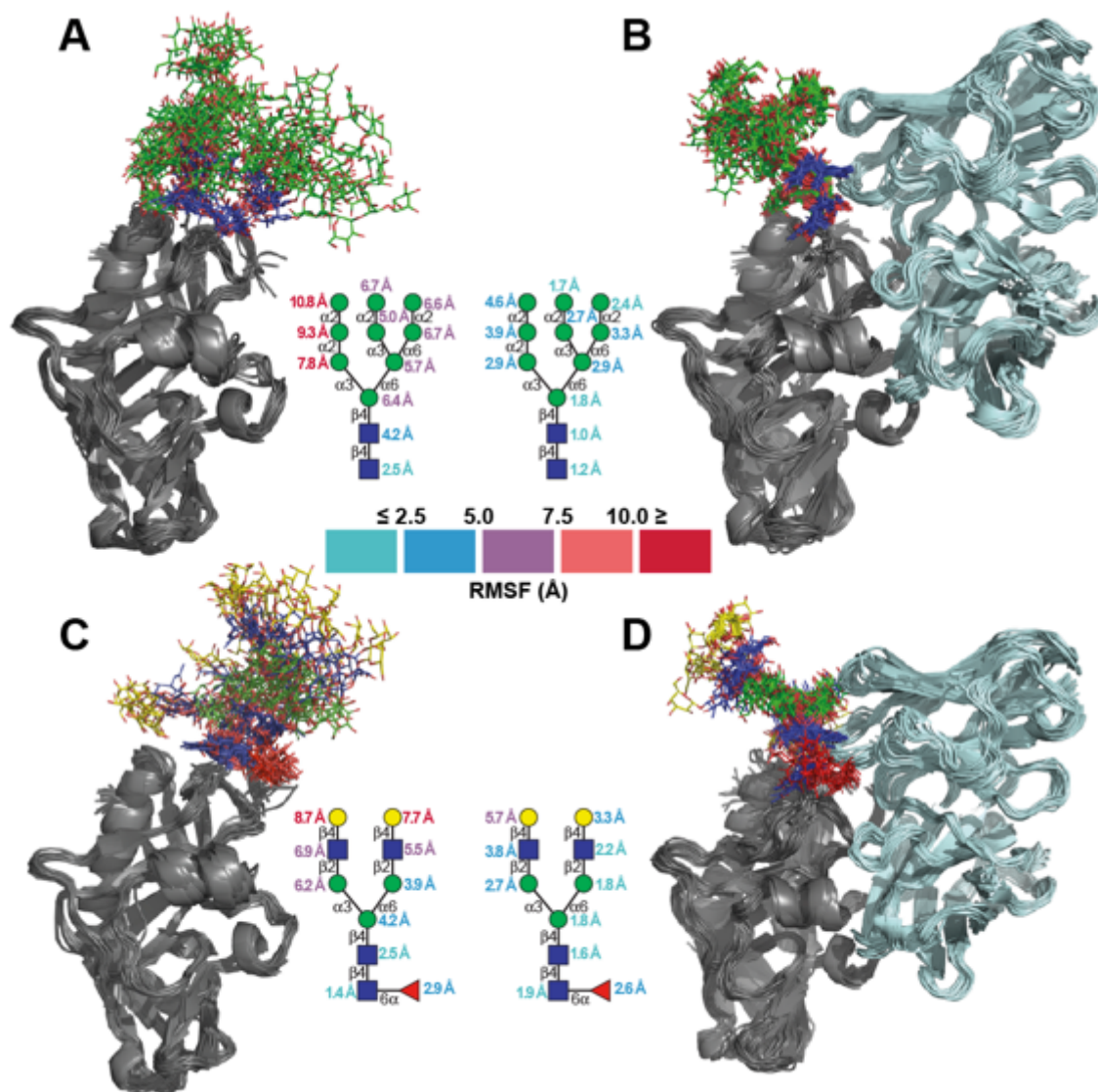

Supplement: S10 Fig — (A) Superposition of structures generated by molecular dynamics (MD) simulations of the unliganded eOD (gray) shows the area sampled by the Man9GlcNAc2 at N276. The corresponding root mean square fluctuation (RMSF in Å) of each glycan residue is described with a color code over its Consortium for Functional Glycomics (CFG) symbol representation. The high RMSF observed here shows the high flexibility of the glycans represented by a variety of conformations acquired by the Man9GlcNAc2 on the unliganded state of eOD. (B) Superposition of eOD (with Man9GlcNAc2)-VRC01 complex structures from MD clearly illustrates the restriction on the allowed conformational space of Man9GlcNAc2 when VRC01 (cyan) binds, as also reflected in the RMSF values. (C) Superposition of eOD with a core-fucosylated biantennary complex glycan [Gal β1–4 GlcNAc β1–2 Man α1–6 (Gal β1–4 GlcNAc β1–2 Manα1–3) Man β1–4GlcNAc β1–4 (Fuc α1–6) GlcNAcβ1] at N276 from MD simulations in the unliganded state shows the glycan sampling a large conformational space with high RMSF values. (D) The core-fucosylated biantennary complex glycan is restricted to a narrow conformational space upon VRC01 binding. (PDF) [file ppat.1005815.s010.pdf]
