# Supplementary material for: Minimally Mutated HIV-1 Broadly Neutralizing Antibodies to Guide Reductionist Vaccine Design
Source: PLoS Pathog. 2016 Aug 25;12(8):e1005815. doi: 10.1371/journal.ppat.1005815 (PMC4999182; doi:10.1371/journal.ppat.1005815)
Supplement: S4 Fig — (A) VRC01-class bnAbs. (B) Minimally mutated and other engineered VRC01-class bnAbs. (C) 10E8 (solid symbol in Fig 1A.) (D) Less potent variants of 10E8 with reduced mutation (open symbols in Fig 1A). (E) Potent V2Apex bnAbs (solid symbols in Fig 1A). (F) Less potent V2Apex bnAbs (open symbols in Fig 1A). (G) CH103-class bnAbs. (H) PGT121-class bnAbs. (I) Very potent high mannose patch bnAbs isolated from infected individuals (solid symbols in Fig 1A). (J) 32H3L, a very potent high mannose patch bnAb engineered to use less mutated heavy and light chains from the PGT124 lineage identified by NGS by Sok et al. [40]. (K) Less potent high mannose patch bnAbs (open symbols in Fig 1A). (L) VH1-46 bnAbs. (M) VRC16. (N) 8ANC195. (O) 35O22. (P) VRC13. (PDF) [file ppat.1005815.s004.pdf]

A.

SeqID	VRC-CH34	VRC-CH30	VRC-CH31	VRC-CH32	VRC-CH33	3BNC117
VDJ	HV1-2_HD6-19_HJ5	HV1-2_HD3-16_HJ5	HV1-2_HD1-7_HJ1	HV1-2_HD1-26_HJ4	HV1-2_HD3-10_HJ5	HV1-2_HD6-25_HJ2
fVDJ	0.000457	0.000409	8.8e-06	0.00118	0.00102	8.8e-06
VJ	KV1D-33_KJ2	KV1D-33_KJ2	KV1D-33_KJ2	KV1D-33_KJ2	KV1D-33_KJ2	KV1D-33_KJ3
fVL|VH	0.0493	0.0493	0.0493	0.0493	0.0493	0.0493
fJL|VL	0.276	0.276	0.276	0.276	0.276	0.204
VHmut	35.2	36.2	36.2	35.2	36.2	36
fVHmut	1e-05	1e-05	1e-05	1e-05	1e-05	1e-05
VLmut	21.6	20.5	22.7	23.9	25	23.8
fVLmut	0.00019	0.00019	7.61e-05	7.61e-05	1.9e-05	7.61e-05
rFRtoVmut_H	0.98	1	0.955	0.929	0.955	0.989
f_rFRtoVmut_H	0.223	0.0405	0.223	0.223	0.223	0.223
rFRtoVmut_L	1.14	1.11	1.16	1.19	1.21	1.43
f_rFRtoVmut_L	0.0187	0.0187	0.0187	0.0187	0.00119	3.49e-05
HCDR3	15.0	15.0	15.0	15.0	15.0	12.0
fHCDR3	0.112	0.112	0.112	0.112	0.112	0.0863
LCDR3	5.0	5.0	5.0	5.0	5.0	5.0
fLCDR3	0.00615	0.00615	0.00615	0.00615	0.00615	0.00615
InsCount_H	1	1	1	1	1	1
InsSizes_H	27	27	27	27	27	12
fInsSizes_H|VHmut	3.35e-05	3.35e-05	3.35e-05	3.35e-05	3.35e-05	0.0051
DelCount_H	0	0	0	0	0	0
DelSizes_H	0	0	0	0	0	0
fDelSizes_H|VHmut	0.974	0.974	0.974	0.974	0.974	0.974
InsCount_L	0	0	0	0	0	0
InsSizes_L	0	0	0	0	0	0
fInsSizes_L|VLmut	0.954	0.954	0.954	0.954	0.954	0.954
DelCount_L	0	0	0	0	0	1
DelSizes_L	0	0	0	0	0	12
fDelSizes_L|VLmut	0.985	0.985	0.985	0.985	0.985	0.000159
CysCount_H	2	2	2	2	2	2
fCysCount_H|VHmut	0.746	0.746	0.746	0.746	0.746	0.746
CysCount_L	2	2	2	2	2	2
fCysCount_L|VLmut	0.838	0.838	0.838	0.838	0.838	0.838
fHL	6.5e-25	1.06e-25	5.01e-27	6.7e-25	9.22e-27	1.32e-31

SeqID	12A12	VRC-PG19	NIH45-46	VRC02	VRC01	VRC-PG20
VDJ	HV1-2_HD2-2_HJ5	HV1-2_HD3-3_HJ1	HV1-2_HD2-8_HJ2	HV1-2_HD3-16_HJ2	HV1-2_HD5-18_HJ2	HV1-2_HD3-10_HJ1
fVDJ	0.00116	4.4e-05	4.4e-06	4.4e-05	1.76e-05	3.08e-05
VJ	KV1D-33_KJ1	LV2-14_LJ3	KV3-20_KJ2	KV3-20_KJ2	KV3-20_KJ2	LV2-14_LJ3
fVL|VH	0.0493	0.00537	0.104	0.104	0.104	0.00537
fJL|VL	0.0531	0.227	0.289	0.289	0.289	0.227
VHmut	34	30.5	40.6	39.6	41.7	37.5
fVHmut	1e-05	1e-05	1e-05	1e-05	1e-05	1e-05
VLmut	23.9	20.2	27.9	31.4	29.1	27.4
fVLmut	7.61e-05	0.00019	1.9e-05	1.9e-05	1.9e-05	1.9e-05
rFRtoVmut_H	0.961	1.07	0.806	0.873	0.916	0.97
f_rFRtoVmut_H	0.223	0.0405	0.223	0.223	0.223	0.223
rFRtoVmut_L	1.19	0.933	1.22	1.14	1.1	1.24
f_rFRtoVmut_L	0.0187	0.115	0.00119	0.0187	0.0187	0.00119
HCDR3	15.0	13.0	18.0	14.0	14.0	15.0
fHCDR3	0.112	0.107	0.0661	0.121	0.121	0.112
LCDR3	5.0	5.0	5.0	5.0	5.0	5.0
fLCDR3	0.00615	0.00122	0.00615	0.00615	0.00615	0.00122
InsCount_H	0	0	0	0	0	0
InsSizes_H	0	0	0	0	0	0
fInsSizes_H|VHmut	0.905	0.905	0.905	0.905	0.905	0.905
DelCount_H	0	0	0	0	0	0
DelSizes_H	0	0	0	0	0	0
fDelSizes_H|VHmut	0.974	0.974	0.974	0.974	0.974	0.974
InsCount_L	0	1	1	0	1	1
InsSizes_L	0	6	6	0	6	6
fInsSizes_L|VLmut	0.954	0.00422	0.00422	0.954	0.00422	0.00422
DelCount_L	0	1	1	1	1	1
DelSizes_L	0	3	6	6	6	18
fDelSizes_L|VLmut	0.985	0.0119	0.00191	0.00191	0.00191	0.000717
CysCount_H	2	2	4	4	4	2
fCysCount_H|VHmut	0.746	0.746	0.103	0.103	0.103	0.746
CysCount_L	2	2	2	2	2	2
fCysCount_L|VLmut	0.838	0.838	0.838	0.838	0.838	0.838
fHL	3.43e-21	1.74e-27	1.66e-30	1.08e-25	1.92e-28	4.33e-31

SeqID	12A21	3BNC60	VRC-PG04B	VRC-PG04	VRC18
VDJ	HV1-2_HD2-21_HJ5	HV1-2_HD6-25_HJ2	HV1-2_HD2-15_HJ2	HV1-2_HD2-8_HJ2	HV1-2_HD3-3_HJ5
fVDJ	0.000255	8.8e-06	3.08e-05	4.4e-06	0.000655
VJ	KV1D-33_KJ3	KV1D-33_KJ3	KV3-20_KJ5	KV3-20_KJ5	KV3-20_KJ4
fVL|VH	0.0493	0.0493	0.104	0.104	0.104
fJL|VL	0.204	0.204	0.0823	0.0823	0.169
VHmut	30.9	40	45.9	45.9	37.8
fVHmut	1e-05	1e-05	1e-05	1e-05	1e-05
VLmut	22.7	27.4	31.4	31.4	22.5
fVLmut	7.61e-05	1.9e-05	1.9e-05	1.9e-05	7.61e-05
rFRtoVmut_H	1	0.975	0.933	0.933	0.976
f_rFRtoVmut_H	0.0405	0.223	0.223	0.223	0.223
rFRtoVmut_L	1.08	1.38	1.14	1.14	1.01
f_rFRtoVmut_L	0.0187	0.00119	0.0187	0.0187	0.0187
HCDR3	15.0	12.0	16.0	16.0	12.0
fHCDR3	0.112	0.0863	0.101	0.101	0.0863
LCDR3	5.0	5.0	5.0	5.0	5.0
fLCDR3	0.00615	0.00615	0.00615	0.00615	0.00615
InsCount_H	0	1	2	2	1
InsSizes_H	0	12	3-3	3-3	6
fInsSizes_H|VHmut	0.905	0.0051	0.00501	0.00501	0.0112
DelCount_H	0	0	0	0	0
DelSizes_H	0	0	0	0	0
fDelSizes_H|VHmut	0.974	0.974	0.974	0.974	0.974
InsCount_L	0	0	0	0	0
InsSizes_L	0	0	0	0	0
fInsSizes_L|VLmut	0.954	0.954	0.954	0.954	0.954
DelCount_L	0	1	1	1	0
DelSizes_L	0	12	9	9	0
fDelSizes_L|VLmut	0.985	0.000159	0.000398	0.000398	0.985
CysCount_H	2	2	2	2	2
fCysCount_H|VHmut	0.746	0.746	0.746	0.746	0.746
CysCount_L	2	2	2	2	2
fCysCount_L|VLmut	0.838	0.838	0.838	0.838	0.838
fHL	5.28e-22	1.12e-30	1.51e-28	2.16e-29	1.25e-22

B.

SeqID	MinVRC01	Min12A21	5fH6fL
VDJ	HV1−2_HD2−15_HJ1	HV1−2_HD2−21_HJ5	HV1−2_HD2−15_HJ1
fVDJ	3.52e−05	0.000255	3.52e−05
VJ	KV3−11_KJ2	KV1D−33_KJ3	KV3−11_KJ2
fVL|VH	0.0296	0.0493	0.0296
fJL|VL	0.192	0.204	0.192
VHmut	14.6	17.7	19.8
fVHmut	0.0422	0.0203	0.00831
VLmut	10.5	9.09	15.1
fVLmut	0.0492	0.118	0.00748
rFRtoVmut_H	0.873	0.924	0.735
f_rFRtoVmut_H	0.223	0.223	0.441
rFRtoVmut_L	1.08	0.623	1.12
f_rFRtoVmut_L	0.0187	0.286	0.0187
HCDR3	14.0	15.0	14.0
fHCDR3	0.121	0.112	0.121
LCDR3	5.0	5.0	5.0
fLCDR3	0.00615	0.00615	0.00615
InsCount_H	0	0	0
InsSizes_H	0	0	0
fInsSizes_H|VHmut	0.905	0.905	0.905
DelCount_H	0	0	0
DelSizes_H	0	0	0
fDelSizes_H|VHmut	0.974	0.974	0.974
InsCount_L	0	0	1
InsSizes_L	0	0	6
fInsSizes_L|VLmut	0.954	0.954	0.00422
DelCount_L	1	0	1
DelSizes_L	6	0	6
fDelSizes_L|VLmut	0.00191	0.985	0.00191
CysCount_H	4	2	4
fCysCount_H|VHmut	0.103	0.746	0.103
CysCount_L	2	2	2
fCysCount_L|VLmut	0.838	0.838	0.838
fHL	1.78e−19	1.4e−13	4.67e−23

C.

SeqID	10E8
VDJ	HV3-15_HD3-3_HJ1
fVDJ	2.2e-05
VJ	LV3-19_LJ3
fVL|VH	0.0128
fJL|VL	0.791
VHmut	26.5
fVHmut	6.02e-05
VLmut	20.7
fVLmut	0.00019
rFRtoVmut_H	1.03
f_rFRtoVmut_H	0.0405
rFRtoVmut_L	0.912
f_rFRtoVmut_L	0.115
HCDR3	22.0
fHCDR3	0.0209
LCDR3	12.0
fLCDR3	0.114
InsCount_H	0
InsSizes_H	0
fInsSizes_H|VHmut	0.905
DelCount_H	0
DelSizes_H	0
fDelSizes_H|VHmut	0.974
InsCount_L	0
InsSizes_L	0
fInsSizes_L|VLmut	0.954
DelCount_L	0
DelSizes_L	0
fDelSizes_L|VLmut	0.985
CysCount_H	2
fCysCount_H|VHmut	0.746
CysCount_L	2
fCysCount_L|VLmut	0.838
fHL	1.47e-20

D.

SeqID	10E8_19fH10fL	10E8_10fH16fL	10E8_10fH10fL	10E8_2fH10fL
VDJ	HV3-15_HD3-3_HJ1	HV3-15_HD3-3_HJ1	HV3-15_HD3-3_HJ1	HV3-15_HD3-3_HJ1
fVDJ	2.2e-05	2.2e-05	2.2e-05	2.2e-05
VJ	LV3-19_LJ3	LV3-19_LJ3	LV3-19_LJ3	LV3-19_LJ3
fVL|VH	0.0128	0.0128	0.0128	0.0128
fJL|VL	0.791	0.791	0.791	0.791
VHmut	26.5	18.4	18.4	12.2
fVHmut	6.02e-05	0.00831	0.00831	0.091
VLmut	13.8	20.7	13.8	13.8
fVLmut	0.0225	0.00019	0.0225	0.0225
rFRtoVmut_H	1.03	0.792	0.792	0.297
f_rFRtoVmut_H	0.0405	0.441	0.441	0.0416
rFRtoVmut_L	0.684	0.912	0.684	0.684
f_rFRtoVmut_L	0.286	0.115	0.286	0.286
HCDR3	22.0	22.0	22.0	22.0
fHCDR3	0.0209	0.0209	0.0209	0.0209
LCDR3	12.0	12.0	12.0	12.0
fLCDR3	0.114	0.114	0.114	0.114
InsCount_H	0	0	0	0
InsSizes_H	0	0	0	0
fInsSizes_H|VHmut	0.905	0.905	0.905	0.905
DelCount_H	0	0	0	0
DelSizes_H	0	0	0	0
fDelSizes_H|VHmut	0.974	0.974	0.974	0.974
InsCount_L	0	0	0	0
InsSizes_L	0	0	0	0
fInsSizes_L|VLmut	0.954	0.954	0.954	0.954
DelCount_L	0	0	0	0
DelSizes_L	0	0	0	0
fDelSizes_L|VLmut	0.985	0.985	0.985	0.985
CysCount_H	2	2	2	2
fCysCount_H|VHmut	0.746	0.746	0.746	0.746
CysCount_L	2	2	2	2
fCysCount_L|VLmut	0.838	0.838	0.838	0.838
fHL	4.29e-18	2.2e-17	6.45e-15	6.66e-15

E.

SeqID	PGT141	PGT143	PGT145	PGT144	PGT142	PGDM1400
VDJ	HV1−8_HD4−17_HJ6	HV1−8_HD4−17_HJ6	HV1−8_HD4−17_HJ6	HV1−8_HD4−17_HJ6	HV1−8_HD4−17_HJ6	HV1−8_HD4−17_HJ6
fVDJ	0.00011	0.00011	0.00011	0.00011	0.00011	0.00011
VJ	KV2D−28_KJ1	KV2D−28_KJ1	KV2D−28_KJ1	KV2D−28_KJ1	KV2D−28_KJ1	KV2D−28_KJ1
fVL|VH	0.0228	0.0228	0.0228	0.0228	0.0228	0.0228
fJL|VL	0.239	0.239	0.239	0.239	0.239	0.239
VHmut	27.1	27.1	28.1	29.2	29.2	45.8
fVHmut	6.02e−05	6.02e−05	2.01e−05	2.01e−05	2.01e−05	1e−05
VLmut	19.4	20.4	24.7	21.5	19.4	22.6
fVLmut	0.00097	0.00019	1.9e−05	0.00019	0.00097	7.61e−05
rFRtoVmut_H	1.01	1.01	0.97	1.12	0.997	1.03
f_rFRtoVmut_H	0.0405	0.0405	0.223	0.0405	0.223	0.0405
rFRtoVmut_L	0.975	1.02	0.687	0.965	0.975	0.668
f_rFRtoVmut_L	0.115	0.0187	0.286	0.115	0.115	0.286
HCDR3	34.0	34.0	33.0	34.0	34.0	34.0
fHCDR3	5.28e−05	5.28e−05	5.28e−05	5.28e−05	5.28e−05	5.28e−05
LCDR3	9.0	9.0	9.0	9.0	9.0	9.0
fLCDR3	0.668	0.668	0.668	0.668	0.668	0.668
InsCount_H	0	0	0	0	0	0
InsSizes_H	0	0	0	0	0	0
fInsSizes_H|VHmut	0.905	0.905	0.905	0.905	0.905	0.905
DelCount_H	0	0	0	0	0	0
DelSizes_H	0	0	0	0	0	0
fDelSizes_H|VHmut	0.974	0.974	0.974	0.974	0.974	0.974
InsCount_L	0	0	0	0	0	0
InsSizes_L	0	0	0	0	0	0
fInsSizes_L|VLmut	0.954	0.954	0.954	0.954	0.954	0.954
DelCount_L	0	0	0	0	0	0
DelSizes_L	0	0	0	0	0	0
fDelSizes_L|VLmut	0.985	0.985	0.985	0.985	0.985	0.985
CysCount_H	2	2	2	2	2	2
fCysCount_H|VHmut	0.746	0.746	0.746	0.746	0.746	0.746
CysCount_L	2	2	3	2	2	2
fCysCount_L|VLmut	0.838	0.838	0.134	0.838	0.838	0.838
fHL	2.99e−21	9.49e−23	4.25e−23	1.96e−22	5.48e−21	9.7e−23

SeqID	CAP256−VRC26.08	PG16	PG9
VDJ	HV3−30_HD3−3_HJ3	HV3−33_HD3−3_HJ6	HV3−33_HD3−3_HJ6
fVDJ	0.000268	0.000418	0.000418
VJ	LV1−51_LJ1	LV2−14_LJ3	LV2−14_LJ3
fVL|VH	0.0401	0.00351	0.00351
fJL|VL	0.152	0.227	0.227
VHmut	14.7	21.9	17.4
fVHmut	0.0422	0.00321	0.0203
VLmut	10.1	17.8	10
fVLmut	0.0492	0.00278	0.0492
rFRtoVmut_H	0.864	1.08	1.05
f_rFRtoVmut_H	0.223	0.0405	0.0405
rFRtoVmut_L	0.746	1.06	0.755
f_rFRtoVmut_L	0.286	0.0187	0.286
HCDR3	39.0	30.0	30.0
fHCDR3	4.4e−06	0.000449	0.000449
LCDR3	12.0	10.0	10.0
fLCDR3	0.114	0.217	0.217
InsCount_H	0	0	0
InsSizes_H	0	0	0
fInsSizes_H|VHmut	0.905	0.905	0.905
DelCount_H	0	0	0
DelSizes_H	0	0	0
fDelSizes_H|VHmut	0.974	0.974	0.974
InsCount_L	0	0	0
InsSizes_L	0	0	0
fInsSizes_L|VLmut	0.954	0.954	0.954
DelCount_L	0	0	0
DelSizes_L	0	0	0
fDelSizes_L|VLmut	0.985	0.985	0.985
CysCount_H	4	2	2
fCysCount_H|VHmut	0.103	0.746	0.746
CysCount_L	2	2	2
fCysCount_L|VLmut	0.838	0.838	0.838
fHL	7.69e−18	1.14e−19	1.95e−16

F.

SeqID	CH02	CH03	CH04	CH01
VDJ	HV3−20_HD3−10_HJ2	HV3−20_HD3−10_HJ2	HV3−20_HD3−10_HJ2	HV3−20_HD3−10_HJ2
fVDJ	2.2e−05	2.2e−05	2.2e−05	2.2e−05
VJ	KV3−20_KJ1	KV3−20_KJ1	KV3−20_KJ1	KV3−20_KJ1
fVL|VH	0.122	0.122	0.122	0.122
fJL|VL	0.342	0.342	0.342	0.342
VHmut	22.9	22.9	24	29.2
fVHmut	0.000642	0.000642	0.000642	2.01e−05
VLmut	23.6	19.1	16.9	16.9
fVLmut	7.61e−05	0.00097	0.00278	0.00278
rFRtoVmut_H	1.19	1.35	0.911	1.18
f_rFRtoVmut_H	0.0405	0.00141	0.223	0.0405
rFRtoVmut_L	1.04	1.09	1.23	0.896
f_rFRtoVmut_L	0.0187	0.0187	0.00119	0.115
HCDR3	26.0	26.0	26.0	26.0
fHCDR3	0.00275	0.00275	0.00275	0.00275
LCDR3	9.0	9.0	9.0	9.0
fLCDR3	0.668	0.668	0.668	0.668
InsCount_H	0	0	0	0
InsSizes_H	0	0	0	0
fInsSizes_H|VHmut	0.905	0.905	0.905	0.905
DelCount_H	0	0	0	0
DelSizes_H	0	0	0	0
fDelSizes_H|VHmut	0.974	0.974	0.974	0.974
InsCount_L	0	0	0	0
InsSizes_L	0	0	0	0
fInsSizes_L|VLmut	0.954	0.954	0.954	0.954
DelCount_L	0	0	0	0
DelSizes_L	0	0	0	0
fDelSizes_L|VLmut	0.985	0.985	0.985	0.985
CysCount_H	2	2	2	2
fCysCount_H|VHmut	0.746	0.746	0.746	0.746
CysCount_L	2	2	2	2
fCysCount_L|VLmut	0.838	0.838	0.838	0.838
fHL	3.24e−20	1.43e−20	4.13e−19	2.29e−19

G.

SeqID	CH104	CH106	CH103
VDJ	HV4-59_HD3-9_HJ4	HV4-59_HD3-9_HJ4	HV4-59_HD3-9_HJ4
fVDJ	0.000717	0.000717	0.000717
VJ	LV3-1_LJ1	LV3-1_LJ1	LV3-1_LJ1
fVL|VH	0.0279	0.0279	0.0279
fJL|VL	0.25	0.25	0.25
VHmut	22.1	23.2	22.1
fVHmut	0.000642	0.000642	0.000642
VLmut	21.8	23	23.8
fVLmut	0.00019	7.61e-05	7.61e-05
rFRtoVmut_H	0.905	0.864	0.905
f_rFRtoVmut_H	0.223	0.223	0.223
rFRtoVmut_L	0.95	0.903	1.19
f_rFRtoVmut_L	0.115	0.115	0.0187
HCDR3	15.0	15.0	15.0
fHCDR3	0.112	0.112	0.112
LCDR3	10.0	10.0	10.0
fLCDR3	0.217	0.217	0.217
InsCount_H	0	0	0
InsSizes_H	0	0	0
fInsSizes_H|VHmut	0.905	0.905	0.905
DelCount_H	0	0	0
DelSizes_H	0	0	0
fDelSizes_H|VHmut	0.974	0.974	0.974
InsCount_L	0	0	0
InsSizes_L	0	0	0
fInsSizes_L|VLmut	0.954	0.954	0.954
DelCount_L	0	0	1
DelSizes_L	0	0	9
fDelSizes_L|VLmut	0.985	0.985	0.000398
CysCount_H	2	2	2
fCysCount_H|VHmut	0.746	0.746	0.746
CysCount_L	3	3	3
fCysCount_L|VLmut	0.134	0.134	0.134
fHL	3.15e-17	1.26e-17	8.23e-22

H.

SeqID	PGT151	PGT156	PGT154	PGT158	PGT155	PGT153
VDJ	HV3-30-3_HD3-3_HJ6	HV3-30-3_HD2-15_HJ6	HV3-30-3_HD3-3_HJ6	HV3-30-3_HD3-16_HJ6	HV3-30-3_HD2-15_HJ6	HV3-30_HD2-15_HJ6
fVDJ	0.000374	0.000343	0.000374	0.000163	0.000343	0.000774
VJ	KV2D-29_KJ4	KV2D-29_KJ4	KV2D-29_KJ4	KV2D-29_KJ4	KV2-29_KJ4	KV2D-29_KJ4
fVL|VH	0.00257	0.00257	0.00257	0.00257	0.000642	0.00321
fJL|VL	0.305	0.305	0.305	0.305	0.234	0.305
VHmut	28.1	30.2	26	29.5	29.2	27.1
fVHmut	2.01e-05	1e-05	6.02e-05	2.01e-05	2.01e-05	6.02e-05
VLmut	12.9	17.2	20.4	16.1	16.1	16.1
fVLmut	0.0225	0.00278	0.00019	0.00278	0.00278	0.00278
rFRtoVmut_H	0.84	0.963	0.838	0.864	0.935	0.806
f_rFRtoVmut_H	0.223	0.223	0.223	0.223	0.223	0.223
rFRtoVmut_L	1.02	0.658	0.831	0.702	0.702	0.936
f_rFRtoVmut_L	0.0187	0.286	0.115	0.286	0.286	0.115
HCDR3	28.0	28.0	28.0	28.0	28.0	28.0
fHCDR3	0.000668	0.000668	0.000668	0.000668	0.000668	0.000668
LCDR3	9.0	9.0	9.0	9.0	9.0	9.0
fLCDR3	0.668	0.668	0.668	0.668	0.668	0.668
InsCount_H	0	0	0	0	0	0
InsSizes_H	0	0	0	0	0	0
fInsSizes_H|VHmut	0.905	0.905	0.905	0.905	0.905	0.905
DelCount_H	0	0	0	0	0	0
DelSizes_H	0	0	0	0	0	0
fDelSizes_H|VHmut	0.974	0.974	0.974	0.974	0.974	0.974
InsCount_L	0	0	0	0	0	0
InsSizes_L	0	0	0	0	0	0
fInsSizes_L|VLmut	0.954	0.954	0.954	0.954	0.954	0.954
DelCount_L	0	0	0	0	0	0
DelSizes_L	0	0	0	0	0	0
fDelSizes_L|VLmut	0.985	0.985	0.985	0.985	0.985	0.985
CysCount_H	2	2	2	2	2	2
fCysCount_H|VHmut	0.746	0.746	0.746	0.746	0.746	0.746
CysCount_L	2	2	2	2	2	2
fCysCount_L|VLmut	0.838	0.838	0.838	0.838	0.838	0.838
fHL	1.27e-19	1.1e-19	2e-20	1.05e-19	4.24e-20	7.55e-19

SeqID	PGT157	PGT152
VDJ	HV3-30-3_HD2-21_HJ6	HV3-30-3_HD3-3_HJ6
fVDJ	0.000158	0.000374
VJ	KV2D-29_KJ4	KV2D-29_KJ4
fVL|VH	0.00257	0.00257
fJL|VL	0.305	0.305
VHmut	27.4	29.2
fVHmut	6.02e-05	2.01e-05
VLmut	14	11.8
fVLmut	0.0225	0.0492
rFRtoVmut_H	0.864	0.873
f_rFRtoVmut_H	0.223	0.223
rFRtoVmut_L	0.81	0.957
f_rFRtoVmut_L	0.115	0.115
HCDR3	28.0	28.0
fHCDR3	0.000668	0.000668
LCDR3	9.0	9.0
fLCDR3	0.668	0.668
InsCount_H	0	0
InsSizes_H	0	0
fInsSizes_H|VHmut	0.905	0.905
DelCount_H	0	0
DelSizes_H	0	0
fDelSizes_H|VHmut	0.974	0.974
InsCount_L	0	0
InsSizes_L	0	0
fInsSizes_L|VLmut	0.954	0.954
DelCount_L	0	0
DelSizes_L	0	0
fDelSizes_L|VLmut	0.985	0.985
CysCount_H	2	2
fCysCount_H|VHmut	0.746	0.746
CysCount_L	2	2
fCysCount_L|VLmut	0.838	0.838
fHL	9.98e-19	1.72e-18

I.

SeqID	PGT128	PGT122	PGT123	PGT121	PGT126	PGT127
VDJ	HV4-39_HD3-3_HJ5	HV4-59_HD3-3_HJ6	HV4-59_HD3-10_HJ6	HV4-59_HD3-3_HJ6	HV4-39_HD3-3_HJ5	HV4-39_HD1-7_HJ5
fVDJ	0.00127	0.000844	0.00134	0.000844	0.00127	0.000295
VJ	LV2-8_LJ3	LV3-21_LJ3	LV3-21_LJ3	LV3-21_LJ3	LV2-8_LJ3	LV2-8_LJ3
fVL|VH	0.0187	0.0275	0.0275	0.0275	0.0187	0.0187
fJL|VL	0.439	0.716	0.716	0.716	0.439	0.439
VHmut	31.1	26.3	29.5	25.3	24.3	25.2
fVHmut	1e-05	6.02e-05	2.01e-05	0.000431	0.000431	0.000431
VLmut	11.8	28	36.6	31.2	12.9	10.6
fVLmut	0.0492	1.9e-05	1.9e-05	1.9e-05	0.0225	0.0492
rFRtoVmut_H	0.761	0.967	0.987	1.01	0.749	0.72
f_rFRtoVmut_H	0.441	0.223	0.223	0.0405	0.441	0.441
rFRtoVmut_L	1.12	0.828	0.781	0.743	1.02	1.07
f_rFRtoVmut_L	0.0187	0.115	0.286	0.286	0.0187	0.0187
HCDR3	21.0	26.0	26.0	26.0	21.0	21.0
fHCDR3	0.0298	0.00275	0.00275	0.00275	0.0298	0.0298
LCDR3	10.0	12.0	12.0	12.0	10.0	10.0
fLCDR3	0.217	0.114	0.114	0.114	0.217	0.217
InsCount_H	1	0	0	0	1	1
InsSizes_H	18	0	0	0	18	18
fInsSizes_H|VHmut	0.00123	0.905	0.905	0.905	0.00123	0.00123
DelCount_H	0	0	0	0	0	0
DelSizes_H	0	0	0	0	0	0
fDelSizes_H|VHmut	0.974	0.974	0.974	0.974	0.974	0.974
InsCount_L	0	1	1	1	0	0
InsSizes_L	0	9	9	9	0	0
fInsSizes_L|VLmut	0.954	0.000716	0.000716	0.000716	0.954	0.954
DelCount_L	1	1	1	1	1	1
DelSizes_L	15	21	21	21	15	15
fDelSizes_L|VLmut	0.000319	3.49e-05	3.49e-05	3.49e-05	0.000319	0.000319
CysCount_H	4	2	2	2	4	4
fCysCount_H|VHmut	0.103	0.746	0.746	0.746	0.103	0.103
CysCount_L	2	2	2	2	2	2
fCysCount_L|VLmut	0.838	0.838	0.838	0.838	0.838	0.838
fHL	8.64e-24	2.11e-27	2.77e-27	6.84e-27	1.7e-22	8.61e-23

SeqID	PGT125	PGT131	PGT130
VDJ	HV4-39_HD3-10_HJ5	HV4-39_HD3-9_HJ5	HV4-39_HD3-10_HJ5
fVDJ	0.00192	0.000334	0.00192
VJ	LV2-8_LJ3	LV2-8_LJ3	LV2-8_LJ3
fVL|VH	0.0187	0.0187	0.0187
fJL|VL	0.439	0.439	0.439
VHmut	29.1	29.9	29.9
fVHmut	2.01e-05	2.01e-05	2.01e-05
VLmut	21.2	22.2	20
fVLmut	0.00019	7.61e-05	0.00019
rFRtoVmut_H	0.749	1.03	0.973
f_rFRtoVmut_H	0.441	0.0405	0.223
rFRtoVmut_L	1.16	0.934	1.13
f_rFRtoVmut_L	0.0187	0.115	0.0187
HCDR3	21.0	21.0	21.0
fHCDR3	0.0298	0.0298	0.0298
LCDR3	10.0	10.0	10.0
fLCDR3	0.217	0.217	0.217
InsCount_H	1	0	0
InsSizes_H	18	0	0
fInsSizes_H|VHmut	0.00123	0.905	0.905
DelCount_H	0	0	0
DelSizes_H	0	0	0
fDelSizes_H|VHmut	0.974	0.974	0.974
InsCount_L	0	0	0
InsSizes_L	0	0	0
fInsSizes_L|VLmut	0.954	0.954	0.954
DelCount_L	1	0	0
DelSizes_L	15	0	0
fDelSizes_L|VLmut	0.000319	0.985	0.985
CysCount_H	4	2	2
fCysCount_H|VHmut	0.103	0.746	0.746
CysCount_L	2	2	2
fCysCount_L|VLmut	0.838	0.838	0.838
fHL	1.01e-25	6.58e-20	8.4e-19

J.

SeqID	PGT124_32H3L
VDJ	HV4-59_HD3-3_HJ6
fVDJ	0.000844
VJ	LV3-21_LJ3
fVL|VH	0.0275
fJL|VL	0.716
VHmut	16.8
fVHmut	0.0203
VLmut	20
fVLmut	0.00019
rFRtoVmut_H	0.864
f_rFRtoVmut_H	0.223
rFRtoVmut_L	0.357
f_rFRtoVmut_L	0.184
HCDR3	26.0
fHCDR3	0.00275
LCDR3	12.0
fLCDR3	0.114
InsCount_H	0
InsSizes_H	0
fInsSizes_H|VHmut	0.905
DelCount_H	0
DelSizes_H	0
fDelSizes_H|VHmut	0.974
InsCount_L	1
InsSizes_L	9
fInsSizes_L|VLmut	0.000716
DelCount_L	0
DelSizes_L	0
fDelSizes_L|VLmut	0.985
CysCount_H	2
fCysCount_H|VHmut	0.746
CysCount_L	2
fCysCount_L|VLmut	0.838
fHL	3.21e-19

K.

SeqID	PGT135	PGT137	PGT136
VDJ	HV4-39_HD2-21_HJ5	HV4-39_HD2-15_HJ5	HV4-39_HD2-15_HJ5
fVDJ	0.000435	0.000602	0.000602
VJ	KV3-15_KJ1	KV3-15_KJ1	KV3-15_KJ1
fVL|VH	0.0415	0.0415	0.0415
fJL|VL	0.335	0.335	0.335
VHmut	29.4	32.7	29.1
fVHmut	2.01e-05	1e-05	2.01e-05
VLmut	29.5	19.3	21.6
fVLmut	1.9e-05	0.00097	0.00019
rFRtoVmut_H	1.05	1.06	1.06
f_rFRtoVmut_H	0.0405	0.0405	0.0405
rFRtoVmut_L	1.02	0.977	1.14
f_rFRtoVmut_L	0.0187	0.115	0.0187
HCDR3	20.0	20.0	20.0
fHCDR3	0.042	0.042	0.042
LCDR3	9.0	9.0	9.0
fLCDR3	0.668	0.668	0.668
InsCount_H	1	1	2
InsSizes_H	15	15	15-3
fInsSizes_H|VHmut	0.00143	0.00143	0.000101
DelCount_H	0	0	0
DelSizes_H	0	0	0
fDelSizes_H|VHmut	0.974	0.974	0.974
InsCount_L	0	0	0
InsSizes_L	0	0	0
fInsSizes_L|VLmut	0.954	0.954	0.954
DelCount_L	0	0	0
DelSizes_L	0	0	0
fDelSizes_L|VLmut	0.985	0.985	0.985
CysCount_H	2	2	2
fCysCount_H|VHmut	0.746	0.746	0.746
CysCount_L	2	2	2
fCysCount_L|VLmut	0.838	0.838	0.838
fHL	4.03e-23	8.79e-21	3.95e-23

L.

SeqID	1NC9	8ANC131	8ANC134	1B2530
VDJ	HV1−46_HD5−12_HJ4	HV1−46_HD3−16_HJ6	HV1−46_HD3−16_HJ6	HV1−46_HD3−10_HJ5
fVDJ	0.000532	0.000119	0.000119	0.000264
VJ	LV1−47_LJ3	KV3−NL5_KJ3	KV3−NL1_KJ3	LV1−47_LJ3
fVL|VH	0.0281	0.00121	0.000302	0.0281
fJL|VL	0.779	0.14	5.55e−06	0.779
VHmut	37.5	41.1	40	40.6
fVHmut	1e−05	1e−05	1e−05	1e−05
VLmut	25	26.4	27.6	22.5
fVLmut	1.9e−05	1.9e−05	1.9e−05	7.61e−05
rFRtoVmut_H	1.07	0.947	0.972	1.03
f_rFRtoVmut_H	0.0405	0.223	0.223	0.0405
rFRtoVmut_L	0.679	1.28	1.23	0.924
f_rFRtoVmut_L	0.286	0.00119	0.00119	0.115
HCDR3	21.0	18.0	18.0	18.0
fHCDR3	0.0298	0.0661	0.0661	0.0661
LCDR3	11.0	9.0	9.0	11.0
fLCDR3	0.483	0.668	0.668	0.483
InsCount_H	0	0	0	0
InsSizes_H	0	0	0	0
fInsSizes_H|VHmut	0.905	0.905	0.905	0.905
DelCount_H	0	1	1	0
DelSizes_H	0	3	3	0
fDelSizes_H|VHmut	0.974	0.0183	0.0183	0.974
InsCount_L	0	0	0	0
InsSizes_L	0	0	0	0
fInsSizes_L|VLmut	0.954	0.954	0.954	0.954
DelCount_L	0	1	1	0
DelSizes_L	0	6	3	0
fDelSizes_L|VLmut	0.985	0.00191	0.0119	0.985
CysCount_H	2	2	2	2
fCysCount_H|VHmut	0.746	0.746	0.746	0.746
CysCount_L	2	2	2	2
fCysCount_L|VLmut	0.838	0.838	0.838	0.838
fHL	1.93e−19	8.45e−28	5.21e−32	3.41e−19

M.

SeqID	VRC16
VDJ	HV3−23D_HD3−22_HJ4
fVDJ	0.00395
VJ	KV1D−39_KJ1
fVL|VH	0.0982
fJL|VL	0.318
VHmut	27.1
fVHmut	6.02e−05
VLmut	28.4
fVLmut	1.9e−05
rFRtoVmut_H	1.07
f_rFRtoVmut_H	0.0405
rFRtoVmut_L	1.26
f_rFRtoVmut_L	0.00119
HCDR3	22.0
fHCDR3	0.0209
LCDR3	9.0
fLCDR3	0.668
InsCount_H	0
InsSizes_H	0
fInsSizes_H|VHmut	0.905
DelCount_H	0
DelSizes_H	0
fDelSizes_H|VHmut	0.974
InsCount_L	0
InsSizes_L	0
fInsSizes_L|VLmut	0.954
DelCount_L	0
DelSizes_L	0
fDelSizes_L|VLmut	0.985
CysCount_H	2
fCysCount_H|VHmut	0.746
CysCount_L	2
fCysCount_L|VLmut	0.838
fHL	4.93e−20

N.

SeqID	8ANC195
VDJ	HV1-69D_HD3-3_HJ4
fVDJ	0.000726
VJ	KV1-5_KJ1
fVL|VH	0.0759
fJL|VL	0.493
VHmut	43.4
fVHmut	1e-05
VLmut	29.2
fVLmut	1.9e-05
rFRtoVmut_H	1.13
f_rFRtoVmut_H	0.0405
rFRtoVmut_L	0.969
f_rFRtoVmut_L	0.115
HCDR3	22.0
fHCDR3	0.0209
LCDR3	9.0
fLCDR3	0.668
InsCount_H	2
InsSizes_H	3-12
fInsSizes_H|VHmut	0.000361
DelCount_H	1
DelSizes_H	6
fDelSizes_H|VHmut	0.00445
InsCount_L	1
InsSizes_L	3
fInsSizes_L|VLmut	0.0325
DelCount_L	0
DelSizes_L	0
fDelSizes_L|VLmut	0.985
CysCount_H	2
fCysCount_H|VHmut	0.746
CysCount_L	2
fCysCount_L|VLmut	0.838
fHL	1.09e-26

O.

SeqID	35022
VDJ	HV1-18_HD3-10_HJ5
fVDJ	0.000888
VJ	LV2-23_LJ1
fVL|VH	0.0292
fJL|VL	0.425
VHmut	33.7
fVHmut	1e-05
VLmut	38.9
fVLmut	1.9e-05
rFRtoVmut_H	1.04
f_rFRtoVmut_H	0.0405
rFRtoVmut_L	1.02
f_rFRtoVmut_L	0.0187
HCDR3	16.0
fHCDR3	0.101
LCDR3	10.0
fLCDR3	0.217
InsCount_H	1
InsSizes_H	24
fInsSizes_H|VHmut	0.000167
DelCount_H	0
DelSizes_H	0
fDelSizes_H|VHmut	0.974
InsCount_L	0
InsSizes_L	0
fInsSizes_L|VLmut	0.954
DelCount_L	0
DelSizes_L	0
fDelSizes_L|VLmut	0.985
CysCount_H	2
fCysCount_H|VHmut	0.746
CysCount_L	6
fCysCount_L|VLmut	6.98e-05
fHL	2.77e-28

P.

SeqID	VRC13
VDJ	HV1−69D_HD3−9_HJ2
fVDJ	1.76e−05
VJ	LV2−14_LJ1
fVL|VH	0.00307
fJL|VL	0.772
VHmut	47.5
fVHmut	1e−05
VLmut	26.2
fVLmut	1.9e−05
rFRtoVmut_H	0.956
f_rFRtoVmut_H	0.223
rFRtoVmut_L	1.37
f_rFRtoVmut_L	0.00119
HCDR3	23.0
fHCDR3	0.0134
LCDR3	6.0
fLCDR3	0.00141
InsCount_H	1
InsSizes_H	15
fInsSizes_H|VHmut	0.00143
DelCount_H	0
DelSizes_H	0
fDelSizes_H|VHmut	0.974
InsCount_L	0
InsSizes_L	0
fInsSizes_L|VLmut	0.954
DelCount_L	1
DelSizes_L	18
fDelSizes_L|VLmut	0.000717
CysCount_H	2
fCysCount_H|VHmut	0.746
CysCount_L	2
fCysCount_L|VLmut	0.838
fHL	2.38e−32
