# Supplementary material for: Minimally Mutated HIV-1 Broadly Neutralizing Antibodies to Guide Reductionist Vaccine Design
Source: PLoS Pathog. 2016 Aug 25;12(8):e1005815. doi: 10.1371/journal.ppat.1005815 (PMC4999182; doi:10.1371/journal.ppat.1005815)
Supplement: S5 Fig — The features and their associated frequencies are given for all the antibodies analyzed in Fig 1. Descriptions of each feature and frequency are shown in the leftmost columns. Values for each antibody are shown as a column. (PDF) [file ppat.1005815.s005.pdf]

SeqID	CR9114-HC	C05-Homo-sapiens	FI6-HC	2G1-Homo-sapiens	1F1-Homo-sapiens	5J8-Homo-sapiens
VDJ	HV1-69_HD1-1_HJ6	HV3-23D_HD6-13_HJ3	HV3-30_HD3-9_HJ4	HV1-69D_HD1-26_HJ5	HV3-30-3_HD3-22_HJ5	HV4-38-2_HD3-3_HJ4
fVDJ	7.48e-05	0.000572	0.000726	4.84e-05	6.16e-05	0.000171
VJ	LV1-44_LJ7	KV1D-33_KJ4	KV4-1_KJ1	KV1D-33_KJ5	LV1-44_LJ3	LV3-21_LJ3
fVL|VH	0.0363	0.044	0.0535	0.0719	0.0353	0.0257
fJL|VL	0.00879	0.336	0.298	0.131	0.707	0.716
VHmut	17.7	23.2	7.29	13.7	5.26	10.5
fVHmut	0.0203	0.000642	0.197	0.091	0.15	0.134
VLmut	12.5	13.6	4.35	7.95	5.62	8.05
fVLmut	0.0225	0.0225	0.252	0.211	0.252	0.118
rFRtoVmut_H	1.03	0.785	0.748	0.664	0.691	1.04
f_rFRtoVmut_H	0.0405	0.441	0.32	0.441	0.32	0.0405
rFRtoVmut_L	0.453	0.969	0.868	0.949	0.336	0.704
f_rFRtoVmut_L	0.331	0.115	0.114	0.115	0.184	0.286
HCDR3	14.0	26.0	22.0	13.0	19.0	17.0
fHCDR3	0.121	0.00275	0.0209	0.107	0.0535	0.0783
LCDR3	11.0	9.0	9.0	9.0	12.0	11.0
fLCDR3	0.483	0.668	0.668	0.668	0.114	0.483
InsCount_H	0	0	0	0	0	0
InsSizes_H	0	0	0	0	0	0
fInsSizes_H|VHmut	0.905	0.905	0.934	0.905	0.934	0.905
DelCount_H	0	0	0	0	0	0
DelSizes_H	0	0	0	0	0	0
fDelSizes_H|VHmut	0.974	0.974	0.986	0.974	0.986	0.974
InsCount_L	0	0	0	0	0	0
InsSizes_L	0	0	0	0	0	0
fInsSizes_L|VLmut	0.954	0.954	0.949	0.954	0.954	0.954
DelCount_L	0	0	1	0	0	0
DelSizes_L	0	0	6.0	0	0	0
fDelSizes_L|VLmut	0.985	0.985	0.0024	0.985	0.985	0.985
CysCount_H	2	2	2	2	2	2
fCysCount_H|VHmut	0.746	0.746	0.8	0.746	0.8	0.746
CysCount_L	2	2	2	2	2	2
fCysCount_L|VLmut	0.838	0.838	0.886	0.838	0.838	0.838
fHL	4.43e-15	5.92e-15	4.36e-13	1.66e-11	1.21e-11	1.14e-11

SeqID	CR6261-HC-Patent-WO20101	8M2-Homo-sapiens
VDJ	HV1-69D_HD2-2_HJ6	HV1-69D_HD3-16_HJ4
fVDJ	0.000418	0.000369
VJ	LV1-51_LJ3	KV3-15_KJ1
fVL|VH	0.0341	0.0599
fJL|VL	0.83	0.335
VHmut	13.7	17.9
fVHmut	0.091	0.0203
VLmut	2.25	7.95
fVLmut	0.228	0.211
rFRtoVmut_H	1.2	0.711
f_rFRtoVmut_H	0.0405	0.441
rFRtoVmut_L	0.84	0.237
f_rFRtoVmut_L	0.114	0.184
HCDR3	14.0	16.0
fHCDR3	0.121	0.101
LCDR3	12.0	10.0
fLCDR3	0.114	0.174
InsCount_H	0	0
InsSizes_H	0	0
fInsSizes_H|VHmut	0.905	0.905
DelCount_H	0	0
DelSizes_H	0	0
fDelSizes_H|VHmut	0.974	0.974
InsCount_L	0	0
InsSizes_L	0	0
fInsSizes_L|VLmut	0.949	0.954
DelCount_L	0	0
DelSizes_L	0	0
fDelSizes_L|VLmut	0.991	0.985
CysCount_H	2	2
fCysCount_H|VHmut	0.746	0.746
CysCount_L	2	2
fCysCount_L|VLmut	0.886	0.838
fHL	8.58e-12	2.36e-11
