# Supplementary material for: Minimally Mutated HIV-1 Broadly Neutralizing Antibodies to Guide Reductionist Vaccine Design
Source: PLoS Pathog. 2016 Aug 25;12(8):e1005815. doi: 10.1371/journal.ppat.1005815 (PMC4999182; doi:10.1371/journal.ppat.1005815)
Supplement: S6 Fig — (A) Cardiolipin binding assay. The indicated antibodies were assayed for cardiolipin binding at the concentrations shown, with 4E10 as a positive control and Humira as a negative control. (B) Single antigen binding assay. The indicated antibodies were assayed for binding to eight nuclear and cellular auto-antigens at an antibody concentration of 50 μg/mL. Values within 20% of the cut-off calibrator score as equivocal. (C) Assessment of the effect of introducing Phe54 into VRC01 (by the G54F mutation) or removing Phe54 from MinVRC01 (by the F54S mutation), by the Hep2 cell binding assay. (D) Assessment of the effects of Phe54 as in (C), by the Polyspecificity reagent (PSR) binding assay; positive controls, 4E10 and 2F5; negative controls, Humira, 10E8, and PG9. (PDF) [file ppat.1005815.s006.pdf]

A.

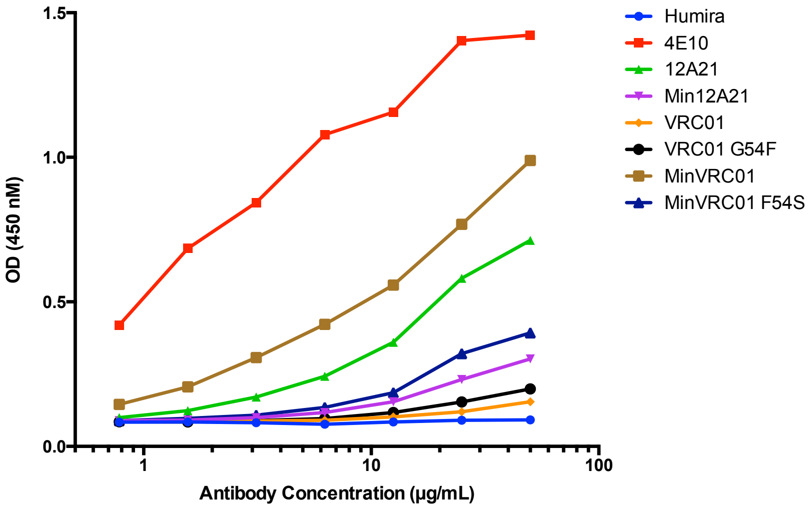

B.

Antibody	µg/ml	Autoantigen							
		U1-RNP	snRNP/Sm	Sm	SS-A	SS-B	Scl-70	Cenp-B	Jo-1
12A21	50	12.41	14.85	14.98	15.13	13.22	13.31	14.16	10.43
Min12A21	50	5.55	5.36	4.56	5.79	4.06	4.29	3.88	5.54
VRC01	50	2.94	2.40	2.54	2.79	2.16	2.35	1.77	2.47
MinVRC01	50	19.54	20.62	20.50	23.84	15.14	16.12	15.20	11.15
VRC01 G54F	50	3.98	3.52	3.96	4.72	3.26	4.48	3.19	3.78
MinVRC01 F54S	50	12.02	9.42	11.05	12.86	8.44	11.00	9.09	8.89
10E8	50	4.10	3.52	4.89	6.13	3.49	5.25	3.74	5.65
Humira	50	1.69	2.41	2.24	1.54	2.44	2.58	3.46	2.44
Cut-off Calibrator		4.08	4.14	4.29	4.04	3.34	3.60	3.14	3.15
Negative Control		1.21	1.22	1.23	1.33	1.29	1.32	1.27	1.20

C.

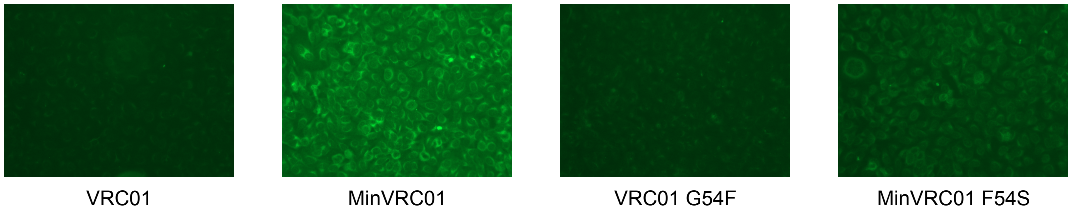
VRC01
MinVRC01
VRC01 G54F
MinVRC01 F54S

D.

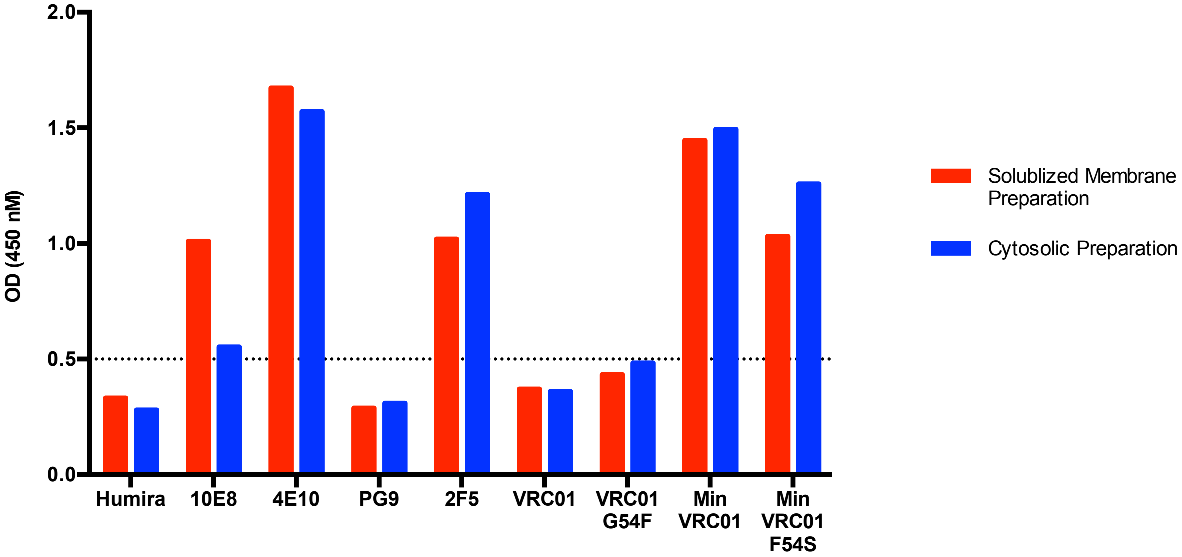
2.0
1.5
1.0
0.5
0.0
OD (450 nM)
Humira
10E8
4E10
PG9
2F5
VRC01
VRC01 G54F
Min VRC01
Min VRC01 F54S
Solublized Membrane Preparation
Cytosolic Preparation
