# Supplementary material for: Minimally Mutated HIV-1 Broadly Neutralizing Antibodies to Guide Reductionist Vaccine Design
Source: PLoS Pathog. 2016 Aug 25;12(8):e1005815. doi: 10.1371/journal.ppat.1005815 (PMC4999182; doi:10.1371/journal.ppat.1005815)
Supplement: S7 Fig — (A) Alignment of VRC01-class antibody heavy chains and the inferred germline precursor VH1-2*02, which highlight the regions that align to MinVRC01 patches. (B) Alignment of VRC01-class antibody light chains and their inferred germline precursors, which highlight the regions that align to MinVRC01 patches. Patches in (A) and (B) are colored according to the scheme used in Fig 2. (PDF) [file ppat.1005815.s007.pdf]

A.

```
              --------------FR1--------------_____CDR1____-------FR2----________CDR2______
IGHV1-02*02   QVQLVQSGAEVKKPGASVKVSCK-ASGYTFTGY--------YMHWVRQAPGQGLEWMGWINPNSGGTNY-AQKFQG
MinVRC01      .......................-........C--------TLN................K.RF.AV..-.R....
Min12A21      ..................R....-.......N.--------IL..W..............K.VF.AV..-.RQ...
5fH           .......................-....E.IDC--------TLN...............LK.RG.AV..-.RPL..
12A12         SQH.....TQ........RI..Q-....S..D.--------VL..W..............K.VY.AR..-.RR...
12A21         SQH.....TQ........R...Q-.......N.--------IL..W............L.K.VF.AV..-.RQ...
3BNC117       ....L....A.T......R...E-....NIRD.--------FI..W........Q.V.....KT.QP.N-PRQ...
3BNC60        ..H.S....A.T......R...E-....KISDH--------FI..W........Q.V.....KT.QP.N-PRQ...
NIH45-46      ..R.S...GQM....E.MRL..R-....E.LNC--------PIN.I.L...RRP.....LK.RG.AV..-.R....
PGV04         ........SG........R...WTSEDIFERTE--------LI.............I..VKTVT.AV.FGSPD.RQ
PGV19         E.R...............R...A-.......DF--------DI..L.....R.......VR.LG..VS.-.RQ...
PGV20         ..H.M...T.M.......R.T.Q-T.....SD.--------FI..L..V..R.F.....M..QW.QV..-.RT...
VRC-CH30      .........A.R......T....F.EDDDYSPHWVNPAPEH.I.FL......Q...LA.M..TN.AV..-.WQLH.
VRC-CH31      .........A.R......T....F.EDDDYSP.WVNPAPEHFI.FL......Q...LA.M..TN.AV..-.WYLN.
VRC01         ........GQM....E.MRI..R-....E.IDC--------TLN.I.L...KRP.....LK.RG.AV..-.RPL..
VRC03         .........VI.T..S...I..R-....N.RD.--------SI....LI.DK.F..I...K.LW.AVS.-.RQL..
VRC06         E....E..PVMR...S.M.I..A-T...N.RDF--------SI....FNRRY.F..I...K.MW.AV..-.RQL..
VRC23         ...F..............R...E-....S..D.--------VLQ.I......RP......K.ER.AVS.-.PQ...
```

```
              ---------------------FR3---------------_______CDR3_____----FR4----
IGHV1-02*02   RVTMTR----DTSIS---TAYMELSRLRSDDTAVYYCAR
MinVRC01      ......----.VYSD---.....................GKNCDYN----WDFQHWGQGTLVTVSS
Min12A21      ......----.IYRE---I....................DESGD-D—LKWHLHPWGQGTQVIVSP
5fH           ......----.VYS.---.....................GKNCDYN----WDFQHWGQGTLVTVSS
12A12         .INFD.----.IYRE---I.F.D..G.......L.F...DGSGD-D--TSWHLDPWGQGTLVIVSA
12A21         .IQL..----.IYRE---I.FLD..G.............DESGD-D--LKWHLHPWGQGTQVIVSP
3BNC117       ..SL..HASW.FDTF---SF..D.KA.........F...QRSDY------WDFDVWGSGTQVTVSS
3BNC60        ..SL..QASW.FDTY---SF..D.KAV......I.F...QRSDF------WDFDVWGSGTQVTVSS
NIH45-46      ......----.VYSD---..FL..RS.T.......F.T.GKYCTARDYYNWDFEHWGRGAPVTVSS
PGV04         ..SL..----.RDLF---..H.DIRG.TQG...T.F...QKFYTGG--QGWYFDLWGRGTLIVVSS
PGV19         ......----.FY.D---..F.DFRN.KM....L.F...MGAAR-----EWDFQYWGQGTRVLVSS
PGV20         ......----.VYRE---V..LD.RS.TFA.....F...RMRSQ-D--REWDFQHWGQGTRIIVSS
VRC-CH30      .L.A..----.G.MT---..FL.VRS.............AQKRGR---SEWAYAHWGQGTPVLVSS
VRC-CH31      ...A..----.R.MT---..FL.VKS.............AQKRGR---SEWAYAHWGQGTPVVVSS
VRC01         ......----.VYSD---..FL..RS.TV......F.T.GKNCD----YNWDFEHWGRGTPVIVSS
VRC03         ..S...QLSQ.PDDPDWGV....F.G.TPA...E.F.V.RGSCDYC--GDFPWQYWGQGTVVVVSS
VRC06         ..S.S.LFSQ.LYYPDRG...L.F.G.T.A...D.F.V.RGSSCPH-CGDFHFEHWGQGTAVVVSA
VRC23         .L.L..----.LYTE---....HFKN.......I.....GVRRDA----SWWLQFWGQGTLVTVSS
```

B.

```
             ----------FR1----------____CDR1____------FR2------__CDR2_
IGKV1-33*01  DIQMTQSPSSLSASVGDRVTITCQASQDISNY-LNWYQQKPGKAPKLLIYDASNLET
12A12        ........................G.G.GSS-.Q.............VHG....HR
12A21        .....................N...G.G.GSS-.....K...R.....VHG....QR
Min12A21     ........................G.G.GSS-.......................
3BNC60       .............R...T.......N----G.-......RR..........G.K..R
3BNC117      .................T.......----NG.-......RR..........G.K..R
VRC-CH30     ..............L...........RG.GKD-..............VS...I..G
VRC-CH31     ..............L...........RG.GKD-.......A.......VS...T..G
IGKV3-11*01  EIVLTQSPATLSLSPGERATLSCRASQSVSSY-LAWYQQKPGQAPRLLIYDASNRAT
6fL          ........................T..YG.---..............V..SG.T..A
NIH45-46     .................T.II...T...G.---......R.......V..SG.T..A
VRC01        ........G........T.II...T..YG.---......R.......V..SG.T..A
MinVRC01     ........................T..YG.---.................G.....
IGKV3-15*01  EIVMTQSPATLSVSPGERATLSCRASQSVSSN-LAWYQQKPGQAPRLLIYGASTRAT
VRC23        ........V.V...R.GT.........G.G.D-V....H....T............S
IGKV3-20*01  EIVLTQSPGTLSLSPGERATLSCRASQSVSSSYLAWYQQKPGQAPRLLIYGASSRAT
PGV04        .................T.S...T.ASYGH---MT...K....P.K...FAT.K..S
VRC03        .........I.......T...F.K...GGNA--MT...KRR..V......DT.R..S
VRC06        ........A..................GGN.--.N...KRR..T......DT.R..S
IGLV2-14*01  QSALTQ-PASVSGSPGQSITISCTGTSSDVGGYNYVSWYQQHPGKAPKLMIYEVSNR
PGV19        ......-.................-A...FR.FSS......V..R....L.FS.NR.
PGV20        ......-.P...........L....A.T------S.A....YAD...R.IVFDGNK.

             ---------------FR3--------------_CDR3----FR4-----
IGKV1-33*01  GVPSRFSGSGSGTDFTFTISSLQPEDIATYYCQQYDNLP
12A12        ..........FH.T.SL...G..RD.F...F.AVLEFFGPGTKVEIK
12A21        ..........FH.T..L.......D.V...F.AVFQWFGPGTKVDIK
Min12A21     ..........FH.T..................AVFQWFGPGTKVDIK
3BNC60       ...A....RRW.QEYNL..NN.....V...F..V.EFIVPGTRLDLK
3BNC117      ........RRW.QEYNL..NN.........F..V.EFVVPGTRLDLK
VRC-CH30     ..........FHQN.SL.........V...F....ETFGQGTKVDIK
VRC-CH31     ..........FHQN.SL......A..V...F....ETFGQGTKVDIK
IGKV3-11*01  GIPARFSGSGSGTDFTLTISSLEPEDFAVYYCQQRSNWP
6fL          .........R..P.Y...................YEFFGPGTKVQVDIK
NIH45-46     ...D.....RW.A.YN.S..N..SG..G......YEFFGQGTKVQVDIK
VRC01        ...D.....RW.P.YN....N..SG..G......YEFFGQGTKVQVDIK
MinVRC01     .........RW.P.....................YEFFGQGTKLEIK
IGKV3-15*01  GIPARFSGSGSGTEFTLTISSLQSEDFAVYYCQQYNNWP
VRC23        .V.E......FHVD...S..G..P..V.I......ETFGQGTKVEIK
IGKV3-20*01  GIPDRFSGSGSGTDFTLTISRLEPEDFAVYYCQQYGSSP
PGV04        .........QF.KQY....T.M......R.....LEFFGQGTRLEIR
VRC03        .V....V........F...NK.DR..........FEFFGLGSELEVH
VRC06        D..EK.V........S...TKVG...........FEFFGLGTTLEIN
IGLV2-14*01  PSGVSNRFSGSKSGNTASLTISGLQAEDEADYYCSSYTSSSTL
PGV19        ...I.H...................I......H.NA.EFFGGGTKVFVLGQ
PGV20        ..DI.S.....Q..G..........S....Y.H.NAFEFFGGGTKLTVLSQ
```
