# Supplementary material for: Minimally Mutated HIV-1 Broadly Neutralizing Antibodies to Guide Reductionist Vaccine Design
Source: PLoS Pathog. 2016 Aug 25;12(8):e1005815. doi: 10.1371/journal.ppat.1005815 (PMC4999182; doi:10.1371/journal.ppat.1005815)
Supplement: S1 Table — VRC01, MinVRC01, 12A21, and Min12A21 were tested on a cross-clade 80-virus panel. Values are neutralization IC50 in μg/ml and are colored according to the legend. (PDF) [file ppat.1005815.s011.pdf]

Table S1. Minimally mutated variants of VRC01 (MinVRC01) and 12A21 (Min12A21) show high retention of neutralization breadth and potency compared to affinity-mature antibodies. VRC01, MinVRC01, 12A21, and Min12A21 were tested on a cross-clade 80-virus panel. Presented values are neutralization IC_{50} in μg/ml and colored according to the listed legend.

VIRUS	CLADE	VRC01	MinVRC01	12A21	Min12A21
Q23.17	A	0.083	0.128	0.036	0.105
Q461.e2	A	0.425	0.642	0.077	0.267
Q769.d22	A	0.052	0.015	0.021	0.060
0330.v4.c3	A	0.069	0.089	0.023	0.054
0260.v5.c36	A	0.252	0.319	0.141	0.480
191084 B7-19	A	0.054	0.030	0.033	4.049
QH0692.42	B	1.46	1.33	0.799	1.857
SC422661.8	B	0.097	0.014	0.338	> 50
PVO.4	B	0.648	0.328	0.262	0.886
TRO.11	B	0.329	0.332	0.070	0.203
AC10.0.29	B	1.98	1.45	0.710	4.51
RHPA4259.7	B	0.043	0.016	0.005	> 50
REJO4541.67	B	0.034	0.020	0.097	> 50
WITO4160.33	B	0.129	0.516	0.025	0.280
CAAN5342.A2	B	2.42	0.750	0.083	1.24
WEAU_d15_410_5017	B (T/F)	0.125	0.012	0.269	> 50
1006_11_C3_1601	B (T/F)	0.189	3.43	0.010	2.76
1054_07_TC4_1499	B (T/F)	0.001	0.934	0.049	0.256
1056_10_TA11_1826	B (T/F)	1.71	0.764	0.466	> 50
6240_08_TA5_4622	B (T/F)	1.54	1.57	0.034	> 50
6244_13_B5_4576	B (T/F)	0.160	0.055	0.480	0.987
62357_14_D3_4589	B (T/F)	0.475	0.169	0.070	0.267
SC05_8C11_2344	B (T/F)	0.543	0.253	0.120	0.778
Du156.12	C	0.105	0.012	0.011	2.24
Du422.1	C	>50	>50	0.665	> 50
ZM214M.PL15	C	0.748	0.686	0.049	0.197
ZM249M.PL1	C	0.041	0.025	0.028	> 50
ZM53M.PB12	C	1.07	0.780	0.131	0.447
ZM109F.PB4	C	0.084	0.044	0.094	> 50
CAP45.2.00.G3	C	1.20	>50	0.026	> 50
CAP210.2.00.E8	C	>50	>50	> 50	> 50
HIV-001428-2.42	C	0.017	0.002	0.010	2.22
HIV-16055-2.3	C	0.051	0.030	0.014	0.071
HIV-16845-2.22	C	3.08	1.11	2.99	> 50
Ce1176_A3	C (T/F)	1.06	0.750	0.211	0.739
Ce2010_F5	C (T/F)	0.231	0.101	0.061	0.184
Ce2060_G9	C (T/F)	0.230	0.123	0.053	0.163
Ce703010054_2A2	C (T/F)	0.431	0.292	0.170	> 50
246F C1G	C (T/F)	1.55	>50	0.059	> 50
249M B10	C (T/F)	0.079	0.024	0.053	0.178
ZM247v1(Rev-)	C (T/F)	0.279	>50	0.022	> 50
7030102001E5(Rev-)	C (T/F)	0.541	0.098	0.008	3.69
1394C9G1(Rev-)	C (T/F)	0.329	0.811	0.008	0.019
Ce704809221_1B3	C (T/F)	0.464	0.073	0.047	0.119
X2131_C1_B5	G	0.477	0.263	0.925	> 50
P1981_C5_3	G	0.189	0.091	0.086	3.10
X1632_S2_B10	G	0.055	0.022	0.109	> 50
3016.v5.c45	D	0.066	0.460	0.010	0.099
231965.c01	D	0.133	0.042	> 50	> 50

Neutralization IC_{50} (µg/mL)
50
10.0
1.00
0.100
0.010
0.001

Table S1 (Continued)

VIRUS	CLADE	VRC01	MinVRC01	12A21	Min12A21
3301.v1.c24	AC	0.080	0.019	0.011	> 50
6041.v3.c23	AC	0.009	0.250	0.002	0.007
6540.v4.c1	AC	>50	>50	0.022	1.10
6545.v4.c1	AC	>50	>50	> 50	> 50
CNE19	BC	0.191	0.026	0.023	> 50
CNE21	BC	0.398	3.78	0.027	1.30
CNE17	BC	1.42	0.543	2.379	> 50
CNE30	BC	0.827	0.915	0.170	0.438
CNE52	BC	0.084	0.022	0.026	0.120
CNE53	BC	0.121	0.024	0.028	0.047
CNE58	BC	0.186	0.634	0.032	50
6480.v4.c25	CD	0.030	0.040	0.023	0.069
6952.v1.c20	CD	0.031	0.047	> 50	> 50
6811.v7.c18	CD	0.122	0.039	0.035	0.169
89-F1_2_25	CD	>50	>50	> 50	> 50
0815.v3.c3	ACD	0.012	0.005	0.013	0.044
T257-31	CRF02_AG	0.706	0.599	0.041	0.034
928-28	CRF02_AG	0.173	0.103	0.161	0.476
T250-4	CRF02_AG	>50	>50	> 50	> 50
T251-18	CRF02_AG	1.97	1.17	1.03	> 50
T278-50	CRF02_AG	>50	>50	> 50	> 50
T255-34	CRF02_AG	0.260	0.303	0.048	0.132
211-9	CRF02_AG	11.0	>50	0.048	7.44
C1080.c03	CRF01_AE	1.48	>50	0.036	0.120
R2184.c04	CRF01_AE	0.061	0.038	0.034	1.49
R3265.c06	CRF01_AE	0.326	1.09	0.084	0.180
C3347.c11	CRF01_AE	0.069	0.015	0.010	0.021
BJOX009000.02.4	CRF01_AE	1.34	1.89	> 50	> 50
BJOX015000.11.5	CRF01_AE (T/F)	0.081	0.566	0.199	0.870
BJOX025000.01.1	CRF01_AE (T/F)	0.601	1.06	0.051	0.144
BJOX028000.10.3	CRF01_AE (T/F)	>50	>50	0.042	0.017

Neutralization IC_{50} (μg/mL)
50
10.0
1.00
0.100
0.010
0.001
