# Supplementary material for: Minimally Mutated HIV-1 Broadly Neutralizing Antibodies to Guide Reductionist Vaccine Design
Source: PLoS Pathog. 2016 Aug 25;12(8):e1005815. doi: 10.1371/journal.ppat.1005815 (PMC4999182; doi:10.1371/journal.ppat.1005815)
Supplement: S2 Table — Variants in which affinity maturation patches on VRC01 and MinVRC01 were reverted to germline were tested on a cross-clade panel of viruses. While there was loss of neutralization breadth for L-FW3 and H-CDR2 reversions for MinVRC01, neutralization activity remained for VRC01. The data also show that the extra disulfide between H-CDR1 residue 32 and H-CDR3 residue 98 is not necessary for potency and breadth of MinVRC01. Presented values are neutralization IC50 in μg/ml and colored according to the listed legend. (PDF) [file ppat.1005815.s012.pdf]

Table S2. Reversion of affinity maturation patches on VRC01 and MinVRC01 reveal redundancies in affinity maturation residues that contribute to neutralization. Variants in which affinity maturation patches on VRC01 and MinVRC01 were reverted to germline were tested on a cross-clade panel of viruses. While there was loss of neutralization breadth for L-FW3 and H-CDR2 reversions for MinVRC01, neutralization activity remained for VRC01. The data also show that the disulfide is not necessary for potency and breadth of MinVRC01. Presented values are neutralization IC_{50} in µg/ml and colored according to the listed legend.

CLADE	VIRUS	VRC01	VRC01 LΔCDR1	VRC01 LΔFW3	VRC01 HΔCDR1	VRC01 HΔCDR2	VRC01 HΔFW3	VRC01 HΔdisulf
A	Q769	0.02	0.06	0.05	0.03	3.13	0.06	0.029
	0330.v4.c3	0.11	> 50	2.51	1.43	8.50	0.29	0.076
	Q461.e2	0.26	> 50	> 50	50	12.9	0.77	0.239
B	REJO4541.67	0.05	0.11	0.08	0.07	1.35	0.07	0.036
	JR-FL	0.03	> 50	0.24	> 50	> 50	0.10	0.033
	TRO.11	0.33	> 50	> 50	50	> 50	0.78	0.300
C	ZM197M.PB7	0.10	0.19	0.12	0.19	0.30	0.14	0.059
	6240_08_TA5_4622	0.61	> 50	1.37	0.62	10.6	1.23	0.495
	ZM109F.PF4	0.18	> 50	4.53	2.33	2.08	0.36	0.194
C (T/F)	Ce2010_F5	0.28	> 50	0.83	0.33	3.47	0.53	0.166
	Ce2060_G9	0.23	> 50	> 50	> 50	> 50	0.83	0.262
BC	CNE52	0.34	> 50	2.00	0.83	3.17	1.30	0.206
	CNE30	0.50	> 50	> 50	2.47	> 50	1.17	0.433
CRF02_AG	C3347.c11	0.08	0.07	0.12	3.26	> 50	0.16	0.073
	R2184.c04	0.09	0.37	0.10	0.35	0.28	0.20	0.061
G	X2131	0.29	20	0.69	2.40	1.99	1.43	0.467

Neutralization IC_{50} (µg/mL)
50
10
1
0.10
0.01
0.001

CLADE	VIRUS	MinVRC01	MinVRC01 LΔCDR1	MinVRC01 LΔFW3	MinVRC01 HΔCDR1	MinVRC01 HΔCDR2	MinVRC01 HΔFW3	MinVRC01 HΔdisulf
A	Q769	0.02	11.0	> 10	0.41	> 50	0.03	0.031
	0330.v4.c3	0.17	> 50	> 50	> 50	> 50	4.76	0.510
	Q461.e2	0.28	> 50	> 50	> 50	> 50	5.89	0.522
B	REJO4541.67	0.01	> 50	> 10	> 50	> 50	0.03	0.025
	JR-FL	1.19	> 50	> 50	> 50	> 50	> 50	3.030
	TRO.11	0.44	> 50	> 50	> 50	> 50	5.07	1.551
C	ZM197M.PB7	0.03	> 50	0.17	8.90	> 50	0.06	0.066
	6240_08_TA5_4622	0.92	> 50	> 50	> 50	> 50	6.96	1.061
	ZM109F.PF4	0.52	> 50	> 50	> 50	> 50	11.4	0.418
C (T/F)	Ce2010_F5	0.15	> 50	> 50	1.81	> 50	1.39	0.153
	Ce2060_G9	0.09	> 50	> 50	> 50	> 50	0.69	0.153
BC	CNE52	0.09	> 50	> 10	0.72	> 50	1.79	0.141
	CNE30	0.40	> 50	> 50	> 50	> 50	4.92	0.936
CRF02_AG	C3347.c11	0.02	0.02	0.17	0.25	> 50	0.03	0.055
	R2184.c04	0.04	> 50	> 10	3.09	> 50	0.14	0.071
G	X2131	0.18	> 50	> 50	> 50	> 50	2.44	0.145
