# Supplementary material for: Minimally Mutated HIV-1 Broadly Neutralizing Antibodies to Guide Reductionist Vaccine Design
Source: PLoS Pathog. 2016 Aug 25;12(8):e1005815. doi: 10.1371/journal.ppat.1005815 (PMC4999182; doi:10.1371/journal.ppat.1005815)
Supplement: S3 Table — gp120-CD4, PDB ID: 1GC1; gp120-VRC01, PDB ID: 3NGB; gp120-NIH45-46, PDB ID: 4JKP. BSA was calculated using 'Protein interfaces, surfaces and assemblies' service PISA at the European Bioinformatics Institute. (http://www.ebi.ac.uk/pdbe/prot_int/pistart.html) [90]. Areas shaded in blue correspond to elements present in eOD. (PDF) [file ppat.1005815.s013.pdf]

Table S3. Buried Surface Area (BSA) on gp120 calculated from different co-crystal structures with VRC01-class bnAbs. gp120-CD4, PDB ID: 1GC1 ; gp120-VRC01, PDB ID: 3NGB; gp120-NIH45-46, PDB ID: 4JKP. BSA was calculated using 'Protein interfaces, surfaces and assemblies' service PISA at the European Bioinformatics Institute. (http://www.ebi.ac.uk/pdbe/prot_int/pistart.html) (90). Areas shaded in blue correspond to elements present in eOD.

	gp120 - CD4		gp120 - VRC01		eOD-N276 – VRC01		gp120- NIH45-46		BG505 SOSIP – NIH45-46 protomer1		BG505 SOSIP – NIH45-46 protomer2		
	Residue	BSA Å²	Residue	BSA Å²	Residue	BSA Å²	Residue	BSA Å²	Residue	BSA Å²	Residue	BSA Å²	
			D49				D49		E49	13.5	E49		
α1̅			E64				E64		E64		E64	17.5	α1̅
			V65				C65		K65		K65	13.6	
			H66				H66		H66		H66	30.6	
	W96		W96				W96		W96	7.3	W96		
	K97		K97	27.0			K97	56.2	K97	49.0	K97		
α1	D99		N99				N99	18.4	N99	16.8	N99		α1
	E102		E102				E102	39.4	E102	36.6	E102		
	L22		L122				L122	12.3	L122		L122		
β2	T123	35.0	T123				T123		T123		T123		β2
	P124		G124	30.2			G124	33.7	P124		P124		
	L125	91.5							L125		L125		
	N197		N197						N197	15.5	N197		
β3	T198		G198	12.5			G198		T198	14.2	T198		β3
β9	T257		T257		T60		T257	6.9	T257		T257		β9
	V275		E275		V78		E275	7.9	E275	13.7	E275		
	N276		N276	28.9	N79	78.7	N276	9.3	N276	22.4	N276		
	T278		T278	79.5	T81	66.0	T278	44.9	T278	64.0	T278		
Loop D	D279	26.5	N279	62.4	D82	62.3	N279	57.7	N279	50.4	N279		Loop D
	N280	52.5	N280	71.3	N83	64.0	N280	65.7	N280	62.9	N280		
	A281	66.6	A281	70.5	A84	71.8	A281	84.1	A281	81.2	A281		
	K282		K282	29.5	K85	41.8	K282	46.6	K282	65.4	K282		
	T283	21.0	T283	13.4	S86		T283	8.8	N283	19.5	N283		
	I360		I360		I134		I360		R360	16.2	R360		
	S365	69.2	S365	60.3	S139	54.7	S365	52.6	S365	65.0	S365		
β15	G366	28.5	G366	22.8	G140	24.8	G366	24.0	G366	24.7	G366		β15
	G367	34.2	G367	24.2	G141	20.6	G367	25.4	G367	22.8	G367		
	D368	57.1	D368	49.2	D142	36.7	D368	55.5	D368	44.6	D368		
α3	E370	16.5	E370		E144	6.8	E370	18.1	E370		E370		α3
	I371	43.9	I371	48.0	I145	32.6	I371	39.7	V371	39.0	V371		
β20	N425	19.2	N425				N425	10.8	N425		N425		β20
	M426	16.1	M426				M426	4.0	M426		M426		
	W427	29.2	W427	5.5			W427	40.3	W427	10.6	W427		
	Q428	1.0	Q428				Q428		Q428	87.3	Q428		
	K429	9.5	K429	2.1			G429	25.7	R429		R429		
β21	V430	119.1	T430	57.0			T430	50.6	I430	37.7	I430		β21
	G431		G431				G431	11.3	G431				
	K432		Q432				Q432	11.2	Q432				
β23	T455	17.7	T455	32.7	T25	23.3	T455	32.0	T455	27.7	T455		β23
	R456	4.2	R456	5.6	R26	10.7	R456	8.4	R456	13.1	R456		
	D457	21.5	D457	43.8	D27	46.8	D457	37.2	D457	53.8	D457		
	G458	20.4	G458	52.2	G28	52.1	G458	42.0	G458	42.8	G458		
	G459	47.8	G459	70.3	G29	66.0	G459	69.1	G459	68.9	G459		
	N460		A460	53.0	N30	99.4	A460	24.4	S460	21.1	S460		
V5	S461		N461	69.1	S31		N461	37.2	T461	98.3	T461		V5
	N462		N462		N32		N462		N462		N462		
	N463		T463	15.9	A33	6.1	T463	17.1	S463	12.4	S463		
	S465		N465	7.6	S35	21.5	N465	6.3	T465	15.0	T465		
β24	E466		E466	13.1	E36	4.7	E466		E466		E466		β24
	I467		T467	9.0	I37	23.7	T467	18.6	T467	14.9	T467		
	R469	15.0	R469	24.3	R39	18.1	R469	25.6	R469	24.3	R469		
	G471		G471		G41		G471		G471		G471		
	G472	19.4	G472		G42	13.1	G472		G472	12.2	G472		
	G473	26.7	G473	46.8	G43	27.8	G473	35.7	G473	19.8	G473		
	D474	50.6	N474	23.6	D44	62.9	N474	24.0	D474	28.9	D474		
	M475		I475		M45	71.3	I475		M475		M475		
α5	R476	7.0	K476	4.8	R46	87.2	K476	23.0	R476	39.0	R476		α5
	D477		D477		D47	5.4	D477		D477		D477		
	R480		R480		I48	8.7	R480	12.7	R480	15.6	R480		
**Total protein BSA (Å²)**		978.7		1166.1		1214.9		1274.4		1388.1		61.7	
**Glycan**	197		197		197		197		197	126.0	197		
	234		234		234		234		234	284.2	234		
	262		262		262		262		262		262	21.8	
	276	33.1	276	108.2	276	503.2	276	386.9	276	64.2	276		
	462		462		462		462		462	135.1	462		
**Total glycan BSA (Å²)**		33.1		108.2		503.2		386.9		609.5		21.8	
**Total BSA monomer (Å²)**		1011.8		1274.3		1718.1		1661.3		1997.6		83.5	
**Total BSA trimer (Å²)**										2081.1			
