# Supplementary material for: Minimally Mutated HIV-1 Broadly Neutralizing Antibodies to Guide Reductionist Vaccine Design
Source: PLoS Pathog. 2016 Aug 25;12(8):e1005815. doi: 10.1371/journal.ppat.1005815 (PMC4999182; doi:10.1371/journal.ppat.1005815)
Supplement: S4 Table — MinVRC01 patch revertants were tested for neutralization on BG505 mutant viruses. (Top) Values are neutralization IC50 in μg/ml and colored according to the listed legend. (Below) Values are fold-enhancement in neutralization potency compared to wild-type virus. LT, low titer. NA, not available due to low titer. (PDF) [file ppat.1005815.s014.pdf]

**Table S4. BG505 glycan mutants.** MinVRC01 reverted variant antibodies were tested for neutralization on BG505 mutant viruses. (Top) Presented values are neutralization $IC_{50}$ in μg/ml and colored according to the listed legend. (Below) Presented values are fold-enhancement in neutralization potency compared to wild-type virus.  LT, low titer. NA, not available due to low titer.

| | MinVRC01 GL-L | MinVRC01 | MinVRC01 LΔCDR1 | MinVRC01 LΔFW3 | MinVRC01 HΔCDR1 | MinVRC01 HΔCDR2 | MinVRC01 HΔF54 | MinVRC01 HΔFW3 | MinVRC01 HΔdisulph | VRC01 | PGT151 | 12A12 |
|---|---|---|---|---|---|---|---|---|---|---|---|---|
| WT (T332N) | > 10 | 0.082 | > 10 | > 10 | > 10 | > 10 | 0.316 | 0.596 | 0.053 | 0.039 | 0.014 | 0.02 |
| N197A | > 10 | 0.005 | > 10 | > 10 | 0.435 | > 10 | 0.012 | 0.031 | 0.005 | 0.003 | 0.016 | 0.001 |
| P206A | > 10 | 0.07 | > 10 | > 10 | > 10 | > 10 | 0.514 | 1 | 0.075 | 0.031 | 0.011 | 0.006 |
| K207A | > 10 | 0.092 | > 10 | > 10 | > 10 | > 10 | 0.746 | 0.13 | 0.045 | 0.012 | 0.008 | 0.006 |
| N234A | > 10 | 0.026 | > 10 | 2 | 2 | > 10 | 0.042 | 0.053 | 0.016 | 0.003 | 0.005 | 0.009 |
| N262A | > 10 | 0.041 | > 10 | > 10 | > 10 | > 10 | 0.09 | 0.154 | 0.046 | 0.014 | 0.014 | 0.004 |
| N276A | 0.038 | 0.013 | 0.022 | 0.016 | 0.561 | > 10 | 0.032 | 0.07 | 0.01 | 0.006 | 0.012 | 0.002 |
| N301A | > 10 | 0.044 | > 10 | > 10 | > 10 | > 10 | 0.148 | 1 | 0.054 | 0.014 | 0.025 | 0.009 |
| R304A | > 10 | 0.112 | > 10 | > 10 | > 10 | > 10 | 0.542 | 1 | 0.138 | 0.082 | 0.037 | 0.032 |
| K305A | > 10 | 0.178 | > 10 | > 10 | > 10 | > 10 | 0.605 | 1 | 0.185 | 0.107 | 0.055 | 0.035 |
| R308A | > 10 | 0.083 | > 10 | > 10 | > 10 | > 10 | 0.389 | 1 | 0.116 | 0.052 | 0.022 | 0.015 |
| T332N | > 10 | 0.082 | > 10 | > 10 | > 10 | > 10 | 0.316 | 1 | 0.053 | 0.039 | 0.014 | 0.02 |
| N332T | > 10 | 0.058 | > 10 | > 10 | > 10 | > 10 | 0.195 | 1 | 0.109 | 0.051 | 0.026 | NA |
| N386A | > 10 | 0.037 | > 10 | > 10 | ~ 10 | > 10 | 0.168 | 1 | 0.033 | 0.026 | 0.017 | 0.006 |
| N392A | > 10 | 0.054 | > 10 | > 10 | > 10 | > 10 | 0.113 | 1 | 0.066 | 0.024 | 0.013 | 0.008 |
| N425A | > 10 | 0.056 | > 10 | > 10 | > 10 | > 10 | 0.871 | 1 | 0.063 | 0.02 | 0.009 | 0.015 |
| M426A | > 10 | 0.077 | > 10 | > 10 | > 10 | > 10 | 0.428 | 1 | 0.072 | 0.027 | 0.014 | 0.048 |
| W427A | LT | LT | LT | LT | LT | LT | LT | LT | LT | LT | LT | LT |
| Q428A | 2.72 | 0.01 | ~ 10 | 2 | 0.126 | > 10 | 0.117 | 0.047 | 0.006 | 0.003 | 0.004 | 0.012 |
| R429A | > 10 | 0.081 | > 10 | > 10 | > 10 | > 10 | 0.802 | 0.4 | 0.109 | 0.028 | 0.012 | 0.016 |
| Q432A | > 10 | 0.071 | > 10 | > 10 | ~ 10 | > 10 | 0.447 | 0.459 | 0.097 | 0.035 | 0.015 | 0.015 |
| N462A | > 10 | 0.049 | > 10 | > 10 | > 10 | > 10 | 0.312 | ~ 10 | 0.066 | 0.026 | 0.014 | 0.005 |

| Neutralization $IC_{50}$ (μg/mL) | 10 | 1 | 0.1 | 0.01 | 0.001 |
|---|---|---|---|---|---|

| | MinVRC01 GL-L | MinVRC01 | MinVRC01 LΔCDR1 | MinVRC01 LΔFW3 | MinVRC01 HΔCDR1 | MinVRC01 HΔCDR2 | MinVRC01 HΔF54 | MinVRC01 HΔFW3 | MinVRC01 HΔdisulph | VRC01 | PGT151 | 12A12 |
|---|---|---|---|---|---|---|---|---|---|---|---|---|
| N197A | 1 | 17 | 1 | 1 | 23 | 1 | 26 | 19 | 11 | 11 | 0.9 | 18 |
| P206A | 1 | 1.2 | 1 | 1 | 1 | 1 | 0.6 | 0.6 | 0.7 | 1.2 | 1.3 | 3.1 |
| K207A | 1 | 0.9 | 1 | 1 | 1 | 1 | 0.4 | 4.6 | 1.2 | 3.3 | 1.7 | 3.5 |
| N234A | 1 | 3.1 | 1 | 5 | 5 | 1 | 7.5 | 11 | 3.4 | 14 | 3.2 | 2.2 |
| N262A | 1 | 2 | 1 | 1 | 1 | 1 | 3.5 | 3.9 | 1.2 | 2.9 | 1 | 4.9 |
| N276A | 260 | 6.3 | 455 | 620 | 18 | 1 | 10 | 8.5 | 5 | 6 | 1.2 | 13 |
| N301A | 1 | 1.9 | 1 | 1 | 1 | 1 | 2.1 | 0.6 | 1 | 2.7 | 0.6 | 2.2 |
| R304A | 1 | 0.7 | 1 | 1 | 1 | 1 | 0.6 | 0.6 | 0.4 | 0.5 | 0.4 | 0.6 |
| K305A | 1 | 0.5 | 1 | 1 | 1 | 1 | 0.5 | 0.6 | 0.3 | 0.4 | 0.3 | 0.6 |
| R308A | 1 | 1 | 1 | 1 | 1 | 1 | 0.8 | 0.6 | 0.5 | 0.7 | 0.7 | 1.4 |
| T332N | 1 | 1 | 1 | 1 | 1 | 1 | 1 | 0.6 | 1 | 1 | 1 | 1 |
| N332T | 1 | 1.4 | 1 | 1 | 1 | 1 | 1.6 | 0.6 | 0.5 | 0.8 | 0.6 | ND |
| N386A | 1 | 2.2 | 1 | 1 | 1 | 1 | 1.9 | 0.6 | 1.6 | 1.5 | 0.9 | 3.5 |
| N392A | 1 | 1.5 | 1 | 1 | 1 | 1 | 2.8 | 0.6 | 0.8 | 1.6 | 1.1 | 2.5 |
| N425A | 1 | 1.5 | 1 | 1 | 1 | 1 | 0.4 | 0.6 | 0.8 | 1.9 | 1.6 | 1.3 |
| M426A | 1 | 1.1 | 1 | 1 | 1 | 1 | 0.7 | 0.6 | 0.7 | 1.5 | 1 | 0.4 |
| W427A | NA | NA | NA | NA | NA | NA | NA | NA | NA | NA | NA | NA |
| Q428A | 3.7 | 8.1 | 1 | 5 | 79 | 1 | 2.7 | 13 | 8.2 | 13 | 3.5 | 1.7 |
| R429A | 1 | 1 | 1 | 1 | 1 | 1 | 0.4 | 1.5 | 0.5 | 1.4 | 1.2 | 1.3 |
| Q432A | 1 | 1.2 | 1 | 1 | 1 | 1 | 0.7 | 1.3 | 0.6 | 1.1 | 0.9 | 1.4 |
| N462A | 1 | 1.7 | 1 | 1 | 1 | 1 | 1 | 17 | 0.8 | 1.5 | 1 | 4.3 |
