# Supplementary material for: Minimally Mutated HIV-1 Broadly Neutralizing Antibodies to Guide Reductionist Vaccine Design
Source: PLoS Pathog. 2016 Aug 25;12(8):e1005815. doi: 10.1371/journal.ppat.1005815 (PMC4999182; doi:10.1371/journal.ppat.1005815)
Supplement: S5 Table — The glycan site at N276 was removed by alanine mutagenesis (N276A) on a cross-clade 13-virus panel and tested for neutralization compared to the corresponding virus without the glycan site removed (wild-type, WT). Virus panels were tested for neutralization by chimeric VRC01-class antibodies containing a mature heavy chain and germline light chain. Values are neutralization IC50 in μg/ml and colored according to the legend. (PDF) [file ppat.1005815.s015.pdf]

Table S5. Light chain of VRC01-class antibodies undergoes somatic hypermutation to accommodate the N276 glycan on Env. The glycan site at N276 was removed by alanine mutagenesis (N276A) on a cross-clade 13-virus panel and tested for neutralization compared to the corresponding virus without the glycan site removed (wild-type, WT). Virus panels were tested for neutralization by chimeric VRC01-class antibodies containing a mature heavy chain and germline light chain. Presented values are neutralization IC_{50} in µg/ml and colored according to the listed legend.

			Mature heavy chain/germline light chain chimeras									
	CLADE	VIRUS	VRC03	CHA31	PGV04	NIH45-46	PGV19	3BNC60	12A12	12A21	VRC01	PGV20
WT	A	Q769.d22	0.077	> 50	> 50	0.032	0.185	0.575	1.09	0.08	0.234	0.019
WT	A	191084 B7-19	> 50	> 50	> 50	0.053	> 50	> 50	> 50	> 50	0.147	0.033
WT	B	SC422661.8	> 50	> 50	> 50	2.27	> 50	> 50	> 50	> 50	3.60	5.06
WT	B	RHPA4259.7	> 50	> 50	> 50	> 50	> 50	> 50	> 50	> 50	> 50	> 50
WT	B (T/F)	1012_11_TC21_3257	0.030	> 50	> 50	0.008	> 50	> 50	> 50	> 50	0.175	0.041
WT	B (T/F)	6244_13_B5_4576	> 50	> 50	> 50	1.27	> 50	> 50	> 50	> 50	> 50	> 50
WT	C	ZM249M.PL1	> 50	> 50	> 50	> 50	> 50	> 50	> 50	> 50	> 50	3.97
WT	C	ZM109F.PB4	> 50	> 50	> 50	> 50	> 50	> 50	> 50	> 50	> 50	> 50
WT	C	249M B10	> 50	> 50	> 50	> 50	> 50	> 50	1.56	> 50	3.81	0.114
WT	G	X1632_S2_B10	> 50	> 50	> 50	> 50	> 50	> 50	> 50	> 50	> 50	> 50
WT	AC	3301.v1.c24	> 50	> 50	> 50	0.123	> 50	> 50	> 50	> 50	> 50	0.870
WT	CD	6952.v1.c20	> 50	> 50	> 50	4.52	> 50	> 50	> 50	> 50	3.29	> 50
WT	ACD	0815.v3.c3	> 50	> 50	> 50	> 50	> 50	2.26	> 50	> 50	> 50	0.286

Neutralization IC_{50} (µg/mL)
50
10
1
0.10
0.01
0.001

	CLADE	VIRUS	VRC03	CHA31	PGV04	NIH45-46	PGV19	3BNC60	12A12	12A21	VRC01	PGV20
N276A	A	Q769.d22	0.008	0.291	> 50	0.003	0.010	0.015	0.057	0.003	0.008	0.002
N276A	A	191084 B7-19	0.006	0.010	0.011	0.002	0.009	0.034	0.089	1.376	0.011	0.004
N276A	B	SC422661.8	0.003	0.033	0.010	0.003	0.118	0.043	3.095	> 50	0.017	0.007
N276A	B	RHPA4259.7	0.023	0.269	0.272	0.002	> 50	0.015	> 50	> 50	0.010	0.109
N276A	B (T/F)	1012_11_TC21_3257	0.005	0.134	0.012	0.001	0.020	0.023	0.786	0.269	0.015	0.007
N276A	B (T/F)	6244_13_B5_4576	0.101	1.016	> 50	0.009	1.504	0.059	0.332	0.098	0.044	0.012
N276A	C	ZM249M.PL1	0.178	0.033	0.090	0.006	0.196	0.266	0.057	1.230	0.008	0.002
N276A	C	ZM109F.PB4	> 50	> 50	0.088	0.028	> 50	> 50	> 50	> 50	0.286	0.005
N276A	C	249M B10	0.148	0.020	0.055	0.006	0.159	0.187	0.050	1.010	0.010	0.001
N276A	G	X1632_S2_B10	0.059	0.008	0.005	0.003	> 50	0.029	> 50	> 50	0.035	0.003
N276A	AC	3301.v1.c24	0.089	0.627	0.176	0.004	1.855	0.218	0.265	> 50	0.033	0.004
N276A	CD	6952.v1.c20	0.008	> 50	> 50	0.004	1.766	> 50	> 50	> 50	0.004	0.004
N276A	ACD	0815.v3.c3	0.001	0.001	> 50	0.001	0.002	0.002	0.017	0.054	0.007	0.001
