# Supplementary material for: Minimally Mutated HIV-1 Broadly Neutralizing Antibodies to Guide Reductionist Vaccine Design
Source: PLoS Pathog. 2016 Aug 25;12(8):e1005815. doi: 10.1371/journal.ppat.1005815 (PMC4999182; doi:10.1371/journal.ppat.1005815)
Supplement: S6 Table — The glycan site at N276 was removed by alanine mutagenesis on a cross-clade 6-virus panel and tested for neutralization by VRC01 class antibodies as well as CD4 IgG2, other CD4bs bnAbs (b12), and CD4bs non-broadly neutralizing antibodies (b6, F105). Values are neutralization IC50 in μg/ml and colored according to the legend. (PDF) [file ppat.1005815.s016.pdf]

Table S6. Removal of the N276 glycan site affects predominantly VRC01-class antibodies. The glycan site at N276 was removed by alanine mutagenesis on a cross-clade 6-virus panel and tested for neutralization by VRC01 class antibodies as well as CD4 IgG_2, other CD4bs bnAbs (b12), and CD4bs non-broadly neutralizing antibodies (b6, F105). Presented values are neutralization IC_{50} in µg/ml and colored according to the listed legend.

WT

	JR-CSF	92TH021	IAVI C22	94UG103	92RW020	JR-FL
VRC01	0.225	0.370	0.651	0.164	0.205	0.021
MinVRC01	0.080	0.539	0.899	0.483	0.151	0.367
MinVRC01 LΔFW3	> 50	> 50	> 50	> 50	> 50	> 50
MinVRC01 GL-L	> 50	> 50	> 50	> 50	> 50	> 50
b6	> 50	> 50	> 50	> 50	> 50	> 50
F105	> 50	> 50	> 50	> 50	> 50	> 50
CD4 IgG2	1.11	0.325	5.16	0.044	0.434	0.045
b12	0.590	1.14	8.55	2.02	> 50	0.019

N276A

	JR-CSF	92TH021	IAVI C22	94UG103	92RW020	JR-FL
VRC01	0.063	0.149	0.069	0.026	0.040	0.026
MinVRC01	0.017	0.048	0.051	0.117	0.033	0.004
MinVRC01 LΔFW3	0.053	0.038	0.097	0.149	0.055	0.006
MinVRC01 GL-L	0.057	0.292	0.163	0.215	0.073	0.009
b6	> 50	> 50	> 50	> 50	> 50	> 50
F105	> 50	> 50	> 50	> 50	> 50	> 50
CD4 IgG2	5.20	0.675	19.3	0.166	0.815	0.050
b12	0.738	3.75	11.3	8.00	> 50	0.016

Neutralization IC_{50} (µg/mL)
50
10
1
0.10
0.01
0.001
