# Supplementary material for: Minimally Mutated HIV-1 Broadly Neutralizing Antibodies to Guide Reductionist Vaccine Design
Source: PLoS Pathog. 2016 Aug 25;12(8):e1005815. doi: 10.1371/journal.ppat.1005815 (PMC4999182; doi:10.1371/journal.ppat.1005815)
Supplement: S7 Table — (PDF) [file ppat.1005815.s017.pdf]

Table S7. X-ray data collection and refinement statistics

	eOD-N276Kif + VRC01	BG505 SOSIP + PGT122 + NIH45-46
Beamline	APS 23-ID-B	APS 23-ID-D
# crystals	1	3
Wavelength, Å	1.03324	1.03324
Space group	$P4_12_12$	C2
Unit cell a, b, c (Å)	113.5, 113.5, 412.5	153.7, 254.2, 283.4
α, β, γ (°)	90, 90, 90	90, 101.0, 90
Resolution (Å)	40.0 – 3.25 (3.35 – 3.25)	40.0 – 4.4 (4.5 – 4.4)
Completeness	99.9 (100.0)	99.9 (100.0)
Redundancy	8.0 (8.2)	21.4 (21.3)
No. total reflections	351,738	1,451,956
No. unique reflections	43,730	67,706
I/σ	10.9 (2.2)	5.9 (2.0)
$CC_{1/2}$	99.5 (57.2)	98.7 (13.5)
R_{sym}	0.16 (0.85)	0.39 (1.03)
R_{pim}	0.06 (0.32)	0.09 (0.23)
Refinement Statistics		
Resolution (Å)	40 - 3.25	40 - 4.4
No. reflections total/R_{free}	43,600/2,180	67,696/1,021
R_{cryst}/R_{free}	0.22/0.27	0.29/0.32
RMSD bond length (Å)	0.004	0.004
RMSD bond angles (°)	0.8	0.8
Protein atoms/Glycan atoms	13,500/349	28,692/2,682
Wilson B-value ($Å^2$)	100	145
Overall average B-value ($Å^2$)	120	218
Ramachandran allowed %	99.4	99.2
MolProbity all-atom clashscore	9.2	8.1
PDB ID	5KZC	5D9Q
